# Viable strategies for enhancing performance in ball sports by mitigating mental fatigue: A systematic review

**DOI:** 10.1371/journal.pone.0313105

**Published:** 2024-11-08

**Authors:** Xiaoyang Pan, Kim Geok Soh, Kim Lam Soh

**Affiliations:** 1 Department of Sport Studies, Faculty of Education Studies, Universiti Putra Malaysia, Selangor, Malaysia; 2 Department of Nursing, Faculty of Medicine and Health Sciences, Universiti Putra Malaysia, Selangor, Malaysia; Huashan Hospital Fudan University, CHINA

## Abstract

Ball sports demand precise control of body and ball while executing tactics and team coordination, which leads to cognitive depletion and mental fatigue. The detrimental effects of mental fatigue on physical, technical, cognitive, and tactical performance in ball sports are evident, highlighting the need for effective management of mental fatigue as a crucial component of modern sports science. This review aims to systematically study and integrate existing experiments on mental fatigue recovery interventions to identify viable strategies for mitigating its impacts. Following PRISMA guidelines, electronic databases including Web of Science, PubMed, SPORTDiscus, and Scopus were used for literature screening. Studies that induced mental fatigue followed by interventions aimed at mental fatigue recovery were included in this review, resulting in 6 articles primarily focused on basketball, soccer, and golf. Short-term interventions such as music listening, mindfulness training, self-talk, and natural visual stimuli have been shown as effective strategies to alleviate mental fatigue and enhance technical and cognitive performance in ball sports. However, their practical application in real-game environments requires further research and validation. Additionally, future research should explore defensive skills and tactical performance as viable directions for study.

## Introduction

In the fiercely competitive world of sports, athletes’ performance depends not only on their physical fitness and technical skills but also critically on their psychological state [1]. On one hand, ball sports with their fast pace require coordination and teamwork, posing psychological challenges [2, 3]. On the other hand, these ball sports require athletes to exercise dual control over their bodies and the ball, which increases the cognitive load and leads to mental fatigue (MF). When the brain experiences pressure, fatigue, and stress, it directly impacts athletic performance negatively, affecting various aspects [4]. For instance, MF leads to decreased physical performance [4, 5], adversely affects visual motion performance [6], and diminishes endurance running [7, 8]. Moreover, it hampers technical skills [9], evidenced by studies showing reduced serve speed and accuracy in tennis players under MF [10, 11]. MF also significantly impairs golf putting accuracy [12], basketball shooting accuracy [13–15], soccer passing and shooting accuracy [16–19], and tackling techniques in rugby players [20]. It also impedes cognitive performance [21], reducing athletes’ psychomotor vigilance [22], decision speed, reaction time [23, 24], and accuracy [25–28]. Tactical execution is also affected, as MF influences strategic decisions among soccer players [29]. Silva et al. [30] found that psychologically fatigued soccer players execute fewer actions in both offensive and defensive phases, highlighting its impact on sports tactical management. Summarizing findings from systematic reviews, MF unfavorably affects physical, technical, cognitive, and tactical performances in ball sports [31, 32]. These examples underscore the widespread and profound effects of MF on ball sport athletes.

Managing MF is becoming a crucial component of sports science [33]. Given the intensity and pressure of competitive matches in ball sports, addressing MF is paramount [34]. Athletes who can effectively manage MF can demonstrate higher cognitive abilities, which directly impacts their attention and performance [29, 35]. Thus, techniques that reduce cognitive load or manage MF are crucial for athletes’ achievements during competitions [13, 36]. The primary aim of this systematic review is to study and synthesize existing intervention experiments on MF recovery, identifying viable strategies to mitigate its impact. It explores and compiles proven techniques and interventions that alleviate MF. The review aims to provide practical guidelines for coaches, trainers, and sports psychologists to implement MF management strategies in training and competitive environments. Through these objectives, this review seeks to comprehensively understand MF in ball sports and offer actionable insights to enhance athletic performance.

## Methods

The review’s reporting adheres to the Preferred Reporting Items for Systematic Reviews and Meta-Analyses (PRISMA) checklist (**S2 Table**) [37].

### Search strategy

Electronic databases such as Web of Science, PubMed, SPORTDiscus, and Scopus were systematically searched up to January 2, 2024. Boolean operators AND and OR were applied with the specified keywords which can refer to **Table 1** below. A uniform search procedure was applied to all databases and did not use the automated functions (**S1 Table**).

**Table 1:** Search string.

Search Builder	Search String
Mental Fatigue	"mental fatigue" OR "cognitive fatigue" OR "mental effort" OR "cognitive effort" OR "mental exertion" OR "ego depletion"
Performance	"performance" OR "decision making" OR "skill" OR "technique"
Ball sports	"3x3 Basketball" OR "Badminton" OR "Baseball" OR "Softball" OR "Basketball" OR "Beach Handball" OR "Beach Volleyball" OR "Cricket" OR "Flag Football" OR "Football" OR "Futsal" OR "Golf" OR "Handball" OR "Hockey" OR "Ice Hockey" OR "Lacrosse" OR "Rugby Sevens" OR "Squash" OR "Table Tennis" OR "Tennis" OR "Volleyball" OR "Water Polo"

### Criteria

Literature screening was performed using the PICO method (**Table 2**). Studies had to meet the following inclusion criteria: (P) the sample population included players; (I) interventions that induced MF followed by recovery; (C) interventions that induced MF followed by no recovery; and (O) outcomes included any form of athletic performance.

**Table 2:** PICO criteria for inclusion criteria.

Items	Detailed inclusion criteria
Population	Players
Intervention	Recovery after induced MF
Comparison	No recovery after induced MF
Outcome	Any form of performance

### Study selection

**Fig 1:**
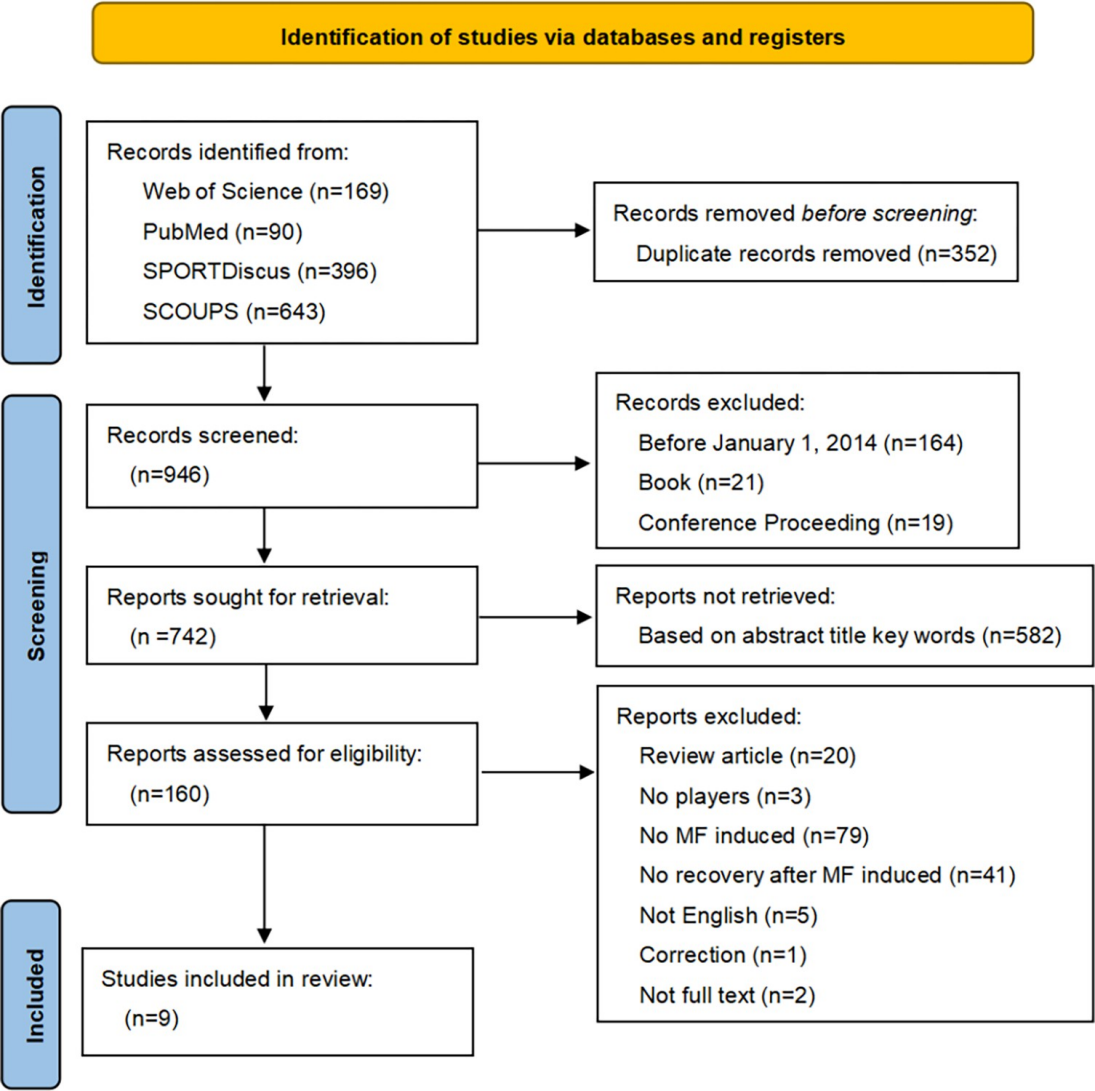
PRISMA flow diagram.

The screening of the literature followed the PRISMA flowchart, where the number of excluded articles and the reasons are labeled (**Fig 1**). The process of identification and screening was co-written by two researchers. When a disagreement arises between the two researchers, the opinion of a third reviewer is sought and negotiated to reach a consensus.

### Quality assessment

“QualSyst” was used to assess the methodology quality [38]. It contained 14 items (**Table 3**). The score was set according the degree to which the certain criteria were met (yes = 2, partial = 1, no = 0). “N/A” was marked when the items did not apply to the study design and excluded from the total calculation of score. A score of ≥75% indicated strong quality, a score of 55–75% indicated moderate quality, and a score of ≤55% indicated weak quality.

**Table 3:** Assessing the quality of studies.

Items	Ⅰ	Ⅱ	Ⅲ	Ⅳ	Ⅴ	Ⅵ	Ⅶ	Ⅷ	Ⅸ	Ⅹ	XI	XII	XIII	XIV	Rating
Englert & Bertrams, 2016 [39] (2016)	2	2	2	2	2	0	0	2	2	2	2	0	2	2	Strong
Shaabani et al., 2020 [40] (2020)	2	2	2	2	2	0	1	2	2	2	2	0	2	2	Strong
Sun et al., 2022 [41] (2022)	2	2	2	2	2	0	1	2	2	2	2	0	2	2	Strong
Fortes et al., 2022 [42] (2022)	2	2	2	2	2	2	2	2	2	2	2	0	2	2	Strong
Galanis et al., 2022 [12] (2022) Exp. 1	2	2	1	2	2	0	0	2	2	2	2	0	2	2	Strong
Moreira et al., 2023 [43] (2023)	2	2	2	2	0	2	2	2	0	2	2	0	2	2	Strong

I, description of the problem; II, appropriate study design; III, appropriate subject selection; IV, characterization; V, random assignment; VI, blinding of researcher; VII, blinding of subjects; VIII, outcome measures are clear; IX, sample sizes are appropriate; X, analytic methods are well described; XI,estimate of variance reported; XII, control for confounding; XIII, detailed report; XIV, conclusions supported by results. N/A, not applicable; 2, for yes; 1, for partial; 0, for no.

## Results

This study screened 1298 articles, and the reasons for exclusion are labeled in the PRISMA flowchart (**Fig 1**). The literature screening resulted in a total of 6 articles being included in the review (**Table 4**). The assessment of article quality showed that all studies were of high quality (**Table 3**).

**Table 4:** Characteristics of the studies.

Authors (Year)	Ball sports	Population	Characteristics	MF induced	MF assessment	Recovery intervention	Comparison	Study designs	Outcome
Englert & Bertrams, (2016) [39]	Basketball	33M; 6FM	Professional; [Age = 24.41, ±2.51]	Transcribing text [6 min]	4-point Likert-type scales	Listened to a relaxing song [2min]	Without relaxing song break [2min]	RCTs	Ego depletion ↓ in I vs. C Free throw percentage ↑ in I vs. C
Shaabani et al. (2020) [40]	Basketball	72M	Semi-professional; [Age = 28.6, ±4.0]	Stroop color-word task [15 min]	7-point Likert-type scales	Listen to audio mindfulness training [15 min]	Listen to a natural history [15 min]	RCTs	Ego depletion ↓ in I vs. C Basketball shooting score ↑ in I vs. C
Sun et al. (2022) [41]	Soccer	40M	Semi-professiona; [Age = 20.73 ± 2]	Stroop color-word task [45 min]	Visual analog scale	Watched natural photos [4.17 min; 8.33 min; 12.50 min]	Watched urban photos [4.17 min; 8.33 min; 12.50 min]	RCTs	MF ↓ in I vs. C Decision-making reaction time ↓ in I vs. C Decision-making reaction accuracy ↑ in I vs. C
Fortes et al. (2022) [42]	Basketball	20M	Professional; [Age = 24.77, ±4.2]	Play sports-based video games for prolonged periods [60 min]	Visual analog scale	a-tDCS [30 min]	Sham [30 min]	RCTs, Crossover	MF ↓ in I vs. C Accuracy of visuomotor → in I vs. C Response time of the visuomotor ↓ in I vs. C Accuracy and response time in basketball decision-making ↓ in I vs. C
Galanis et al. (2022) Exp. 1 [12]	Golf	32M; 30FM	Beginner; [Age = 18.58, ± 1.03]	Attention Task [15 min]	7-point Likert-type scales	Self-Talk [During the test]	No self-talk [During the test]	RCTs	Ego depletion ↓ in I vs. C Attention ↑ in I vs. C Putting performance ↑ in I vs. C
Galanis et al. (2022) Exp. 2 [12]	Golf	27M; 27FM	Beginner; [Age = 19.91, ±1.04]	Attention Task [15 min]	7-point Likert-type scales	Self-Talk [During the test]	No self-talk [During the test]	RCTs	Ego depletion ↓ in I vs. C Attention ↑ in I vs. C Putting performance ↑ in I vs. C
Moreira et al. (2023) [43]	Basketball	8FM	Professional; [Age = 25, ±8]	Stroop color-word task [30 min]	Task Load Index	a-tDCS [20 min]	Sham [20 min]	nRCTs, Crossover	MF → in I vs. C Mean accuracy → in I vs. C Shooting performance → in I vs. C

M, male; F, female; I, intervention; C, control; RCT, randomized controlled trial; MF, mental fatigue; ↑, increase; ↓, decrease; →, unchanged.

### General study characteristics

6 articles included a total of 295 participants. The sample sizes ranged from as few as 8 participants [43] to as many as 72 participants [40]. Most studies included only male participants [40–43], but 2 studies included both genders [12, 39], and only one study focused exclusively on females [43]. Among all participants, 24.07% were female and 75.93% were male. Most studies focused on professional [39, 42, 43] or semi-professional [40, 41] participants, with only one study focusing on beginners [12].

The search used keywords for all 24 Olympic ball sports, making it more representative and comprehensive than previous searches of ball sports. However, only three types of ball sports met the screening criteria: basketball, soccer, and golf. Basketball studies were the most numerous, with four [39, 40, 42, 43]. Soccer [41] and golf [12] each had only one study.

### MF intervention and assessment

In this study, the Stroop task was the most frequently chosen tool for inducing MF. Shaabani et al. [40] induced MF through a 15-minute Stroop color-word task. Moreira et al. [43] induced MF through a 30-minute Stroop color-word task. Sun et al. [41] induced MF through a 45-minute Stroop color-word task. In these Stroop tasks, different durations were proven to be effective, with the shortest being 15 minutes and the longest being 45 minutes. Galanis et al. [12] in both Experiment 1 and Experiment 2 induced MF through a 15-minute computer-based attention task. In this study, Fortes et al. [42] used the longest duration to induce MF by having participants play sports-based video games for 60 minutes. Englert & Bertrams [39] used the shortest duration to induce MF by having participants transcribe text for 6 minutes.

In the studies included in this research, subjective assessments were used to measure MF, including Likert-type scales [12, 39, 40], visual analog scale [41, 42], and task load index [43]. Englert & Bertrams [39] employed a 4-point Likert-type scale ranging from 1 (not at all) to 4 (very much). Shaabani et al. [40] and Galanis et al. [12] utilized 7-point Likert-type scales ranging from 1 (not at all) to 7 (very). Sun et al. [41] and Fortes et al. [42] used visual analog scales where participants moved a vertical line along a 100-mm horizontal line to directly indicate their level of MF, with "0" indicating "none at all" and "100" indicating "maximal fatigue." Moreira et al. [43] used the National Aeronautics and Space Administration’s Task Load Index (NASA-TLX) [44] to assess the subjective workload related to participation in the Stoop task.

### Viable strategies to mitigate MF and improve performance

Englert & Bertrams [39] found that professional basketball players significantly reduced feelings of ego depletion, increased relaxation levels, and improved free throw accuracy by listening to relaxing music. Shaabani et al. [40] demonstrated that mindfulness training helped semi-professional basketball players alleviate MF and enhance shooting performance. Fortes et al. [42] and Moreira et al. [43] investigated the effects of transcranial direct current stimulation (tDCS) on MF and performance among basketball players, showing improved decision-making accuracy and faster response times [42], but no significant improvement in shooting performance [43]. Galanis et al. [12] emphasized the positive impact of self-talk on MF and attention among beginner golfers, demonstrating enhanced putting performance during the task. Sun et al. [41] found that exposure to natural visual stimuli helped alleviate MF and enhance decision-making abilities in soccer players. Most studies focused on offensive performance, such as basketball shooting [39, 40, 43] and golf putting performance [12]. While Fortes et al. [42] studied shooting decision-making, which falls under offensive performance, Sun et al. [41] studied soccer decision-making, involving both offensive and defensive aspects. No studies specifically focused on defensive performance.

Regarding intervention durations reported to effectively mitigate MF and enhance performance, the shortest intervention duration reported was 2 minutes for listening to relaxing music [39], while the longest was 15 minutes for mindfulness training [40]. Galanis et al. [12] did not specify a particular intervention duration but emphasized continuous self-talk during the task. Among the reviewed studies, Sun et al. [41] investigated the effects of different intervention durations (4.17 minutes; 8.33 minutes; 12.50 minutes) on alleviating MF in athletes, with the longest duration (12.50 minutes) significantly improving decision-making response times.

## Discussion

### Viable strategies to mitigate MF and improve performance

MF negatively impacts physical, technical, cognitive, and tactical performance in ball sports [31, 45–48]. However, this systematic review reveals that most existing studies focus on alleviating MF to improve technical performance [12, 39, 40, 43], with a lack of research on enhancing tactical performance through MF recovery. Given the crucial role of tactical performance in real-game situations [49], future research should emphasize exploring effective measures to restore MF and improve tactical performance.

Although Sun et al. [41] investigated the impact of MF on decision-making performance, including both offensive and defensive aspects, they did not assess specific motor skills. Most current studies focus on recovering MF to enhance offensive performance [12, 39, 40, 43], whereas there is a significant lack of intervention studies on improving defensive skills through MF recovery. Future research should delve deeper into the effects of MF on defensive skills, and performance [41].

Despite the search strategy of this review covering all 24 Olympic ball sports, the studies meeting the inclusion criteria were limited to basketball, soccer, and golf. This indicates a significant gap in intervention research on MF recovery in other ball sports. Future studies need to expand to more types of ball sports to comprehensively understand the demands and challenges of MF management across different sports.

### Practical application of MF recovery strategies

Existing studies on MF recovery strategies are mostly conducted in controlled laboratory or simulated environments [39, 40, 41, 43]. While Galanis et al. [12] used self-talk during tasks to enhance athletes’ attention, theoretically applicable during competitions, real-game MF and stress might be more complex and enduring. Although these interventions show significant effects in laboratory settings, their implementation in actual games faces various challenges such as time constraints and equipment availability. For example, Moreira et al. [43] demonstrated the beneficial effects of transcranial direct current stimulation (tDCS) on alleviating MF in experimental outcomes, but the operationalization of this technique in real game scenarios has not yet been validated. While these studies provide valuable insights, further research and validation are needed for their application and effectiveness in real-game environments.

Current research primarily focuses on the immediate impact of short-term interventions on MF, such as listening to relaxing music [39], mindfulness training [40], or natural visual stimuli [41]. These interventions show positive effects in reducing MF and enhancing sports performance, but their long-term effects lack sufficient research support. Considering the long season cycles of most professional ball sports [50], future research should focus on the sustained effects of long-term interventions throughout the season and potential adaptation issues athletes may face during prolonged recovery processes.

## Limitations

Although the keywords used in this study have endeavored to cover as many ball sports as possible, it is clearly not possible to cover all ball sports in the world. Only published articles in English were included in this review. Therefore, findings may have been affected by publication and language bias. PICO’s inclusion methodology may have resulted in qualitative studies not being included, which may have resulted in the loss of subjective experiences and experiences of recovery from MF in ballplayers.

## Conclusions

This study reveals that interventions such as listening to relaxing music, mindfulness training, self-talk, and exposure to natural visual stimuli are effective strategies to reduce MF and improve ball sports performance. However, these studies primarily focus on technical and cognitive performance in offensive phases. Therefore, future research should explore MF recovery strategies to enhance defensive skills and tactical performance. Most studies were conducted in controlled environments, showing encouraging results, but their practical application in real-game settings requires further investigation and validation. Additionally, considering the long seasons in professional ball sports, the long-term effects of MF interventions need more research.

## Supporting information

S1 Table Detailed search strategy. (PDF)

S2 Table PRISMA 2020 checklist. (PDF)

S3 Table Numbered table of all studies. (PDF)
