# Supplementary material for: Viable strategies for enhancing performance in ball sports by mitigating mental fatigue: A systematic review
Source: PLoS One. 2024 Nov 8;19(11):e0313105. doi: 10.1371/journal.pone.0313105 (PMC11548715; doi:10.1371/journal.pone.0313105)
Supplement: S1 Table — (PDF) [file pone.0313105.s001.pdf]

S1 Table. Detailed search strategy.

Database	Strategy	Results
Web of Science (1995–Jan 2024)	"mental fatigue" OR "cognitive fatigue" OR "mental effort" OR "cognitive effort" OR "mental exertion" OR "ego depletion" (All Fields) AND "performance" OR "decision making" OR "skill" OR "technique" (All Fields) AND "3x3 Basketball" OR "Badminton" OR "Baseball" OR "Softball" OR "Basketball" OR "Beach Handball" OR "Beach Volleyball" OR "Cricket" OR "Flag Football" OR "Football" OR "Futsal" OR "Golf" OR "Handball" OR "Hockey" OR "Ice Hockey" OR "Lacrosse" OR "Rugby Sevens" OR "Squash" OR "Table Tennis" OR "Tennis" OR "Volleyball" OR "Water Polo" (All Fields)	169
PubMed (1993–Jan 2024)	("mental fatigue"[All Fields] OR "cognitive fatigue"[All Fields] OR "mental effort"[All Fields] OR "cognitive effort"[All Fields] OR "mental exertion"[All Fields] OR "ego depletion"[All Fields]) AND ("performance"[All Fields] OR "decision making"[All Fields] OR "skill"[All Fields] OR "technique"[All Fields]) AND ("3x3 Basketball"[All Fields] OR "Badminton"[All Fields] OR "Baseball"[All Fields] OR "Softball"[All Fields] OR "Basketball"[All Fields] OR "Beach Handball"[All Fields] OR "Beach Volleyball"[All Fields] OR "Cricket"[All Fields] OR "Flag Football"[All Fields] OR "Football"[All Fields] OR "Futsal"[All Fields] OR "Golf"[All Fields] OR "Handball"[All Fields] OR "Hockey"[All Fields] OR "Ice Hockey"[All Fields] OR "Lacrosse"[All Fields] OR "Rugby Sevens"[All Fields] OR "Squash"[All Fields] OR "Table Tennis"[All Fields] OR "Tennis"[All Fields] OR "Volleyball"[All Fields] OR "Water Polo"[All Fields])	90
SPORTDiscus (1985–Jan 2024)	TX ("mental fatigue" OR "cognitive fatigue" OR "mental effort" OR "cognitive effort" OR "mental exertion" OR "ego depletion") AND TX ("performance" OR "decision making" OR "skill" OR "technique") AND AB ("3x3 Basketball" OR "Badminton" OR "Baseball" OR "Softball" OR "Basketball" OR "Beach Handball" OR "Beach Volleyball" OR "Cricket" OR "Flag Football" OR "Football" OR "Futsal" OR "Golf" OR "Handball" OR "Hockey" OR "Ice Hockey" OR "Lacrosse" OR "Rugby Sevens" OR "Squash" OR "Table Tennis" OR "Tennis" OR "Volleyball" OR "Water Polo")	396
SCOUPS (1982–Jan 2024)	(ALL ("mental fatigue" OR "cognitive fatigue" OR "mental effort" OR "cognitive effort" OR "mental exertion" OR "ego depletion") AND ALL ("performance" OR "decision making" OR "skill" OR "technique") AND TITLE-ABS-KEY ("3x3 Basketball" OR "Badminton" OR "Baseball" OR "Softball" OR "Basketball" OR "Beach Handball" OR "Beach Volleyball" OR "Cricket" OR "Flag Football" OR "Football" OR "Futsal" OR "Golf" OR "Handball" OR "Hockey" OR "Ice Hockey" OR "Lacrosse" OR "Rugby Sevens" OR "Squash" OR "Table Tennis" OR "Tennis" OR "Volleyball" OR "Water Polo"))	643
