# Supplementary material for: Viable strategies for enhancing performance in ball sports by mitigating mental fatigue: A systematic review
Source: PLoS One. 2024 Nov 8;19(11):e0313105. doi: 10.1371/journal.pone.0313105 (PMC11548715; doi:10.1371/journal.pone.0313105)
Supplement: S3 Table — (PDF) [file pone.0313105.s003.pdf]

S3 Table. Numbered table of all studies.

No.	Author(s)	Year	Title	Journal	Included in Analysis	Reason for Exclusion
1	W. Abbott; T. E. Brownlee; R. J. Naughton; T. Clifford; R. Page; L. D. Harper	2020	Changes in perceptions of mental fatigue during a season in professional under-23 English Premier League soccer players	RESEARCH IN SPORTS MEDICINE	No	Duplicate records
2	W. Abbott; T. E. Brownlee; R. J. Naughton; T. Clifford; R. Page; L. D. Harper	2020	Changes in perceptions of mental fatigue during a season in professional under-23 English Premier League soccer players	Research in Sports Medicine	No	Duplicate records
3	W. Abbott; C. Thomas; T. Clifford	2023	Effect of Playing Status and Fixture Congestion on Training Load, Mental Fatigue, and Recovery Status in Premier League Academy Goalkeepers	JOURNAL OF STRENGTH AND CONDITIONING RESEARCH	No	Duplicate records
4	H. S. Ahmed; S. M. Marcora; D. Dixon; G. Davison	2020	The effect of a competitive futsal match on psychomotor vigilance in referees	International Journal of Sports Physiology and Performance	No	Duplicate records
5	F. Alarcón; N. Ureña; D. Cárdenas	2017	Mental fatigue impairs the basketball free-throw performance	Revista de Psicologia del Deporte	No	Duplicate records
6	D. B. Alder; D. P. Broadbent; J. Stead; J. Poolton	2019	The impact of physiological load on anticipation skills in badminton: From testing to training	J Sports Sci	No	Duplicate records
7	D. B. Alder; D. P. Broadbent; J. Stead; J. Poolton	2019	The impact of physiological load on anticipation skills in badminton: From testing to training	JOURNAL OF SPORTS SCIENCES	No	Duplicate records
8	D. B. Alder; D. P. Broadbent; J. Stead; J. Poolton	2019	The impact of physiological load on anticipation skills in badminton: From testing to training	Journal of Sports Sciences	No	Duplicate records
9	D. B. Alder; P. R. Ford; J. Causer; A. M. Williams	2018	The effect of anxiety on anticipation, allocation of attentional resources, and visual search behaviours	HUMAN MOVEMENT SCIENCE	No	Duplicate records
10	D. B. Alder; P. R. Ford; J. Causer; A. M. Williams	2018	The effect of anxiety on anticipation, allocation of attentional resources, and visual search behaviours	Human Movement Science	No	Duplicate records
11	I. M. Alkhawaldeh	2023	The Effect of Mental Fatigue on the Accuracy of the Direct Free Kick in Terms of Some Kinematic Variables for Football Players	Asian Journal of Sports Medicine	No	Duplicate records
12	I. M. Alkhawaldeh; M. Altarawneh	2023	Effect of Trait and State Anxiety on Overhead Defensive Clear Shot Skill Performance Regarding Some Kinematic Variables for Badminton Players	Asian Journal of Sports Medicine	No	Duplicate records
13	M. S. Allen; M. Jones; P. J. McCarthy; S. Sheehan-Mansfield; D. Sheffield	2013	Emotions correlate with perceived mental effort and concentration disruption in adult sport performers	Eur J Sport Sci	No	Duplicate records
14	M. S. Allen; M. Jones; P. J. McCarthy; S. Sheehan-Mansfield; D. Sheffield	2013	Emotions correlate with perceived mental effort and concentration disruption in adult sport performers	EUROPEAN JOURNAL OF SPORT SCIENCE	No	Duplicate records
15	M. S. Allen; M. Jones; P. J. McCarthy; S. Sheehan-Mansfield; D. Sheffield	2013	Emotions correlate with perceived mental effort and concentration disruption in adult sport performers	European Journal of Sport Science	No	Duplicate records
16	L. Angius; M. Merlini; J.	2022	Physical and Mental Fatigue Reduce	Int J Sports Physiol Perform	No	Duplicate records

	Hopker; M. Bianchi; F. Fois; F. Piras; P. Cugia; J. Russell; S. M. Marcora		Psychomotor Vigilance in Professional Football Players			
17	L. Angius; M. Merlini; J. Hopker; M. Bianchi; F. Fois; F. Piras; P. Cugia; J. Russell; S. M. Marcora	2022	Physical and Mental Fatigue Reduce Psychomotor Vigilance in Professional Football Players	INTERNATIONAL JOURNAL OF SPORTS PHYSIOLOGY AND PERFORMANCE	No	Duplicate records
18	L. Angius; M. Merlini; J. Hopker; M. Bianchi; F. Fois; F. Piras; P. Cugia; J. Russell; S. M. Marcora	2022	Physical and Mental Fatigue Reduce Psychomotor Vigilance in Professional Football Players	International Journal of Sports Physiology and Performance	No	Duplicate records
19	D. Aras; A. S. Onlu; T. Durmus; C. Cengiz; D. Guler; Y. Guler; A. Ugurlu; M. I. Aldhahi; M. Gnln	2023	A brief body scan mindfulness practice has no positive effect on the recovery of heart rate variability and cognitive tasks in female professional basketball players	FRONTIERS IN PSYCHOLOGY	No	Duplicate records
20	G. Armando De Moraes; V. Nagy Soares; J. G. Cren Chiminazzo	2022	TEMPORAL ANALYSIS OF GOALS SCORED IN FUTSAL: A COMPARISON OF TWO MODELS	Human Movement	No	Duplicate records
21	M. Arvinen-Barrow; W. V. Massey; B. Hemmings	2014	Role of sport medicine professionals in addressing psychosocial aspects of sport-injury rehabilitation: Professional athletes' views	Journal of Athletic Training	No	Duplicate records
22	O. O. Badin; M. R. Smith; D. Conte; A. J. Coutts	2016	Mental Fatigue: Impairment of Technical Performance in Small-Sided Soccer Games	INTERNATIONAL JOURNAL OF SPORTS PHYSIOLOGY AND PERFORMANCE	No	Duplicate records
23	O. O. Badin; M. R. Smith; D. Conte; A. J. Coutts	2016	Mental fatigue: Impairment of technical performance in small-sided soccer games	International Journal of Sports Physiology and Performance	No	Duplicate records
24	T. Ballard; A. Neal; S. Farrell; E. Lloyd; J. Lim; A. Heathcote	2022	A General Architecture for Modeling the Dynamics of Goal-Directed Motivation and Decision-Making	PSYCHOLOGICAL REVIEW	No	Duplicate records
25	J. C. M. Barte; A. Nieuwenhuys; S. A. E. Geurts; M. A. J. Kompier	2019	Motivation counteracts fatigue-induced performance decrements in soccer passing performance	Journal of Sports Sciences	No	Duplicate records
26	J. C. M. Barte; A. Nieuwenhuys; S. A. E. Geurts; M. A. J. Kompier	2020	Effects of fatigue on interception decisions in soccer	International Journal of Sport and Exercise Psychology	No	Duplicate records
27	H. Ben Chikha; B. Zoudji; A. Khacharem	2023	An eye-tracking study of how coach's nonverbal communication affects memorization of basketball tactical scenes	International Journal of Sports Science and Coaching	No	Duplicate records
28	H. Ben Chikha; B. Zoudji; A. Khacharem	2023	The role of coach’s gaze guidance on memorization of tactical movements in basketball: an eye tracking study	German Journal of Exercise and Sport Research	No	Duplicate records
29	H. Ben Chikha; B. Zoudji; A. Khacharem	2023	Using pointing gestures to convey tactical information: investigating the roles of expertise and complexity	PSYCHOLOGICAL RESEARCH-PSYCHOLOGISCHE FORSCHUNG	No	Duplicate records
30	H. Ben Chikha; B. Zoudji; A. Khacharem	2023	Using pointing gestures to convey tactical information: investigating the roles of expertise and complexity	Psychological Research	No	Duplicate records
31	K. Berestetska	2019	The Relationship Between Perceived Coaching Behaviors, Intrinsic Motivation, and Scholarship Status on NCAA Division I Tennis Players' Sport Commitment	Journal of Sport Behavior	No	Duplicate records
32	Z. Bilić; F. Sinković; P. Barbaros; D. Novak; E. Zemkova	2023	Exercise-Induced Fatigue Impairs Change of Direction Performance and Serve Precision among Young Male Tennis Players	Sports	No	Duplicate records
33	F. Bloise Mundstock; F.	2021	Goal difference relationship between the	Journal of Physical Education	No	Duplicate records

	H. Da Silva Maia; C. C. Fonseca Bicalho		national leagues of Brazil, Germany and England from the perspective of the prospect theory	& Sport		
34	R. Boat; C. Sunderland; S. B. Cooper	2021	Detrimental effects of prior self-control exertion on subsequent sporting skill performance	SCANDINAVIAN JOURNAL OF MEDICINE & SCIENCE IN SPORTS	No	Duplicate records
35	R. Boat; C. Sunderland; S. B. Cooper	2021	Detrimental effects of prior self-control exertion on subsequent sporting skill performance	Scandinavian Journal of Medicine and Science in Sports	No	Duplicate records
36	R. Boat; C. Sunderland; S. B. Cooper	2021	Detrimental effects of prior self-control exertion on subsequent sporting skill performance	Scand J Med Sci Sports	No	Duplicate records
37	N. Bonney; K. Ball; J. Berry; P. Larkin	2020	Effects of manipulating player numbers on technical and physical performances participating in an Australian football small-sided game	Journal of Sports Sciences	No	Duplicate records
38	S. Brini; A. B. Abderrahman; C. C. T. Clark; S. Zouita; A. C. Hackney; K. Govindasamy; U. Granacher; H. Zouhal	2021	Sex-specific effects of small-sided games in basketball on psychometric and physiological markers during Ramadan intermittent fasting: a pilot study	BMC Sports Science, Medicine & Rehabilitation	No	Duplicate records
39	S. Brini; A. B. Abderrahman; C. C. T. Clark; S. Zouita; A. C. Hackney; K. Govindasamy; U. Granacher; H. Zouhal	2021	Sex-specific effects of small-sided games in basketball on psychometric and physiological markers during Ramadan intermittent fasting: a pilot study	BMC Sports Science, Medicine and Rehabilitation	No	Duplicate records
40	S. Brini; A. Ben Abderrahman; C. C. T. Clark; S. Zouita; A. C. Hackney; K. Govindasamy; U. Granacher; H. Zouhal	2021	Sex-specific effects of small-sided games in basketball on psychometric and physiological markers during Ramadan intermittent fasting: a pilot study	BMC SPORTS SCIENCE MEDICINE AND REHABILITATION	No	Duplicate records
41	D. P. Broadbent; J. Causer; A. Mark Williams; P. R. Ford	2017	The role of error processing in the contextual interference effect during the training of perceptual-cognitive skills	Journal of Experimental Psychology: Human Perception and Performance	No	Duplicate records
42	D. P. Broadbent; J. Causer; A. M. Williams; P. R. Ford	2017	The Role of Error Processing in the Contextual Interference Effect During the Training of Perceptual-Cognitive Skills	JOURNAL OF EXPERIMENTAL PSYCHOLOGY-HUMAN PERCEPTION AND PERFORMANCE	No	Duplicate records
43	P. Camacho; D. A. Cruz; I. Madinabeitia; F. J. Giménez; D. Cárdenas	2021	Time Constraint Increases Mental Load and Influences in the Performance in Small-Sided Games in Basketball	Research Quarterly for Exercise and Sport	No	Duplicate records
44	A. Camacho-Cardenosa; M. Camacho-Cardenosa; J. Brazo-Sayavera	2019	ENDURANCE ASSESSMENT IN HANDBALL: A SYSTEMATIC REVIEW. / EVALUACIÓN DE LA RESISTENCIA EN BALONMANO: UNA REVISIÓN SISTEMÁTICA	European Journal of Human Movement	No	Duplicate records
45	M. J. Campbell; A. P. Moran; N. Bargary; S. Surmon; L. Bressan; I. C. Kenny	2019	Pupillometry during golf putting: A new window on the cognitive mechanisms underlying quiet eye	Sport, Exercise, and Performance Psychology	No	Duplicate records
46	S. Cao; S. K. Geok; S. Roslan; S. Qian; H. Sun; S. K. Lam; J. Liu	2022	Mindfulness-Based Interventions for the Recovery of Mental Fatigue: A Systematic Review	International Journal of Environmental Research and Public Health	No	Duplicate records
47	S. Cao; S. K. Geok; S.	2021	Mental Fatigue and Basketball Performance:	Front Psychol	No	Duplicate records

	Roslan; H. Sun; S. K. Lam; S. Qian		A Systematic Review			
48	S. D. Cao; S. K. Geok; S. Roslan; S. W. Qian; H. Sun; S. K. Lam; J. Liu	2022	Mindfulness-Based Interventions for the Recovery of Mental Fatigue: A Systematic Review	INTERNATIONAL JOURNAL OF ENVIRONMENTAL RESEARCH AND PUBLIC HEALTH	No	Duplicate records
49	S. D. Cao; S. K. Geok; S. Roslan; H. Sun; S. K. Lam; S. W. Qian	2022	Mental Fatigue and Basketball Performance: A Systematic Review	FRONTIERS IN PSYCHOLOGY	No	Duplicate records
50	E. Carnegie; D. Marchant; S. Towers; P. Ellison	2020	Beyond visual fixations and gaze behaviour. Using pupillometry to examine the mechanisms in the planning and motor performance of a golf putt	HUMAN MOVEMENT SCIENCE	No	Duplicate records
51	E. Carnegie; D. Marchant; S. Towers; P. Ellison	2020	Beyond visual fixations and gaze behaviour. Using pupillometry to examine the mechanisms in the planning and motor performance of a golf putt	Human Movement Science	No	Duplicate records
52	F. Carson; C. McCormack; P. McGovern; S. Ralston; J. Walsh	2021	Coach like a woman: Learnings from a pilot coach education program	Women in Sport and Physical Activity Journal	No	Duplicate records
53	H. J. Carson; D. Collins; J. Richards	2014	Intra-individual movement variability during skill transitions: a useful marker?	Eur J Sport Sci	No	Duplicate records
54	H. J. Carson; D. Collins; J. Richards	2014	Intra-individual movement variability during skill transitions: A useful marker?	EUROPEAN JOURNAL OF SPORT SCIENCE	No	Duplicate records
55	H. J. Carson; D. Collins; J. Richards	2014	Intra-individual movement variability during skill transitions: A useful marker?	European Journal of Sport Science	No	Duplicate records
56	H. J. Carson; D. Collins; J. Richards	2016	Initiating technical refinements in high-level golfers: Evidence for contradictory procedures	European Journal of Sport Science	No	Duplicate records
57	F. Casanova; P. T. Esteves; M. B. Padilha; J. Ribeiro; A. M. Williams; J. Garganta	2022	The Effects of Physiological Demands on Visual Search Behaviours During 2 vs. 1 + GK Game Situations in Football: An in-situ Approach	Frontiers in Psychology	No	Duplicate records
58	F. Casanova; P. T. Esteves; M. B. Padilha; J. Ribeiro; A. M. Williams; J. Garganta	2022	The Effects of Physiological Demands on Visual Search Behaviours During 2 vs. 1+GK Game Situations in Football: An <i>in-situ</i> Approach	FRONTIERS IN PSYCHOLOGY	No	Duplicate records
59	G. Ciocca; A. Tessitore; M. Mandorino; H. Tschan	2022	A Video-Based Tactical Task Does Not Elicit Mental Fatigue and Does Not Impair Soccer Performance in a Subsequent Small-Sided Game	SPORTS	No	Duplicate records
60	G. Ciocca; A. Tessitore; M. Mandorino; H. Tschan	2022	A Video-Based Tactical Task Does Not Elicit Mental Fatigue and Does Not Impair Soccer Performance in a Subsequent Small-Sided Game	Sports	No	Duplicate records
61	F. M. Clemente; R. Ramirez-Campillo; D. Castillo; J. Raya-González; A. F. Silva; J. Afonso; H. Sarmento; T. Rosemann; B. Knechtle	2021	Effects of Mental Fatigue in Total Running Distance and Tactical Behavior During Small-Sided Games: A Systematic Review With a Meta-Analysis in Youth and Young Adult's Soccer Players	FRONTIERS IN PSYCHOLOGY	No	Duplicate records
62	F. M. Clemente; R. Ramirez-Campillo; D. Castillo; J. Raya-González; A. F. Silva; J. Afonso; H.	2021	Effects of Mental Fatigue in Total Running Distance and Tactical Behavior During Small-Sided Games: A Systematic Review With a Meta-Analysis in Youth and Young Adult's Soccer Players	Frontiers in Psychology	No	Duplicate records

	Sarmento; T. Rosemann; B. Knechtle					
63	A. J. Cocks; R. C. Jackson; D. T. Bishop; A. M. Williams	2016	Anxiety, anticipation and contextual information: A test of attentional control theory	COGNITION & EMOTION	No	Duplicate records
64	A. J. Cocks; R. C. Jackson; D. T. Bishop; A. M. Williams	2016	Anxiety, anticipation and contextual information: A test of attentional control theory	Cognition and Emotion	No	Duplicate records
65	D. R. Coimbra; G. G. Bevilacqua; F. S. Pereira; A. Andrade	2021	Effect of Mindfulness Training on Fatigue and Recovery in Elite Volleyball Athletes: A Randomized Controlled Follow-Up Study	JOURNAL OF SPORTS SCIENCE AND MEDICINE	No	Duplicate records
66	D. R. Coimbra; G. G. Bevilacqua; F. S. Pereira; A. Andrade	2021	Effect of mindfulness training on fatigue and recovery in elite volleyball athletes: A randomized controlled follow-up study	Journal of Sports Science and Medicine	No	Duplicate records
67	D. Conte; M. R. Smith; F. Santolamazza; T. G. Favero; A. Tessitore; A. Coutts	2019	Reliability, usefulness and construct validity of the Combined Basketball Skill Test (CBST)	Journal of Sports Sciences	No	Duplicate records
68	A. Cooke; M. Kavussanu; D. McIntyre; I. D. Boardley; C. Ring	2011	Effects of competitive pressure on expert performance: Underlying psychological, physiological, and kinematic mechanisms	Psychophysiology	No	Duplicate records
69	E. K. Coughlan; A. M. Williams; P. R. Ford	2019	Lessons From the Experts: The Effect of a Cognitive Processing Intervention During Deliberate Practice of a Complex Task	J Sport Exerc Psychol	No	Duplicate records
70	E. K. Coughlan; A. M. Williams; P. R. Ford	2019	Lessons From the Experts: The Effect of a Cognitive Processing Intervention During Deliberate Practice of a Complex Task	JOURNAL OF SPORT & EXERCISE PSYCHOLOGY	No	Duplicate records
71	E. K. Coughlan; A. M. Williams; P. R. Ford	2019	Lessons From the Experts: The Effect of a Cognitive Processing Intervention During Deliberate Practice of a Complex Task	Journal of Sport & Exercise Psychology	No	Duplicate records
72	D. Coutinho; B. Gonçalves; B. Travassos; H. Folgado; B. Figueira; J. Sampaio	2020	Different Marks in the Pitch Constraint Youth Players' Performances During Football Small-sided Games	Research Quarterly for Exercise and Sport	No	Duplicate records
73	A. J. Coutts	2016	Fatigue in football: it's not a brainless task!	Journal of Sports Sciences	No	Duplicate records
74	J. O. C. Coyne; A. J. Coutts; R. U. Newton; G. G. Haff	2021	The Influence of Mental Fatigue on Sessional Ratings of Perceived Exertion in Elite Open and Closed Skill Sports Athletes	JOURNAL OF STRENGTH AND CONDITIONING RESEARCH	No	Duplicate records
75	J. O. C. Coyne; A. J. Coutts; R. U. Newton; G. G. Haff	2021	The Influence of Mental Fatigue on Sessional Ratings of Perceived Exertion in Elite Open and Closed Skill Sports Athletes	Journal of Strength and Conditioning Research	No	Duplicate records
76	J. O. C. Coyne; G. G. Haff; A. J. Coutts; R. U. Newton; S. Nimphius	2018	The Current State of Subjective Training Load Monitoring-a Practical Perspective and Call to Action	SPORTS MEDICINE-OPEN	No	Duplicate records
77	D. C. da Silva; J. Afonso; D. Augusto; G. H. Petiot; C. C. Martins; F. Vasconcellos	2023	Influence of pre-induced mental fatigue on tactical behaviour and performance among young elite football players	INTERNATIONAL JOURNAL OF SPORT AND EXERCISE PSYCHOLOGY	No	Duplicate records
78	D. C. da Silva; D. M. Carnevale; D. A. N. Santos; C. D. Andrade; C. C. Martins; F. Vasconcellos	2024	Mental fatigue in football: behavioural responses of players with high and low tactical performance	RETOS-NUEVAS TENDENCIAS EN EDUCACION FISICA DEPORTE Y RECREACION	No	Duplicate records
79	B. D. Daub; B. D. McLean; A. D. Heishman; A. J. Coutts	2023	The reliability and usefulness of a novel basketball standardized shooting task	INTERNATIONAL JOURNAL OF SPORTS SCIENCE & COACHING	No	Duplicate records
80	B. D. Daub; B. D. McLean; A. D. Heishman;	2023	The reliability and usefulness of a novel basketball standardized shooting task	International Journal of Sports Science and Coaching	No	Duplicate records

	A. J. Coutts					
81	B. D. Daub; B. D. McLean; A. D. Heishman; K. M. Peak; A. J. Coutts	2023	Impacts of mental fatigue and sport specific film sessions on basketball shooting tasks	EUROPEAN JOURNAL OF SPORT SCIENCE	No	Duplicate records
82	B. D. Daub; B. D. McLean; A. D. Heishman; K. M. Peak; A. J. Coutts	2023	Impacts of mental fatigue and sport specific film sessions on basketball shooting tasks	European Journal of Sport Science	No	Duplicate records
83	D. Davidow; M. Smith; T. Ross; G. L. James; L. Paul; M. Lambert; B. Jones; S. Hendricks	2023	Mental Fatigue Impairs Tackling Technique in Amateur Rugby Union Players	International Journal of Sports Physiology and Performance	No	Duplicate records
84	D. Davidow; M. Smith; T. Ross; G. Laura James; L. Paul; M. Lambert; B. Jones; S. Hendricks	2023	Mental Fatigue Impairs Tackling Technique in Amateur Rugby Union Players	Int J Sports Physiol Perform	No	Duplicate records
85	K. Davies; B. Staples; C. Morris	2020	Accommodate and adapt - Coaching in COVID-19 environment	Coaching & Sport Science Review	No	Duplicate records
86	V. H. de Freitas; S. D. P. Ramos; A. Leicht; T. Alves; F. Rabelo; M. G. Bara-Filho; F. A. Guarnier; F. Y. Nakamura	2017	Validation of the futsal-specific intermittent shuttle protocol for the simulation of the physical demands of futsal match-play	International Journal of Performance Analysis in Sport	No	Duplicate records
87	G. de Sousa Pinheiro; H. Soares Bernardino; I. Teoldo Costa; V. Teoldo Costa	2022	Differences in discriminative reaction time between elite youth football players: a comparison between age-categories. / Diferencias en el tiempo de reacción discriminativo entre jugadores de fútbol juvenil de élite: una comparación entre categorías de edad	Retos: Nuevas Perspectivas de Educación Física, Deporte y Recreación	No	Duplicate records
88	A. Delextrat; J. Calleja-González; A. Hippocrate; N. Clarke	2013	Effects of sports massage and intermittent cold-water immersion on recovery from matches by basketball players	Journal of Sports Sciences	No	Duplicate records
89	R. A. Denardi; F. A. R. Clavijo; T. A. C. De Oliveira; S. L. Da Silva; B. Travassos; U. C. Corrêa	2017	The influence of defender's positional gap on the aces in the sport of volleyball	Journal of Human Sport and Exercise	No	Duplicate records
90	J. Díaz-García; V. J. Clemente-Suárez; J. P. Fuentes-García; S. Villafaina	2023	Combining HIIT Plus Cognitive Task Increased Mental Fatigue but Not Physical Workload in Tennis Players	Applied Sciences (Switzerland)	No	Duplicate records
91	J. Díaz-García; J. C. Ponce-Bordón; A. Moreno-Gil; A. Rubio-Morales; M. López-Gajardo; T. García-Calvo	2023	Influence of Scoring Systems on Mental Fatigue, Physical Demands, and Tactical Behavior during Soccer Large-Sided Games	Int J Environ Res Public Health	No	Duplicate records
92	S. H. Doeven; M. S. Brink; B. C. H. Huijgen; J. de Jong; K. A. P. M. Lemmink	2021	Managing load to optimize well-being and recovery during short-term match congestion in elite basketball	International Journal of Sports Physiology and Performance	No	Duplicate records
93	R. I. Doewes; G. Elumalai; S. H. Azmi	2022	Development of long pass test instruments in football	Journal of Physical Education and Sport	No	Duplicate records
94	D. C. Dorris; D. A. Power; E. Kenefick	2012	Investigating the effects of ego depletion on physical exercise routines of athletes	Psychology of Sport and Exercise	No	Duplicate records
95	R. Duffield; A. Coutts; A. McCall; D. Burgess	2013	Pre-cooling for football training and competition in hot and humid conditions	European Journal of Sport Science	No	Duplicate records
96	M. J. Duncan; S. Taylor; M. Lyons	2012	The Effect of Caffeine Ingestion on Field Hockey Skill Performance Following	RESEARCH IN SPORTS MEDICINE	No	Duplicate records

			Physical Fatigue			
97	M. J. Duncan; S. Taylor; M. Lyons	2012	The effect of caffeine ingestion on field hockey skill performance following physical fatigue	Research in Sports Medicine	No	Duplicate records
98	M. J. Duncan; S. Taylor; M. Lyons	2012	The effect of caffeine ingestion on field hockey skill performance following physical fatigue	Res Sports Med	No	Duplicate records
99	C. Englert; A. Bertrams	2012	Anxiety, ego depletion, and sports performance	J Sport Exerc Psychol	No	Duplicate records
100	C. Englert; A. Bertrams	2012	Anxiety, Ego Depletion, and Sports Performance	JOURNAL OF SPORT & EXERCISE PSYCHOLOGY	No	Duplicate records
101	C. Englert; A. Bertrams	2012	Anxiety, ego depletion, and sports performance	Journal of Sport and Exercise Psychology	No	Duplicate records
102	C. Englert; A. Bertrams	2015	Autonomy as a protective factor against the detrimental effects of ego depletion on tennis serve accuracy under pressure	INTERNATIONAL JOURNAL OF SPORT AND EXERCISE PSYCHOLOGY	No	Duplicate records
103	C. Englert; A. Bertrams	2015	Autonomy as a protective factor against the detrimental effects of ego depletion on tennis serve accuracy under pressure	International Journal of Sport and Exercise Psychology	No	Duplicate records
104	C. Englert; A. Bertrams	2016	Active relaxation counteracts the effects of ego depletion on performance under evaluative pressure in a state of ego depletion	Sportwissenschaft	No	Duplicate records
105	C. Englert; A. Bertrams; P. Purley; R. R. D. Oudejans	2015	Is ego depletion associated with increased distractibility? Results from a basketball free throw task	PSYCHOLOGY OF SPORT AND EXERCISE	No	Duplicate records
106	H. Faro; D. Cavalcante Silva; B. T. Barbosa; Y. P. D. Costa; C. G. Freitas-Junior; D. de Lima-Junior; J. Faubert; L. D. S. Fortes	2023	Young Basketball Players’ Multiple Object Tracking Skills Were Unaffected by Stroop-Induced Mental Fatigue	Perceptual and Motor Skills	No	Duplicate records
107	H. Faro; D. Cavalcante Silva; B. T. Barbosa; Y. P. D. Costa; C. G. Freitas-Junior; D. de Lima-Junior; J. Faubert; L. S. Fortes	2023	Young Basketball Players' Multiple Object Tracking Skills Were Unaffected by Stroop-Induced Mental Fatigue	Percept Mot Skills	No	Duplicate records
108	H. Faro; L. D. Fortes; D. de Lima-Junior; B. T. Barbosa; M. E. C. Ferreira; S. S. Almeida	2022	Sport-based video game causes mental fatigue and impairs visuomotor skill in male basketball players	INTERNATIONAL JOURNAL OF SPORT AND EXERCISE PSYCHOLOGY	No	Duplicate records
109	H. Faro; D. C. Silva; B. T. Barbosa; Y. P. da Costa; C. G. Freitas-Junior; D. de Lima-Junior; J. Faubert; L. D. Fortes	2023	Young Basketball Players' Multiple Object Tracking Skills Were Unaffected by Stroop-Induced Mental Fatigue	PERCEPTUAL AND MOTOR SKILLS	No	Duplicate records
110	R. FernÁNdez; F. Zurita; M. A. R. Cepero; V. A. Molina; J. M. Vilches; J. Ambris	2015	Influence of the physical and psychological variables on physical injuries in football	Journal of Human Sport & Exercise	No	Duplicate records
111	X. E. Fernández; M. Priego-Ojeda; A. R. Morente; C. A. Mora	2022	Relationship between emotional intelligence, burnout and health perception in a sample of football Spanish referees	Retos	No	Duplicate records
112	L. Filipas; D. Ferioli; G. Banfi; A. La Torre; J. A. Vitale	2021	Single and Combined Effect of Acute Sleep Restriction and Mental Fatigue on Basketball Free-Throw Performance	Int J Sports Physiol Perform	No	Duplicate records

113	L. Filipas; D. Ferioli; G. Banfi; A. La Torre; J. A. Vitale	2021	Single and Combined Effect of Acute Sleep Restriction and Mental Fatigue on Basketball Free-Throw Performance	INTERNATIONAL JOURNAL OF SPORTS PHYSIOLOGY AND PERFORMANCE	No	Duplicate records
114	L. Filipas; D. Ferioli; G. Banfi; A. La Torre; J. A. Vitale	2021	Single and Combined Effect of Acute Sleep Restriction and Mental Fatigue on Basketball Free-Throw Performance	International Journal of Sports Physiology & Performance	No	Duplicate records
115	L. Filipas; C. Rossi; R. Codella; M. Bonato	2023	Mental Fatigue Impairs Second Serve Accuracy in Tennis Players	RESEARCH QUARTERLY FOR EXERCISE AND SPORT	No	Duplicate records
116	L. Filipas; C. Rossi; R. Codella; M. Bonato	2023	Mental Fatigue Impairs Second Serve Accuracy in Tennis Players	Research Quarterly for Exercise and Sport	No	Duplicate records
117	Z. A. Fitrian; A. S. Graha; A. Nasrulloh; A. Munir; M. Asmara; N. Y. Irsyad	2023	The effect of circuit training, fartlek, and small-sided games on maximum oxygen consumption capacity building in futsal players. / Занзабил Адва Фитриан, Али Сатья Граха, Ахмад Насрулло, Али Мунир, Мирза Асмара, Науфаллатхуф Якуттул Ирсяд. Влияние круговых тренировок, фартлека и игр с малым составом на развитие способности к максимальному потреблению кислорода у футзалистов	Health Sport Rehabilitation	No	Duplicate records
118	B. D. Fletcher; C. Twist; J. D. Haigh; C. Brewer; J. P. Morton; G. L. Close	2016	Season-long increases in perceived muscle soreness in professional rugby league players: role of player position, match characteristics and playing surface	Journal of Sports Sciences	No	Duplicate records
119	H. Folgado; R. Duarte; P. Marques; J. Sampaio	2015	The effects of congested fixtures period on tactical and physical performance in elite football	Journal of Sports Sciences	No	Duplicate records
120	L. S. Fortes; G. P. Berriel; H. Faro; C. G. Freitas; L. A. Peyré-Tartaruga	2022	Can Prolongate Use of Social Media Immediately Before Training Worsen High Level Male Volleyball Players' Visuomotor Skills?	PERCEPTUAL AND MOTOR SKILLS	No	Duplicate records
121	L. S. Fortes; G. P. Berriel; H. Faro; C. G. Freitas-Júnior; L. A. Peyré-Tartaruga	2022	Can Prolongate Use of Social Media Immediately Before Training Worsen High Level Male Volleyball Players' Visuomotor Skills?	Percept Mot Skills	No	Duplicate records
122	L. S. Fortes; G. P. Berriel; H. Faro; C. G. Freitas-Júnior; L. A. Peyré-Tartaruga	2022	Can Prolongate Use of Social Media Immediately Before Training Worsen High Level Male Volleyball Players’ Visuomotor Skills?	Perceptual and Motor Skills	No	Duplicate records
123	L. S. Fortes; M. E. C. Ferreira; H. Faro; E. M. Penna; S. S. Almeida	2022	Brain Stimulation Over the Motion-Sensitive Midtemporal Area Reduces Deleterious Effects of Mental Fatigue on Perceptual-Cognitive Skills in Basketball Players	JOURNAL OF SPORT & EXERCISE PSYCHOLOGY	No	Duplicate records
124	L. S. Fortes; M. E. C. Ferreira; H. Faro; E. M. Penna; S. S. Almeida	2022	Brain Stimulation Over the Motion-Sensitive Midtemporal Area Reduces Deleterious Effects of Mental Fatigue on Perceptual-Cognitive Skills in Basketball Players	Journal of sport & exercise psychology	No	Duplicate records
125	L. S. Fortes; M. E. C. Ferreira; H. Faro; E. M. Penna; S. S. Almeida	2022	Brain Stimulation Over the Motion-Sensitive Midtemporal Area Reduces Deleterious Effects of Mental Fatigue on Perceptual-Cognitive Skills in Basketball Players	J Sport Exerc Psychol	No	Duplicate records
126	L. S. Fortes; F. S. Fonseca;	2021	Effects of Mental Fatigue Induced by Social	PERCEPTUAL AND	No	Duplicate records

	F. Y. Nakamura; B. T. Barbosa; P. Gantois; D. de Lima; M. E. C. Ferreira		Media Use on Volleyball Decision-Making, Endurance, and Countermovement Jump Performance	MOTOR SKILLS		
127	L. S. Fortes; F. S. Fonseca; F. Y. Nakamura; B. T. Barbosa; P. Gantois; D. de Lima-Júnior; M. E. C. Ferreira	2021	Effects of Mental Fatigue Induced by Social Media Use on Volleyball Decision-Making, Endurance, and Countermovement Jump Performance	Percept Mot Skills	No	Duplicate records
128	L. S. Fortes; F. S. Fonseca; F. Y. Nakamura; B. T. Barbosa; P. Gantois; D. de Lima-Júnior; M. E. C. Ferreira	2021	Effects of Mental Fatigue Induced by Social Media Use on Volleyball Decision-Making, Endurance, and Countermovement Jump Performance	Perceptual and Motor Skills	No	Duplicate records
129	L. S. Fortes; D. Lima; B. T. Barbosa; H. K. C. Faro; M. E. C. Ferreira; S. S. Almeida	2022	Effect of mental fatigue on decision-making skill and visual search behaviour in basketball players: an experimental and randomised study	INTERNATIONAL JOURNAL OF SPORT AND EXERCISE PSYCHOLOGY	No	Duplicate records
130	J. L. Fox; C. J. O'Grady; A. T. Scanlan	2020	Game schedule congestion affects weekly workloads but not individual game demands in semi-professional basketball	Biology of Sport	No	Duplicate records
131	J. P. Fuentes-García; J. Díaz-García; M. Á. López-Gajardo; V. J. Clemente-Suarez	2021	Effects of combined hiit and stroop on strength manifestations, serve speed and accuracy in recreational tennis players	Sustainability (Switzerland)	No	Duplicate records
132	H. H. Fullagar; S. Skorski; R. Duffield; R. Julian; J. Bartlett; T. Meyer	2016	Impaired sleep and recovery after night matches in elite football players	J Sports Sci	No	Duplicate records
133	P. Furley; A. Bertrams; C. Englert; A. Delphia	2013	Ego depletion, attentional control, and decision making in sport	Psychology of Sport and Exercise	No	Duplicate records
134	E. Galanis; A. Hatzigeorgiadis; N. Comoutos; F. Charachousi; X. Sanchez	2018	From the lab to the field: Effects of self-talk on task performance under distracting conditions	Sport Psychologist	No	Duplicate records
135	E. Galanis; L. Nurkse; J. Kooijman; E. Papagiannis; A. Karathanasi; N. Comoutos; Y. Theodorakis; A. Hatzigeorgiadis	2022	Effects of a Strategic Self-Talk Intervention on Attention Functions and Performance in a Golf Task under Conditions of Ego Depletion	SUSTAINABILITY	No	Duplicate records
136	T. García-Calvo; I. González-Ponce; J. C. Ponce; D. Tomé-Lourido; Á. Vales-Vázquez	2019	Incidence of the tasks scoring system on the mental load in football training	Revista de Psicologia del Deporte	No	Duplicate records
137	P. Gaudreau; A. Nicholls; A. R. Levy	2010	The ups and downs of coping and sport achievement: An episodic process analysis of within-person associations	Journal of Sport and Exercise Psychology	No	Duplicate records
138	A. Ghavami; H. Samadi; A. Dana; S. Ghorbani	2022	Effects of observing real, animated and combined model on learning cognitive and motor levels of basketball jump shot in children	Biomedical Human Kinetics	No	Duplicate records
139	F. R. Goes; M. S. Brink; M. T. Elferink-Gemser; M. Kempe; K. A. P. M. Lemmink	2021	The tactics of successful attacks in professional association football: large-scale spatiotemporal analysis of dynamic subgroups using position tracking data	Journal of Sports Sciences	No	Duplicate records
140	Y. Gok; E. Suel; Y. Soylu	2023	Effects of different small-sided games on psychological responses and technical activities in young female basketball players	Acta Gymnica	No	Duplicate records
141	P. Goldman; B. Pandit; D.	2022	Effect of Real-Time Feedback on Power	International Journal of	No	Duplicate records

	Gomez; S. Lu; C. Mills; N. Kull; R. Ku; A. Aramie; A. Kim; A. Alexandru; J. Hu; E. V. Neufeld; B. A. Dolezal		Output Using a Novel Smart-Resisted Sled Push	Exercise Science		
142	N. Goldschmied; M. Raphaeli; S. Moothart; P. Furley	2022	Free throw shooting performance under pressure: a social psychology critical review of research	International Journal of Sport and Exercise Psychology	No	Duplicate records
143	N. Goldschmied; D. Vira	2019	The experience of ego threat in the public arena: A study of air ball shots performance in collegiate basketball	Journal of Applied Social Psychology	No	Duplicate records
144	R. Gomes; A. Coutts; L. Viveiros; M. Aoki	2011	Physiological demands of match-play in elite tennis: A case study	European Journal of Sport Science	No	Duplicate records
145	B. Gonçalves; D. Coutinho; B. Travassos; H. Folgado; P. Caixinha; J. Sampaio	2018	Speed synchronization, physical workload and match-to-match performance variation of elite football players	PLOS ONE	No	Duplicate records
146	B. Gonçalves; D. Coutinho; B. Travassos; H. Folgado; P. Caixinha; J. Sampaio	2018	Speed synchronization, physical workload and match-to-match performance variation of elite football players	PLoS ONE	No	Duplicate records
147	S. González-Víllora; A. Prieto-Ayuso; F. Cardoso; I. Teoldo	2022	The role of mental fatigue in soccer: a systematic review	International Journal of Sports Science and Coaching	No	Duplicate records
148	J. D. Graham; B. Zhang; D. M. Y. Brown; J. Cairney	2022	The Home Advantage in the National Basketball Association Conference Finals and Finals Series From 1979 to 2019: A Mediation Analysis of Offensive and Defensive Skills	Journal of Sport and Exercise Psychology	No	Duplicate records
149	A. Guillot; C. Collet	2005	Duration of mentally simulated movement: A review	Journal of Motor Behavior	No	Duplicate records
150	W. Guo; J. Ren; B. Y. Wang; Q. Zhu	2015	Effects of Relaxing Music on Mental Fatigue Induced by a Continuous Performance Task: Behavioral and ERPs Evidence	PLOS ONE	No	Duplicate records
151	J. Habay; M. Proost; J. De Wachter; J. Díaz-García; K. De Pauw; R. Meeusen; J. Van Cutsem; B. Roelands	2021	Mental Fatigue-Associated Decrease in Table Tennis Performance: Is There an Electrophysiological Signature?	INTERNATIONAL JOURNAL OF ENVIRONMENTAL RESEARCH AND PUBLIC HEALTH	No	Duplicate records
152	A. D. Hagstrom; K. A. Shorter	2018	Creatine kinase, neuromuscular fatigue, and the contact codes of football: A systematic review and meta-analysis of pre- and post-match differences	European Journal of Sport Science	No	Duplicate records
153	N. Haller; T. Ehlert; S. Schmidt; D. Ochmann; B. Sterzing; F. Grus; P. Simon	2019	Circulating, Cell-Free DNA for Monitoring Player Load in Professional Football	Int J Sports Physiol Perform	No	Duplicate records
154	N. Haller; T. Ehlert; S. Schmidt; D. Ochmann; B. Sterzing; F. Grus; P. Simon	2019	Circulating, Cell-Free DNA for Monitoring Player Load in Professional Football	INTERNATIONAL JOURNAL OF SPORTS PHYSIOLOGY AND PERFORMANCE	No	Duplicate records
155	N. Haller; T. Ehlert; S. Schmidt; D. Ochmann; B. Sterzing; F. Grus; P. Simon	2019	Circulating, cell-free DNA for monitoring player load in professional football	International Journal of Sports Physiology and Performance	No	Duplicate records
156	N. Haller; E. Hübler; T. Stöggl; P. Simon	2022	Evidence-Based Recovery in Soccer – Low-Effort Approaches for Practitioners	Journal of Human Kinetics	No	Duplicate records
157	A. Harrison; A.	2022	Concussion History and Heart Rate	Journal of Athletic Training	No	Duplicate records

	Lane-Cordova; M. F. La Fountaine; R. D. Moore		Variability During Bouts of Acute Stress	(Allen Press)		
158	S. J. Haslerig	2017	Graduate(d) student athletes in Division I football: Redefining archetypes and disrupting stereotypes or invisible?	Sociology of Sport Journal	No	Duplicate records
159	R. Hayman; R. Polman; E. Borkoles; J. Taylor	2013	The influence of a deliberate practice intervention on the putting performance and subsequent practice behaviours of aspiring elite adolescent golfers	Talent Development and Excellence	No	Duplicate records
160	R. Hayman; R. Polman; J. Taylor; B. Hemmings; E. Borkoles	2011	Development of Elite Adolescent Golfers	Talent Development & Excellence	No	Duplicate records
161	T. J. Hepler; M. Andre	2022	Does stress type and level affect gut decisions in sport?	International Journal of Sport & Exercise Psychology	No	Duplicate records
162	T. J. Hepler; A. J. Kovacs	2017	Influence of acute stress on decision outcomes and heuristics	Journal of Sports Medicine and Physical Fitness	No	Duplicate records
163	D. L. Herbert	2010	New NFL, Professional Association and State Actions on Concussions	Sports Medicine Standards & Malpractice Reporter	No	Duplicate records
164	G. R. Hockey; F. Earle	2006	Control over the scheduling of simulated office work reduces the impact of workload on mental fatigue and task performance	J Exp Psychol Appl	No	Duplicate records
165	L. W. Hogarth; B. J. Burkett; M. R. McKean	2015	Neuromuscular and perceptual fatigue responses to consecutive tag football matches	International Journal of Sports Physiology and Performance	No	Duplicate records
166	L. W. Hogarth; B. J. Burkett; M. R. McKean; T. Gabbett; W. Kraak	2016	Match demands of professional rugby football codes: A review from 2008 to 2015	International Journal of Sports Science & Coaching	No	Duplicate records
167	D. L. Huber; D. G. Thomas; M. Danduran; T. B. Meier; M. A. McCrea; L. D. Nelson	2019	Quantifying activity levels after sport-related concussion using actigraph and mobile (mHealth) technologies	Journal of Athletic Training	No	Duplicate records
168	C. Humberto Almeida; P. Cruz; R. Gonçalves; R. Batalau; P. Paixão; J. António Jorge; P. Vargas	2022	Game criticality in male youth football: Situational and age-related effects on the goal-scoring period in Portuguese national championships. / La criticalidad del juego en el fútbol juvenil masculino: Efectos situacionales y de la edad en el período de gol en los campeonatos nacionales portugueses	Retos: Nuevas Perspectivas de Educación Física, Deporte y Recreación	No	Duplicate records
169	W. Jang; D. H. Kwak; Y. J. Ko	2020	Vitalizing effect of athlete-drafting task in fantasy sports: the role of competitive goal-framing, involvement, and competitiveness trait	European Sport Management Quarterly	No	Duplicate records
170	R. J. Jansen; R. van Egmond; H. de Ridder	2016	Task Prioritization in Dual-Tasking: Instructions versus Preferences	PLOS ONE	No	Duplicate records
171	R. J. Jansen; R. Van Egmond; H. De Ridder	2016	Task prioritization in dual-tasking: Instructions versus preferences	PLoS ONE	No	Duplicate records
172	I. Jukic; J. Calleja-González; F. Cos; F. Cuzzolin; J. Olmo; N. Terrados; N. Njaradi; R. Sassi; B. Requena; L. Milanovic; I. Krakan; K. Chatzichristos; P. E. Alcaraz	2020	Strategies and Solutions for Team Sports Athletes in Isolation due to COVID-19	Sports (Basel)	No	Duplicate records
173	P. Kamarauskas; D. Conte	2022	Changes in salivary markers during basketball long-term and short-term training periods: A systematic review	Biology of Sport	No	Duplicate records

174	A. Khacharem	2017	Top-down and bottom-up guidance in comprehension of schematic football diagrams	JOURNAL OF SPORTS SCIENCES	No	Duplicate records
175	A. Khacharem	2017	Top-down and bottom-up guidance in comprehension of schematic football diagrams	Journal of Sports Sciences	No	Duplicate records
176	A. Khacharem; K. Trabelsi; F. A. Engel; B. Sperlich; S. Kalyuga	2020	The Effects of Temporal Contiguity and Expertise on Acquisition of Tactical Movements	Frontiers in Psychology	No	Duplicate records
177	A. Khacharem; K. Trabelsi; B. Zoudji; S. Kalyuga	2020	Communicating Dynamic Behaviors in Basketball: The Role of Verbal Instructions and Arrow Symbols	Res Q Exerc Sport	No	Duplicate records
178	A. Khacharem; K. Trabelsi; B. Zoudji; S. Kalyuga	2020	Communicating Dynamic Behaviors in Basketball: The Role of Verbal Instructions and Arrow Symbols	RESEARCH QUARTERLY FOR EXERCISE AND SPORT	No	Duplicate records
179	A. Khacharem; K. Trabelsi; B. Zoudji; S. Kalyuga	2020	Communicating Dynamic Behaviors in Basketball: The Role of Verbal Instructions and Arrow Symbols	Research Quarterly for Exercise and Sport	No	Duplicate records
180	A. Khacharem; B. Zoudji; S. Kalyuga	2019	Which representation is best for communicating dynamic information?	Memory	No	Duplicate records
181	A. Kok	2022	Cognitive control, motivation and fatigue: A cognitive neuroscience perspective	BRAIN AND COGNITION	No	Duplicate records
182	A. Kok	2022	Cognitive control, motivation and fatigue: A cognitive neuroscience perspective	Brain and Cognition	No	Duplicate records
183	M. H. Kosack; W. Staiano; R. Folino; M. B. Hansen; S. Lønbro	2020	The Acute Effect of Mental Fatigue on Badminton Performance in Elite Players	Int J Sports Physiol Perform	No	Duplicate records
184	M. H. Kosack; W. Staiano; R. Folino; M. B. Hansen; S. Lonbro	2020	The Acute Effect of Mental Fatigue on Badminton Performance in Elite Players	INTERNATIONAL JOURNAL OF SPORTS PHYSIOLOGY AND PERFORMANCE	No	Duplicate records
185	M. H. Kosack; W. Staiano; R. Folino; M. B. Hansen; S. Lønbro	2020	The acute effect of mental fatigue on badminton performance in elite players	International Journal of Sports Physiology and Performance	No	Duplicate records
186	V. Kostiukevych; N. Lazarenko; V. Adamchuk; N. Shchepotina; T. Vozniuk; O. Shynkaruk; I. Asauliuk; S. Konnov; S. Voitenko	2023	COMPREHENSIVE ASSESSMENT OF THE PREPAREDNESS OF HIGHLY QUALIFIED FIELD HOCKEY PLAYERS AT THE STAGE OF DIRECT PREPARATION FOR THE MAIN COMPETITIONS. / КОМПЛЕКСНА ОЦІНКА ПІДГОТОВЛЕНОСТІ ВИСОКОКВАЛІФІКОВАНИХ ХОКЕЇСТІВ НА ТРАВІ НА ЕТАПІ БЕЗПОСЕРЕДНЬОЇ ПІДГОТОВКИ ДО ГОЛОВНИХ ЗМАГАНЬ	Physical Education Theory & Methodology	No	Duplicate records
187	C. A. Kunrath; F. Y. Nakamura; A. Roca; A. Tessitore; I. Teoldo Da Costa	2020	How does mental fatigue affect soccer performance during small-sided games? A cognitive, tactical and physical approach	Journal of Sports Sciences	No	Duplicate records
188	C. A. Kunrath; F. Y. Nakamura; A. Roca; A. Tessitore; I. Teoldo Da Costa	2020	How does mental fatigue affect soccer performance during small-sided games? A cognitive, tactical and physical approach	J Sports Sci	No	Duplicate records
189	S. Laborde; M. Raab	2013	The tale of hearts and reason: The influence of mood on decision making	Journal of Sport and Exercise Psychology	No	Duplicate records
190	W.-K. Lam; J.-X. Fan; Y. Zheng; W. C.-C. Lee	2019	Joint and plantar loading in table tennis topspin forehand with different footwork	European Journal of Sport Science	No	Duplicate records
191	W. K. Lam; R. S. Masters;	2010	Cognitive demands of error processing	Conscious Cogn	No	Duplicate records

	J. P. Maxwell		associated with preparation and execution of a motor skill			
192	W. K. Lam; R. S. W. Masters; J. P. Maxwell	2010	Cognitive demands of error processing associated with preparation and execution of a motor skill	Consciousness and Cognition	No	Duplicate records
193	S. Lane; N. Hall; L. Keir; C. Lawrence	2020	Empowering Elite Athlete Education in Australian Professional Sports	International Journal of Sport and Society	No	Duplicate records
194	Y. Le Mansec; B. Pageaux; A. Nordez; S. Dorel; M. Jubeau	2018	Mental fatigue alters the speed and the accuracy of the ball in table tennis	J Sports Sci	No	Duplicate records
195	Y. Le Mansec; B. Pageaux; A. Nordez; S. Dorel; M. Jubeau	2018	Mental fatigue alters the speed and the accuracy of the ball in table tennis	JOURNAL OF SPORTS SCIENCES	No	Duplicate records
196	Y. Le Mansec; B. Pageaux; A. Nordez; S. Dorel; M. Jubeau	2018	Mental fatigue alters the speed and the accuracy of the ball in table tennis	Journal of Sports Sciences	No	Duplicate records
197	Y. Le Mansec; J. Perez; Q. Rouault; J. Doron; M. Jubeau	2020	Impaired performance of the smash stroke in badminton induced by muscle fatigue	International Journal of Sports Physiology and Performance	No	Duplicate records
198	J. W. Lee; S. Song; Y. Kim; S. B. Park; D. H. Han	2023	Soccer's AI transformation: deep learning's analysis of soccer's pandemic research evolution	Frontiers in Psychology	No	Duplicate records
199	E. Lignell; V. Rago; M. Mohr	2020	Analysis of goal scoring opportunities in elite male ice hockey in relation to tactical and contextual variables	International Journal of Performance Analysis in Sport	No	Duplicate records
200	J. Low; A. M. Williams; A. McRobert; P. Ford	2013	The microstructure of practice activities engaged in by elite and recreational youth cricket players	Journal of Sports Sciences	No	Duplicate records
201	E. Lundkvist; H. Gustafsson; G. Björklund; P. Davis; A. Ivarsson	2021	Relating Competitive Golfers' Perceived Emotions and Performance	Perceptual and Motor Skills	No	Duplicate records
202	M. Lyons; A.-N. Yahya; J. Hankey; A. Nevill	2013	The Effect of Moderate and High-Intensity Fatigue on Groundstroke Accuracy in Expert and Non-Expert Tennis Players	Journal of Sports Science & Medicine	No	Duplicate records
203	A. Magdaleno; L. Wiersma; B. B. Meyer	2022	An Exploratory Comparison of Subjective Mental Fatigue Following a Task Designed to Replicate the Observation of Game Film	International Journal of Exercise Science	No	Duplicate records
204	H. Makaruk; J. M. Porter; A. Bodasińska; S. Palmer	2020	Optimizing the penalty kick under external focus of attention and autonomy support instructions	European Journal of Sport Science	No	Duplicate records
205	J. M. Malheiro Maia Junior; D. Braga de Mello; G. Rosa; L. Alonso dos Santos; R. A. Moreira Nunes; R. G. de Souza Vale	2023	Effects of scoring method on the physical, technical, and tactical performances during football smallsided games (SSGs): A systematic review. / Efectos del método de puntuación en el desempeño físico, técnico y táctico durante los juegos de fútbol en espacios reducidos (SSGs): una revisión sistemática	Retos: Nuevas Perspectivas de Educación Física, Deporte y Recreación	No	Duplicate records
206	N. Maraga; R. Duffield; D. Gescheit; T. Perri; M. Reid	2018	Playing not once, not twice but three times in a day: the effect of fatigue on performance in junior tennis players	International Journal of Performance Analysis in Sport	No	Duplicate records
207	G. Marcolin; N. Camazzola; F. A. Panizzolo; D. Grigoletto; A. Paoli	2018	Different intensities of basketball drills affect jump shot accuracy of expert and junior players	PeerJ	No	Duplicate records
208	Y. Mariano; K. Martin; J. Mara	2023	Mental fatigue and technical performance in elite rugby league	JOURNAL OF SPORTS SCIENCES	No	Duplicate records
209	Y. Mariano; K. Martin; J.	2023	Mental fatigue and technical performance in	Journal of Sports Sciences	No	Duplicate records

	Mara		elite rugby league			
210	R. U. I. Marques; F. Martins; R. U. I. Mendes; M. C. E. Silva; G. Dias	2018	The use of eye tracking glasses in Basketball shooting: a systematic review	Journal of Physical Education & Sport	No	Duplicate records
211	D. Marqués-Jiménez; J. Calleja-González; I. Arratibel; A. Delextrat; N. Terrados	2017	Fatigue and recovery in soccer: Evidence and challenges	Open Sports Sciences Journal	No	Duplicate records
212	R. J. Mason; D. Farrow; J. A. C. Hattie	2020	An analysis of in-game feedback provided by coaches in an Australian Football League competition	Physical Education and Sport Pedagogy	No	Duplicate records
213	E. Maunder; A. E. Kilding; S. P. Cairns	2017	Do Fast Bowlers Fatigue in Cricket? A Paradox Between Player Anecdotes and Quantitative Evidence	International Journal of Sports Physiology & Performance	No	Duplicate records
214	E. Maunder; A. E. Kilding; S. P. Cairns	2017	Do Fast Bowlers Fatigue in Cricket? A Paradox Between Player Anecdotes and Quantitative Evidence	Int J Sports Physiol Perform	No	Duplicate records
215	E. Maunder; A. E. Kilding; S. P. Cairns	2017	Do Fast Bowlers Fatigue in Cricket? A Paradox Between Player Anecdotes and Quantitative Evidence	INTERNATIONAL JOURNAL OF SPORTS PHYSIOLOGY AND PERFORMANCE	No	Duplicate records
216	E. Maunder; A. E. Kilding; S. P. Cairns	2017	Do fast bowlers fatigue in cricket? A paradox between player anecdotes and quantitative evidence	International Journal of Sports Physiology and Performance	No	Duplicate records
217	S. Mellalieu; G. Trewartha; K. Stokes	2008	Science and rugby union	Journal of Sports Sciences	No	Duplicate records
218	D. Mexis; T. Nomikos; N. Mitsopoulos; N. Kostopoulos	2023	Effect of a 6-Week Preseason Training Protocol on Physiological and Muscle Damage Markers in High-Level Female and Male Basketball Players	Sports	No	Duplicate records
219	A. Moreira; M. S. Aoki; E. Franchini; D. G. da Silva Machado; A. C. Paludo; A. H. Okano	2018	Mental fatigue impairs technical performance and alters neuroendocrine and autonomic responses in elite young basketball players	Physiology and Behavior	No	Duplicate records
220	A. Moreira; M. S. Aoki; E. Franchini; D. G. D. Machado; A. C. Paludo; A. H. Okano	2018	Mental fatigue impairs technical performance and alters neuroendocrine and autonomic responses in elite young basketball players	PHYSIOLOGY & BEHAVIOR	No	Duplicate records
221	A. Moreira; L. Moscaleski; D. G. D. Machado; M. Bikson; G. Unal; P. S. Bradley; T. Cevada; F. T. G. da Silva; A. F. Baptista; E. Morya; A. H. Okano	2023	Transcranial direct current stimulation during a prolonged cognitive task: the effect on cognitive and shooting performances in professional female basketball players	ERGONOMICS	No	Duplicate records
222	J. Morente-Sánchez; T. Zandonai; M. Mateo-March; D. Sanabria; C. Sánchez-Muñoz; C. Chiamulera; M. Z. Díaz	2015	Acute effect of Snus on physical performance and perceived cognitive load on amateur footballers	SCANDINAVIAN JOURNAL OF MEDICINE & SCIENCE IN SPORTS	No	Duplicate records
223	J. Morente-Sánchez; T. Zandonai; M. Mateo-March; D. Sanabria; C. Sánchez-Muñoz; C. Chiamulera; M. Zabala Díaz	2015	Acute effect of Snus on physical performance and perceived cognitive load on amateur footballers	Scandinavian Journal of Medicine and Science in Sports	No	Duplicate records

224	J. Morente-Sánchez; T. Zandonai; M. Mateo-March; D. Sanabria; C. Sánchez-Muñoz; C. Chiamulera; M. Zabala Díaz	2015	Acute effect of Snus on physical performance and perceived cognitive load on amateur footballers	Scand J Med Sci Sports	No	Duplicate records
225	R. Mullen; L. Hardy; A. Tattersall	2005	The effects of anxiety on motor performance: A test of the conscious processing hypothesis	Journal of Sport and Exercise Psychology	No	Duplicate records
226	T. Mullen; C. Twist; M. Daniels; N. Dobbin; J. Highton	2021	Influence of contextual factors, technical performance, and movement demands on the subjective task load associated with professional rugby league match-play	International Journal of Sports Physiology and Performance	No	Duplicate records
227	A. P. Murphy; R. Duffield; A. Kellett; M. Reid	2014	Comparison of athlete-coach perceptions of internal and external load markers for elite junior tennis training	Int J Sports Physiol Perform	No	Duplicate records
228	A. P. Murphy; R. Duffield; A. Kellett; M. Reid	2014	Comparison of Athlete-Coach Perceptions of Internal and External Load Markers for Elite Junior Tennis Training	INTERNATIONAL JOURNAL OF SPORTS PHYSIOLOGY AND PERFORMANCE	No	Duplicate records
229	A. P. Murphy; R. Duffield; A. Kellett; M. Reid	2014	Comparison of athlete-coach perceptions of internal and external load markers for elite junior tennis training	International Journal of Sports Physiology and Performance	No	Duplicate records
230	A. P. Murphy; R. Duffield; A. Kellett; M. Reid	2014	A descriptive analysis of internal and external loads for elite-level tennis drills	Int J Sports Physiol Perform	No	Duplicate records
231	A. P. Murphy; R. Duffield; A. Kellett; M. Reid	2014	A Descriptive Analysis of Internal and External Loads for Elite-Level Tennis Drills	INTERNATIONAL JOURNAL OF SPORTS PHYSIOLOGY AND PERFORMANCE	No	Duplicate records
232	A. P. Murphy; R. Duffield; A. Kellett; M. Reid	2014	A descriptive analysis of internal and external loads for elite-level tennis drills	International Journal of Sports Physiology and Performance	No	Duplicate records
233	A. P. Murphy; R. Duffield; A. Kellett; M. Reid	2016	A Comparison of the Perceptual and Technical Demands of Tennis Training, Simulated Match Play, and Competitive Tournaments	Int J Sports Physiol Perform	No	Duplicate records
234	A. P. Murphy; R. Duffield; A. Kellett; M. Reid	2016	A Comparison of the Perceptual and Technical Demands of Tennis Training, Simulated Match Play, and Competitive Tournaments	INTERNATIONAL JOURNAL OF SPORTS PHYSIOLOGY AND PERFORMANCE	No	Duplicate records
235	A. P. Murphy; R. Duffield; A. Kellett; M. Reid	2016	A comparison of the perceptual and technical demands of tennis training, simulated match play, and competitive tournaments	International Journal of Sports Physiology and Performance	No	Duplicate records
236	M. A. Nabli; N. Ben Abdelkrim; M. S. Fessi; M. D. DeLang; W. Moalla; K. Chamari	2019	Sport science applied to basketball refereeing: a narrative review	PHYSICIAN AND SPORTSMEDICINE	No	Duplicate records
237	M. A. Nabli; N. Ben Abdelkrim; M. S. Fessi; M. D. DeLang; W. Moalla; K. Chamari	2019	Sport science applied to basketball refereeing: a narrative review	Physician and Sportsmedicine	No	Duplicate records
238	G. P. Nassis; A. Massey; P. Jacobsen; J. Brito; M. B. Randers; C. Castagna; M. Mohr; P. Krustrup	2020	Elite football of 2030 will not be the same as that of 2020: Preparing players, coaches, and support staff for the evolution	Scandinavian Journal of Medicine and Science in Sports	No	Duplicate records
239	M. Nedelec; G. Dupont	2019	The influence of playing position in soccer on the recovery kinetics of cognitive and physical performance	Journal of Sports Medicine and Physical Fitness	No	Duplicate records

240	M. Nédélec; S. Halson; A. Abaidia; S. Ahmaidi; G. Dupont	2015	Stress, Sleep and Recovery in Elite Soccer: A Critical Review of the Literature	SPORTS MEDICINE	No	Duplicate records
241	L. Nielsen	2014	The Games Approach and High School Football	Sport Journal	No	Duplicate records
242	R. Notario-Alonso; A. Prieto-Ayuso; A. García-Notario; O. R. Contreras-Jordán	2023	The sports commitment in football players and its relationship with the coach performance: A systematic review	Journal of Human Sport and Exercise	No	Duplicate records
243	R. O'Meagher; J. O'Reilly; A. Ali	2022	The effect of different playing surfaces on soccer skill performance	International Journal of Sports Science & Coaching	No	Duplicate records
244	A. Oliver; P. J. McCarthy; L. Burns	2020	A Grounded-Theory Study of Meta-Attention in Golfers	SPORT PSYCHOLOGIST	No	Duplicate records
245	A. Oliver; P. J. McCarthy; L. Burns	2020	A grounded-theory study of meta-attention in golfers	Sport Psychologist	No	Duplicate records
246	A. S. Parodi-Feye; Á. D. Cappuccio-Díaz; C. A. Magallanes-Mira	2023	Effects of Inspiratory Muscle Training on Physiological Performance Variables in Women's Handball	Journal of Human Kinetics	No	Duplicate records
247	A. Peeters; C. Carling; J. Piscione; M. Lacome	2019	In-Match Physical Performance Fluctuations in International Rugby Sevens Competition	Journal of Sports Science & Medicine	No	Duplicate records
248	A. Peeters; C. Carling; J. Piscione; M. Lacome	2019	In-match physical performance fluctuations in international rugby sevens competition	Journal of Sports Science and Medicine	No	Duplicate records
249	E. M. Penna; E. Filho; B. T. Campos; D. A. Pires; F. Y. Nakamura; T. T. Mendes; T. R. Lopes; M. Smith; L. S. Prado	2018	Mental fatigue does not affect heart rate recovery but impairs performance in handball players	Revista Brasileira de Medicina do Esporte	No	Duplicate records
250	M. Peralta-Geis; J. Arboix-Alió; J. C. Sanromà; R. M. Agulled	2021	Influence of fatigue in decision-making in football referees	Sport TK	No	Duplicate records
251	Y. Pessoa Da Costa; L. Fortes; R. Santos; E. Souza; L. Hayes; E. Soares-Silva; G. Ricarte Batista	2023	Mental fatigue measured in real-world sport settings: A case study of world class beach volleyball players	Journal of Physical Education & Sport	No	Duplicate records
252	I. Popovych; A. Kurova; I. Koval; V. Kazibekova; M. Maksymov; V. Huzar	2022	Interdependence of emotionality, anxiety, aggressiveness and subjective control in handball referees before the beginning of a game: a comparative analysis	Journal of Physical Education and Sport	No	Duplicate records
253	I. Popovych; I. Radul; V. Radul; I. Geiko; N. Hoi; O. Sribna; Y. Tymosh	2022	Construction and comparison of mental resource complexes of male and female sports teams	Journal of Physical Education and Sport	No	Duplicate records
254	L. Pote; S. Nicholls; G. King; C. Christie	2023	Anthropometric and morphological characteristics of elite male cricket bowlers and batters over time: A systematic review	International Journal of Sports Science and Coaching	No	Duplicate records
255	G. M. Praça; R. B. E. Sousa; P. J. Greco	2019	Influence of aerobic power on youth players' tactical behavior and network properties during football small-sided games	Sports	No	Duplicate records
256	W. Pramkratok; T. Yimlamai	2021	Effects of Acute Hypoxia on Psycho-Physiological Response and Muscle Oxygenation during Incremental Running Exercise	Journal of Exercise Physiology Online	No	Duplicate records
257	J. M. Pratas; A. Volossovitch; A. I. Carita	2018	Goal scoring in elite male football: A systematic review	Journal of Human Sport and Exercise	No	Duplicate records
258	S. A. Pullinger; P. S. Bradley; J. Causer; P. R. Ford; A. Newlove; K. Patel; K. Reid; C. M.	2019	Football-induced fatigue in hypoxia impairs repeated sprint ability and perceptual-cognitive skills	SCIENCE AND MEDICINE IN FOOTBALL	No	Duplicate records

	Robertson; J. G. Burniston; D. A. Doran; J. M. Waterhouse; B. J. Edwards					
259	S. A. Pullinger; P. S. Bradley; J. Causer; P. R. Ford; A. Newlove; K. Patel; K. Reid; C. M. Robertson; J. G. Burniston; D. A. Doran; J. M. Waterhouse; B. J. Edwards	2019	Football-induced fatigue in hypoxia impairs repeated sprint ability and perceptual-cognitive skills	Science and Medicine in Football	No	Duplicate records
260	K. Put; J. Wagemans; J. Spitz; A. M. Williams; W. F. Helsen	2016	Using web-based training to enhance perceptual-cognitive skills in complex dynamic offside events	Journal of Sports Sciences	No	Duplicate records
261	D. M. S. Rashid; S. M. S. Faraj; N. Hedayatpour	2020	The effect of triceps brachii fatigue on shot accuracy of male and female basketball players	International Journal of Performance Analysis in Sport	No	Duplicate records
262	B. Reid; K. Schreiber; J. Shawhan; E. Stewart; R. Burch; W. Reimann	2020	Reaction time assessment for coaching defensive players in NCAA division 1 American football: A comprehensive literature review	International Journal of Industrial Ergonomics	No	Duplicate records
263	G. Rekik; Y. Belkhir; M. Jarraya	2021	Searching to improve learning from complex animated basketball scenes: when decreasing the presentation speed is more efficient than using segmentation	Technology, Pedagogy and Education	No	Duplicate records
264	G. Rekik; Y. Belkhir; N. Mezghanni; M. Jarraya; Y. S. Chen; C. D. Kuo	2021	Learning Basketball Tactical Actions from Video Modeling and Static Pictures: When Gender Matters	CHILDREN-BASEL	No	Duplicate records
265	G. Rekik; Y. Belkhir; N. Mezghanni; M. Jarraya; Y. S. Chen; C. D. Kuo	2021	Learning basketball tactical actions from video modeling and static pictures: When gender matters	Children	No	Duplicate records
266	G. Rekik; A. Khacharem; Y. Belkhir; N. Bali; M. Jarraya	2019	The instructional benefits of dynamic visualizations in the acquisition of basketball tactical actions	Journal of Computer Assisted Learning	No	Duplicate records
267	G. Rekik; N. Khacharem; Y. Belkhir; N. Bali; M. Jarraya	2019	The effect of visualization format and content complexity on acquisition of tactical actions in basketball	LEARNING AND MOTIVATION	No	Duplicate records
268	M. A. Rendell; R. S. Masters; D. Farrow; T. Morris	2011	An implicit basis for the retention benefits of random practice	J Mot Behav	No	Duplicate records
269	M. A. Rendell; R. S. W. Masters; D. Farrow; T. Morris	2011	An implicit basis for the retention benefits of random practice	Journal of Motor Behavior	No	Duplicate records
270	M. A. Rendell; R. S. W. Masters; D. Farrow; T. Morris	2011	An Implicit Basis for the Retention Benefits of Random Practice	JOURNAL OF MOTOR BEHAVIOR	No	Duplicate records
271	V. Richard; B. Lavoie-Léonard; T. Romeas	2021	Embedding Perceptual–Cognitive Training in the Athlete Environment: An Interdisciplinary Case Study Among Elite Female Goalkeepers Preparing for Tokyo 2020	Case Studies in Sport & Exercise Psychology	No	Duplicate records
272	J. C. Rius; D. Suarez	2016	The specific nature of endurance training in tennis	Coaching & Sport Science Review (Spanish Version)	No	Duplicate records
273	M. K. Robison; B. Nguyen	2023	Competition and Reward Structures Nearly Eliminate Time-on-Task Performance Decrements: Implications for Theories of Vigilance and Mental Effort	JOURNAL OF EXPERIMENTAL PSYCHOLOGY-HUMAN PERCEPTION AND	No	Duplicate records

				PERFORMANCE		
274	M. K. Robison; B. Nguyen	2023	Competition and reward structures nearly eliminate time-on-task performance decrements: Implications for theories of vigilance and mental effort	Journal of experimental psychology. Human perception and performance	No	Duplicate records
275	O. R. Runswick; M. Jewiss; B. Sharpe; J. S. North	2021	Context Affects Quiet Eye Duration and Motor Performance Independent of Cognitive Effort	JOURNAL OF SPORT & EXERCISE PSYCHOLOGY	No	Duplicate records
276	O. R. Runswick; M. Jewiss; B. T. Sharpe; J. S. North	2021	Context Affects Quiet Eye Duration and Motor Performance Independent of Cognitive Effort	J Sport Exerc Psychol	No	Duplicate records
277	O. R. Runswick; M. Jewiss; B. T. Sharpe; J. S. North	2021	Context affects quiet eye duration and motor performance independent of cognitive effort	Journal of Sport and Exercise Psychology	No	Duplicate records
278	A. Rusdiana; A. Komaini; S. Nugraha	2023	Impact of cardiovascular fatigue on kinematic changes in badminton overhead jump smash: A descriptive analysis	Journal of Physical Education and Sport	No	Duplicate records
279	S. Russell; D. Jenkins; S. Halson; V. Kelly	2020	Changes in subjective mental and physical fatigue during netball games in elite development athletes	Journal of Science and Medicine in Sport	No	Duplicate records
280	S. Russell; D. G. Jenkins; S. L. Halson; L. E. Juliff; M. J. Connick; V. G. Kelly	2022	Mental Fatigue Over 2 Elite Netball Seasons: A Case for Mental Fatigue to be Included in Athlete Self-Report Measures	International Journal of Sports Physiology and Performance	No	Duplicate records
281	S. Russell; D. G. Jenkins; S. L. Halson; V. G. Kelly	2022	Mental fatigue increases across a 16-week pre-season in elite female athletes	Journal of Science and Medicine in Sport	No	Duplicate records
282	S. Ryan; A. J. Coutts; J. Hocking; T. Kempton	2017	Factors affecting match running performance in professional Australian football	International Journal of Sports Physiology and Performance	No	Duplicate records
283	S. Ryan; T. Kempton; F. M. Impellizzeri; A. J. Coutts	2020	Training monitoring in professional Australian football: theoretical basis and recommendations for coaches and scientists	SCIENCE AND MEDICINE IN FOOTBALL	No	Duplicate records
284	S. Ryan; T. Kempton; F. M. Impellizzeri; A. J. Coutts	2020	Training monitoring in professional Australian football: theoretical basis and recommendations for coaches and scientists	Science and Medicine in Football	No	Duplicate records
285	R. D. Samuel; C. Englert; Q. Zhang; I. Basevitch	2018	Hi ref, are you in control? Self-control, ego-depletion, and performance in soccer referees	Psychology of Sport and Exercise	No	Duplicate records
286	P. Sansone; A. Tessitore; I. Lukonaitiene; H. Paulauskas; H. Tschan; D. Conte	2020	Technical-tactical profile, perceived exertion, mental demands and enjoyment of different tactical tasks and training regimes in basketball small-sided games	Biology of Sport	No	Duplicate records
287	S. Santos; D. Coutinho; B. Gonçalves; E. Abade; B. Pasquarelli; J. Sampaio	2020	Effects of manipulating ball type on youth footballers' performance during small-sided games	International Journal of Sports Science and Coaching	No	Duplicate records
288	J. M. Sarabia; J. Fernandez-Fernandez; C. Juan-Recio; H. Hernández-Davó; T. Urbán; M. Moya	2015	Mechanical, Hormonal and Psychological Effects of a Non-Failure Short-Term Strength Training Program in Young Tennis Players	Journal of Human Kinetics	No	Duplicate records
289	S. Schaefer; D. Scornaienchi	2020	Table Tennis Experts Outperform Novices in a Demanding Cognitive-Motor Dual-Task Situation	Journal of Motor Behavior	No	Duplicate records
290	J. M. H. Schellekens; G. J. Sijtsma; E. Vegter; T. F. Meijman	2000	Immediate and delayed after-effects of long lasting mentally demanding work	Biological Psychology	No	Duplicate records
291	P. Scott; R. Ahmun; C. de Weymarn; E. Gardner; A. Bliss; T. W. Jones; S. J. Callaghan; J. Tallent	2023	Evolution of anthropometric and physical performance characteristics of international male cricketers from 2014 to 2020 in a World Cup winning nation	International Journal of Sports Science and Coaching	No	Duplicate records

292	H. Sepahvand; G. P. Jahromi; H. Sahraei; G. H. Meftahi	2017	Studying the perceptive and cognitive function under the stress of match in female futsal players	Asian Journal of Sports Medicine	No	Duplicate records
293	F. Shaabani; A. Naderi; E. Borella; L. Calmeiro	2020	Does a brief mindfulness intervention counteract the detrimental effects of ego depletion in basketball free throw under pressure?	Sport, Exercise, and Performance Psychology	No	Duplicate records
294	S. Shao; C. Yu; Y. Song; J. S. Baker; U. C. Ugbolue; I. M. Lanzoni; Y. Gu	2020	Mechanical character of lower limb for table tennis cross step maneuver	International Journal of Sports Science and Coaching	No	Duplicate records
295	T. Shcherbak; I. Popovych; A. Kariyev; A. Duisenbayeva; V. Huzar; I. Hoian; K. Kyrychenko	2023	Psychological causes of fatigue in football players	Journal of Physical Education and Sport	No	Duplicate records
296	M. Shin; Y. Kim; S. Park	2019	Effects of State Anxiety and Ego Depletion on Performance Change in Golf Putting: A Hierarchical Linear Model Application	Percept Mot Skills	No	Duplicate records
297	M. Shin; Y. Kim; S. Park	2019	Effects of State Anxiety and Ego Depletion on Performance Change in Golf Putting: A Hierarchical Linear Model Application	PERCEPTUAL AND MOTOR SKILLS	No	Duplicate records
298	M. Shin; Y. Kim; S. Park	2019	Effects of State Anxiety and Ego Depletion on Performance Change in Golf Putting: A Hierarchical Linear Model Application	Perceptual and Motor Skills	No	Duplicate records
299	H. Silva; F. Y. Nakamura; M. Beato; R. Marcelino	2023	Acceleration and deceleration demands during training sessions in football: a systematic review	Science and Medicine in Football	No	Duplicate records
300	F. Silvestri; M. Campanella; M. Bertollo; M. R. Albuquerque; V. Bonavolontà; F. Perroni; C. Baldari; L. Guidetti; D. Curzi	2023	Acute Effects of Fitlight Training on Cognitive-Motor Processes in Young Basketball Players	INTERNATIONAL JOURNAL OF ENVIRONMENTAL RESEARCH AND PUBLIC HEALTH	No	Duplicate records
301	F. Silvestri; M. Campanella; M. Bertollo; M. R. Albuquerque; V. Bonavolontà; F. Perroni; C. Baldari; L. Guidetti; D. Curzi	2023	Acute Effects of Fitlight Training on Cognitive-Motor Processes in Young Basketball Players	International Journal of Environmental Research and Public Health	No	Duplicate records
302	M. R. Smith; A. J. Coutts; M. Merlini; D. Deprez; M. Lenoir; S. M. Marcora	2016	Mental fatigue impairs soccer-specific physical and technical performance	Medicine and Science in Sports and Exercise	No	Duplicate records
303	M. R. Smith; J. Fransen; D. Deprez; M. Lenoir; A. J. Coutts	2017	Impact of mental fatigue on speed and accuracy components of soccer-specific skills	Science and Medicine in Football	No	Duplicate records
304	M. R. Smith; L. Zeuwts; M. Lenoir; N. Hens; L. M. De Jong; A. J. Coutts	2016	Mental fatigue impairs soccer-specific decision-making skill	J Sports Sci	No	Duplicate records
305	M. R. Smith; L. Zeuwts; M. Lenoir; N. Hens; L. M. S. De Jong; A. J. Coutts	2016	Mental fatigue impairs soccer-specific decision-making skill	Journal of Sports Sciences	No	Duplicate records
306	N. C. Smith; M. Bellamy; D. J. Collins; D. Newell	2001	A test of processing efficiency theory in a team sport context	J Sports Sci	No	Duplicate records
307	N. C. Smith; M. Bellamy; D. J. Collins; D. Newell	2001	A test of processing efficiency theory in a team sport context	JOURNAL OF SPORTS SCIENCES	No	Duplicate records
308	N. C. Smith; M. Bellamy; D. J. Collins; D. Newell	2001	A test of processing efficiency theory in a team sport context	Journal of Sports Sciences	No	Duplicate records
309	N. A. Sothern; J.	2021	Exploring the mental health and wellbeing of	Soccer & Society	No	Duplicate records

	O'Gorman		professional academy footballers in England			
310	W. Staiano; M. Merlini; M. Romagnoli; U. Kirk; C. Ring; S. Marcora	2022	Brain Endurance Training Improves Physical, Cognitive, and Multitasking Performance in Professional Football Players	Int J Sports Physiol Perform	No	Duplicate records
311	W. Staiano; M. Merlini; M. Romagnoli; U. Kirk; C. Ring; S. Marcora	2022	Brain Endurance Training Improves Physical, Cognitive, and Multitasking Performance in Professional Football Players	INTERNATIONAL JOURNAL OF SPORTS PHYSIOLOGY AND PERFORMANCE	No	Duplicate records
312	W. Staiano; M. Merlini; M. Romagnoli; U. Kirk; C. Ring; S. Marcora	2022	Brain Endurance Training Improves Physical, Cognitive, and Multitasking Performance in Professional Football Players	International Journal of Sports Physiology and Performance	No	Duplicate records
313	N. Stanger; R. Chettle; J. Whittle; J. Poolton	2018	The role of preperformance and in-game emotions in cognitive interference during sport performance: The moderating role of self-confidence and reappraisal	Sport Psychologist	No	Duplicate records
314	E. J. Stevenson; P. R. Hayes; S. J. Allison	2009	The effect of a carbohydrate-caffeine sports drink on simulated golf performance	Appl Physiol Nutr Metab	No	Duplicate records
315	E. J. Stevenson; P. R. Hayes; S. J. Allison	2009	The effect of a carbohydrate-caffeine sports drink on simulated golf performance	APPLIED PHYSIOLOGY NUTRITION AND METABOLISM-PHYSIOLOGIE APPLIQUEE NUTRITION ET METABOLISME	No	Duplicate records
316	E. J. Stevenson; P. R. Hayes; S. J. Allison	2009	The effect of a carbohydrate-caffeine sports drink on simulated golf performance	Applied Physiology, Nutrition and Metabolism	No	Duplicate records
317	S. Sukys; I. Tilindienė; V. J. Cesnaitiene; R. Kreivyte	2019	Does Emotional Intelligence Predict Athletes' Motivation to Participate in Sports?	Perceptual and Motor Skills	No	Duplicate records
318	H. Sun; K. G. Soh; S. Roslan; M. Wazir; K. L. Soh	2021	Does mental fatigue affect skilled performance in athletes? A systematic review	PLOS ONE	No	Duplicate records
319	H. Sun; K. G. Soh; S. Roslan; M. Wazir; K. L. Soh	2021	Does mental fatigue affect skilled performance in athletes? A systematic review	PLoS One	No	Duplicate records
320	M. Suzuki; T. Umeda; S. Nakaji; T. Shimoyama; T. Mashiko; K. Sugawara	2004	Effect of incorporating low intensity exercise into the recovery period after a rugby match	BRITISH JOURNAL OF SPORTS MEDICINE	No	Duplicate records
321	M. Suzuki; T. Umeda; S. Nakaji; T. Shimoyama; T. Mashiko; K. Sugawara	2004	Effect of incorporating low intensity exercise into the recovery period after a rugby match	British Journal of Sports Medicine	No	Duplicate records
322	J. M. Tassi; J. Díaz-García; M. Á. López-Gajardo; A. Rubio-Morales; T. García-Calvo	2023	Effect of a Four-Week Soccer Training Program Using Stressful Constraints on Team Resilience and Precompetitive Anxiety	International Journal of Environmental Research and Public Health	No	Duplicate records
323	J. M. Tassi; M. A. López-Gajardo; J. Díaz-García; T. García-Calvo; I. González-Ponce	2023	Attentional focus in team sports: Effects of an intervention program on football players	European Journal of Human Movement	No	Duplicate records
324	B. Thatcher; G. Ivanov; M. Szerovay; G. Mills	2021	Virtual Reality Technology in Football Coaching: Barriers and Opportunities	International Sport Coaching Journal	No	Duplicate records
325	C. J. Thompson; J. Fransen; S. Skorski; M. R. Smith; T. Meyer; S. Barrett; A. J. Coutts	2019	Mental Fatigue in Football: Is it Time to Shift the Goalposts? An Evaluation of the Current Methodology	SPORTS MEDICINE	No	Duplicate records
326	C. J. Thompson; J. Fransen; S. Skorski; M. R. Smith; T. Meyer; S.	2019	Mental Fatigue in Football: Is it Time to Shift the Goalposts? An Evaluation of the Current Methodology	Sports Medicine	No	Duplicate records

	Barrett; A. J. Coutts					
327	C. J. Thompson; A. Smith; A. J. Coutts; S. Skorski; N. Datson; M. R. Smith; T. Meyer	2022	Understanding the Presence of Mental Fatigue in Elite Female Football	Res Q Exerc Sport	No	Duplicate records
328	C. J. Thompson; A. Smith; A. J. Coutts; S. Skorski; N. Datson; M. R. Smith; T. Meyer	2022	Understanding the Presence of Mental Fatigue in Elite Female Football	RESEARCH QUARTERLY FOR EXERCISE AND SPORT	No	Duplicate records
329	C. J. Thompson; A. Smith; A. J. Coutts; S. Skorski; N. Datson; M. R. Smith; T. Meyer	2022	Understanding the Presence of Mental Fatigue in Elite Female Football	Research Quarterly for Exercise and Sport	No	Duplicate records
330	H. R. Thornton; J. Miller; L. Taylor; C. Sargent; M. Lastella; P. M. Fowler	2018	Impact of short- compared to long-haul international travel on the sleep and wellbeing of national wheelchair basketball athletes	Journal of Sports Sciences	No	Duplicate records
331	J. Torrado; C. Arce; A. Vales-Vázquez; A. Areces; G. Iglesias; I. Valle; G. Patiño	2017	Relationship between Leadership among Peers and Burnout in Sports Teams	Spanish Journal of Psychology	No	Duplicate records
332	J. Van Cutsem; K. De Pauw; C. Vandervaeren; S. Marcora; R. Meeusen; B. Roelands	2019	Mental fatigue impairs visuomotor response time in badminton players and controls	Psychology of Sport and Exercise	No	Duplicate records
333	V. Vanessa Wergin; Z. Zimanyi; J. Beckmann	2021	A field study investigating running distance and affect of field hockey players in collective team collapse situations	International Journal of Sport and Exercise Psychology	No	Duplicate records
334	D. Veness; S. D. Patterson; O. Jeffries; M. Waldron	2017	The effects of mental fatigue on cricket-relevant performance among elite players	J Sports Sci	No	Duplicate records
335	D. Veness; S. D. Patterson; O. Jeffries; M. Waldron	2017	The effects of mental fatigue on cricket-relevant performance among elite players	JOURNAL OF SPORTS SCIENCES	No	Duplicate records
336	D. Veness; S. D. Patterson; O. Jeffries; M. Waldron	2017	The effects of mental fatigue on cricket-relevant performance among elite players	Journal of Sports Sciences	No	Duplicate records
337	R. Venter	2014	Perceptions of team athletes on the importance of recovery modalities	European Journal of Sport Science	No	Duplicate records
338	J. Vera; R. Jiménez; B. Redondo; I. Madinabeitia; I. Madinabeitia; F. A. López; D. Cárdenas	2019	Intraocular Pressure as an Indicator of the Level of Induced Anxiety in Basketball	OPTOMETRY AND VISION SCIENCE	No	Duplicate records
339	J. Vera; R. Molina; D. Cárdenas; B. Redondo; R. Jiménez	2020	Basketball free-throws performance depends on the integrity of binocular vision	European Journal of Sport Science	No	Duplicate records
340	T. Vogt; S. Gassen; S. Wrede; J. Spielmann; M. Jedrusiak-Jung; S. Härtel; J. Mayer	2018	Football practice with youth players in the “Footbonaut”: Speed of action and ball control in face of physical and mental strain	German Journal of Exercise and Sport Research	No	Duplicate records
341	N. S. Weerakkody; C. J. Taylor; C. L. Bulmer; D. B. Hamilton; J. Gloury; N. J. O'Brien; J. H. Saunders; S. Harvey; T. A. Patterson	2021	The effect of mental fatigue on the performance of Australian football specific skills amongst amateur athletes	JOURNAL OF SCIENCE AND MEDICINE IN SPORT	No	Duplicate records
342	N. S. Weerakkody; C. J. Taylor; C. L. Bulmer; D. B. Hamilton; J. Gloury; N.	2021	The effect of mental fatigue on the performance of Australian football specific skills amongst amateur athletes	Journal of Science and Medicine in Sport	No	Duplicate records

	J. O'Brien; J. H. Saunders; S. Harvey; T. A. Patterson					
343	A. M. Williams; J. Vickers; S. Rodrigues	2002	The effects of anxiety on visual search, movement kinematics, and performance in table tennis: A test of Eysenck and Calvo's processing efficiency theory	JOURNAL OF SPORT & EXERCISE PSYCHOLOGY	No	Duplicate records
344	A. M. Williams; J. Vickers; S. Rodrigues	2002	The effects of anxiety on visual search, movement kinematics, and performance in table tennis: A test of Eysenck and Calvo's processing efficiency theory	Journal of Sport and Exercise Psychology	No	Duplicate records
345	M. Wilson; N. C. Smith	2007	A test of the predictions of processing efficiency theory during elite team competition using the thought occurrence questionnaire for sport	International Journal of Sport Psychology	No	Duplicate records
346	M. J. Wright; D. T. Bishop; R. C. Jackson; B. Abernethy	2013	Brain regions concerned with the identification of deceptive soccer moves by higher-skilled and lower-skilled players	FRONTIERS IN HUMAN NEUROSCIENCE	No	Duplicate records
347	M. J. Wright; D. T. Bishop; R. C. Jackson; B. Abernethy	2013	Brain regions concerned with the identification of deceptive soccer moves by higher-skilled and lower-skilled players	Frontiers in Human Neuroscience	No	Duplicate records
348	L. Yang; Y. C. Wang	2023	The effect of motivational and instructional self-talk on attentional control under noise distraction	PLOS ONE	No	Duplicate records
349	R. Yuan; H. Sun; K. G. Soh; A. Mohammadi; Z. Toumi; Z. Zhang	2023	The effects of mental fatigue on sport-specific motor performance among team sport athletes: A systematic scoping review	Frontiers in Psychology	No	Duplicate records
350	R. Yuan; H. Sun; K. G. Soh; A. Mohammadi; Z. Toumi; Z. D. Zhang	2023	The effects of mental fatigue on sport-specific motor performance among team sport athletes: A systematic scoping review	FRONTIERS IN PSYCHOLOGY	No	Duplicate records
351	M. Zanin; J. Ranaweera; J. Darrall-Jones; D. Weaving; K. Till; G. Roe	2021	A systematic review of small sided games within rugby: Acute and chronic effects of constraints manipulation	Journal of Sports Sciences	No	Duplicate records
352	T. N. Ziegenfuss; S. M. Habowski; R. Lemieux; J. E. Sandrock; A. W. Kedia; C. M. Kerksick; H. L. Lopez	2015	Effects of a dietary supplement on golf drive distance and functional indices of golf performance	Journal of the International Society of Sports Nutrition	No	Duplicate records
353		1985	Cues, Cricket, and Decision-Making	Journal of Sport Psychology	No	Before January1,2014
354		1985	Stress and Psycho-Regulation in Tennis	Journal of Sport Psychology	No	Before January1,2014
355		2000	Team reports	Sporting News	No	Before January1,2014
356		2006	Telic Dominance and Emotional Response in Basketball and Running	Journal of Sport & Exercise Psychology	No	Before January1,2014
357		2008	ROUND 14 PREVIEWS	Rugby League Week	No	Before January1,2014
358		2011	Learn To Be A Clutch Putter	Golf Digest	No	Before January1,2014
359		2013	Sport and Exercise Psychology	Journal of Sport & Exercise Psychology	No	Before January1,2014
360	P. Agrawal; C. Liu; N. Sarkar	2008	Interaction between human and robot: An effect-inspired approach	Interaction Studies	No	Before January1,2014
361	A. Alaphilippe; S. Mandigout; S. B. Ratel; J. Bonis; D. Courteix; M.	2012	Longitudinal follow-up of biochemical markers of fatigue throughout a sporting season in young elite rugby players	Journal of Strength and Conditioning Research	No	Before January1,2014

	Duclos					
362	A. Ali; C. Williams; M. Hulse; A. Strudwick; J. Reddin; L. Howarth; J. Eldred; M. Hirst; S. McGregor	2007	Reliability and validity of two tests of soccer skill	Journal of Sports Sciences	No	Before January1,2014
363	M. S. Allen; M. Jones; P. J. McCarthy; S. Sheehan-Mansfield; D. Sheffield	2013	Emotions correlate with perceived mental effort and concentration disruption in adult sport performers	European Journal of Sport Science	No	Before January1,2014
364	M. T. Allison; C. Meyer	1988	Career Problems and Retirement Among Elite Athletes: The Female Tennis Professional	Sociology of Sport Journal	No	Before January1,2014
365	A. J. Amorose; P. J. K. Smith	2003	Feedback as a Source of Physical Information: Effects of Age, Experience and Type of Feedback	Journal of Sport & Exercise Psychology	No	Before January1,2014
366	K. Anderson	2013	A CHANGE OF MIND	Sports Illustrated	No	Before January1,2014
367	M. Arkush	1996	Pain reliever	Golf World	No	Before January1,2014
368	M. Audiffren; P. D. Tomporowski; J. Zagrodnik	2009	Acute aerobic exercise and information processing: Modulation of executive control in a Random Number Generation task	Acta Psychologica	No	Before January1,2014
369	M. Bamberger	2002	The Amazing ANNIKA	Sports Illustrated	No	Before January1,2014
370	N. Best	2001	New York Giants	Sporting News	No	Before January1,2014
371	B. K. Britton; A. Tesser	1982	Effects of prior knowledge on use of cognitive capacity in three complex cognitive tasks	Journal of Verbal Learning and Verbal Behavior	No	Before January1,2014
372	C. N. Brown; K. M. Guskiewicz; J. Bleiberg	2007	Athlete Characteristics and Outcome Scores for Computerized Neuropsychological Assessment: A Preliminary Analysis	Journal of Athletic Training (National Athletic Trainers' Association)	No	Before January1,2014
373	M. Buchheit; C. Horobeanu; A. Mendez-Villanueva; B. M. Simpson; P. C. Bourdon	2011	Effects of age and spa treatment on match running performance over two consecutive games in highly trained young soccer players	Journal of Sports Sciences	No	Before January1,2014
374	R. M. Buscombe; I. A. Greenlees	2012	The role of time pressure and accountability in moderating the impact of expectancies on judgments of tennis performance	International Journal of Sport Psychology	No	Before January1,2014
375	R. Cañal-Bruland; J. R. Pijpers; R. D. Oudejans	2010	The influence of anxiety on action-specific perception	Anxiety, Stress & Coping	No	Before January1,2014
376	L. O. Carneiro Rodrigues; F. de Castro Magalhães	2004	Car racing: In the heat of competition	Revista Brasileira de Medicina do Esporte	No	Before January1,2014
377	C. M. Cassidy; D. E. Conroy	2006	Children's Self-Esteem Related to School- and Sport-Specific Perceptions of Self and Others	Journal of Sport Behavior	No	Before January1,2014
378	A. Cooke; M. Kavussanu; D. McIntyre; I. D. Boardley; C. Ring	2011	Effects of competitive pressure on expert performance: Underlying psychological, physiological, and kinematic mechanisms	Psychophysiology	No	Before January1,2014
379	A. Cooke; M. Kavussanu; D. McIntyre; C. Ring	2010	Psychological, muscular and kinematic factors mediate performance under pressure	Psychophysiology	No	Before January1,2014
380	S. L. Cresswell; R. C. Eklund	2006	Changes in athlete burnout over a thirty-week rugby year""	Journal of Science and Medicine in Sport	No	Before January1,2014
381	L. Crognier; Y. A. Féry	2007	40 years of research on anticipation in tennis: A critical review	Science et Motricite	No	Before January1,2014
382	B. Cunniffe; A. J. Hore; D.	2010	Time course of changes in	European Journal of Applied	No	Before

	M. Whitcombe; K. P. Jones; J. S. Baker; B. Davies		immuneoendocrine markers following an international rugby game	Physiology		January1,2014
383	B. Cunniffe; A. J. Hore; D. M. Whitcombe; K. P. Jones; B. Davies; J. S. Baker	2011	Immunoendocrine responses over a three week international rugby union series	Journal of Sports Medicine and Physical Fitness	No	Before January1,2014
384	D. de la Peña; N. Murray; C. Janelle	2008	Implicit overcompensation: The influence of negative self-instructions on performance of a self-paced motor task	Journal of Sports Sciences	No	Before January1,2014
385	A. Delextrat; J. Calleja-González; A. Hippocrate; N. D. Clarke	2013	Effects of sports massage and intermittent cold-water immersion on recovery from matches by basketball players	Journal of Sports Sciences	No	Before January1,2014
386	N. Dlodlo; M. Dhurup	2013	Are university students living in a world of fantasy? Fantasy football motives among students at a South African university of technology	African Journal for Physical, Health Education, Recreation & Dance	No	Before January1,2014
387	D. C. Dorris; D. A. Power; E. Kenefick	2012	Investigating the effects of ego depletion on physical exercise routines of athletes	PSYCHOLOGY OF SPORT AND EXERCISE	No	Before January1,2014
388	R. Duffield; A. Coutts; A. McCall; D. Burgess	2013	Pre-cooling for football training and competition in hot and humid conditions	European Journal of Sport Science	No	Before January1,2014
389	M. Duncan; S. Taylor; M. Lyons	2012	The Effect of Caffeine Ingestion on Field Hockey Skill Performance Following Physical Fatigue	Research in Sports Medicine	No	Before January1,2014
390	B. Dwyer	2011	The Impact of Fantasy Football Involvement on Intentions to Watch National Football League Games on Television	International Journal of Sport Communication	No	Before January1,2014
391	C. Englert; A. Bertrams	2012	Anxiety, Ego Depletion, and Sports Performance	Journal of Sport & Exercise Psychology	No	Before January1,2014
392	B. Ermeling	2012	Improving Teaching through Continuous Learning: The Inquiry Process John Wooden Used to Become Coach of the Century	Quest (00336297)	No	Before January1,2014
393	K. Eun Chang	2005	The Immediate Effects of Various Task Presentation Types on Middle School Students' Skill Learning	International Journal of Applied Sports Sciences	No	Before January1,2014
394	M. Farber	1998	Peter the great	Sports Illustrated	No	Before January1,2014
395	D. Farrow; D. Pyne; T. Gabbett	2008	Skill and Physiological Demands of Open and Closed Training Drills in Australian Football	International Journal of Sports Science & Coaching	No	Before January1,2014
396	T. Finkenzeller; M. Doppelmayr; G. Amesberger	2012	Heart rate variability as an indicator of attention focus in golfers of differing performance levels	Zeitschrift fur Sportpsychologie	No	Before January1,2014
397	J. Finn	2010	Leading Article: Discovering Golf's Innermost Truths: A New Approach to Teaching the Game	International Journal of Sports Science & Coaching	No	Before January1,2014
398	G. S. Fleisig; A. Weber; N. Hassell; J. R. Andrews	2009	Prevention of Elbow Injuries in Youth Baseball Pitchers	Current Sports Medicine Reports (American College of Sports Medicine)	No	Before January1,2014
399	A. Follenfant; J. B. Légal; F. M. Dit Dinard; T. Meyer	2005	Effect of stereotypes activation on behavior: An application in a sport setting	Revue europeenne de psychologie appliquee	No	Before January1,2014
400	A. Frank; M. Belokopytov; D. Moran; Y. Shapiro; Y. Epstein	2001	Changes In Heart Rate Variability Following Acclimation To Heat	Journal of Basic and Clinical Physiology and Pharmacology	No	Before January1,2014
401	P. Furley; A. Bertrams; C. Englert; A. Delphia	2013	Ego depletion, attentional control, and decision making in sport	PSYCHOLOGY OF SPORT AND EXERCISE	No	Before January1,2014

402	T. Gabbett; D. Jenkins; B. Abernethy	2009	Game-Based Training for Improving Skill and Physical Fitness in Team Sport Athletes	INTERNATIONAL JOURNAL OF SPORTS SCIENCE & COACHING	No	Before January1,2014
403	T. Gabbett; M. Wake; B. Abernethy	2011	Use of dual-task methodology for skill assessment and development: Examples from rugby league	Journal of Sports Sciences	No	Before January1,2014
404	T. J. Gabbett; D. G. Jenkins; B. Abernethy	2010	Physiological and skill demands of 'on-side' and 'off-side' games	Journal of Strength and Conditioning Research	No	Before January1,2014
405	P. Gaudreau; A. Nicholls; A. R. Levy	2010	The Ups and Downs of Coping and Sport Achievement: An Episodic Process Analysis of Within-Person Associations	Journal of Sport & Exercise Psychology	No	Before January1,2014
406	A. Gil; M. Perla Moreno; L. GarcÍA-GonzÁLez; A. Moreno; F. Del Villar	2012	ANALYSIS OF DECLARATIVE AND PROCEDURAL KNOWLEDGE IN VOLLEYBALL ACCORDING TO THE LEVEL OF PRACTICE AND PLAYERS' AGE	Perceptual & Motor Skills	No	Before January1,2014
407	R. V. Gomes; A. J. Coutts; L. Viveiros; M. S. Aoki	2011	Physiological demands of match-play in elite tennis: A case study	European Journal of Sport Science	No	Before January1,2014
408	A. M. Gorce-Dupuy; C. Vela; S. Badiou; A. S. Bargnoux; C. Josse; N. Roagna; M. Delage; F. Michel; M. H. Vernet; D. Destizons; J. P. Cristol	2012	Antioxidant and oligonutrient status, distribution of amino acids, muscle damage, inflammation, and evaluation of renal function in elite rugby players	Clinical Chemistry and Laboratory Medicine	No	Before January1,2014
409	K. C. Graham; K. E. French; A. M. Woods	1993	Observing and Interpreting Teaching-Learning Processes: Novice PETE Students, Experienced PETE Students, and Expert Teacher Educators	Journal of Teaching in Physical Education	No	Before January1,2014
410	M. Gregg; C. Hall	2006	The Relationship of Skill Level and Age to the Use of Imagery by Golfers	Journal of Applied Sport Psychology	No	Before January1,2014
411	A. Guillot; C. Collet	2005	Duration of Mentally Simulated Movement: A Review	Journal of Motor Behavior	No	Before January1,2014
412	A. Guillot; C. Collet; A. Dittmar	2004	Relationship between visual and kinesthetic imagery, field dependence-independence, and complex motors skills	Journal of Psychophysiology	No	Before January1,2014
413	A. Guillot; C. Collet; A. Dittmar	2005	Influence of environmental context on motor imagery quality: An autonomic nervous system study	Biology of Sport	No	Before January1,2014
414	K. M. Guskiewicz; S. L. Bruce; R. C. Cantu; M. S. Ferrara; J. P. Kelly; M. McCrea; M. Putukian; T. C. Valovich McLeod	2004	National Athletic Trainers' Association Position Statement: Management of Sport-Related Concussion	Journal of Athletic Training (National Athletic Trainers' Association)	No	Before January1,2014
415	J. L. Gusthart; I. M. Kelly; J. E. Rink	1997	The validity of the qualitative measures of teaching performance scale as a measure of teacher effectiveness	Journal of Teaching in Physical Education	No	Before January1,2014
416	R. Halvorson	2009	PE-RELATED INJURIES ON THE RISE	IDEA Fitness Journal	No	Before January1,2014
417	J. Hawkins	2006	The Lost Generation	Golf World	No	Before January1,2014
418	J. Hawkins	2006	What Should Phil do next?	Golf World	No	Before January1,2014
419	R. Hayman; R. Polman; E. Borkoles; J. Taylor	2013	The Influence of a Deliberate Practice Intervention on the Putting Performance and Subsequent Practice Behaviours of Aspiring Elite Adolescent Golfers	Talent Development & Excellence	No	Before January1,2014
420	R. Hayman; R. Polman; J. Taylor	2012	The validity of retrospective recall in assessing practice regimes in golf	International Journal of Sport & Exercise Psychology	No	Before January1,2014

421	R. Hayman; R. Polman; J. Taylor; B. Hemmings; E. Borkoles	2011	Development of Elite Adolescent Golfers	Talent Development & Excellence	No	Before January1,2014
422	D. L. Herbert	2010	New NFL, Professional Association and State Actions on Concussions	Sports, Parks & Recreation Law Reporter	No	Before January1,2014
423	R. Herrington	2005	Led by captain Kevin Kisner's return to form, consistent Georgia plays to its potential and captures the NCAAs	Golf World	No	Before January1,2014
424	D. Higdon	1997	Grass court crash course	Tennis	No	Before January1,2014
425	T. R. Higgins; I. T. Heazlewood; M. Climstein	2011	A random control trial of contrast baths and ICE baths for recovery during competition in U/20 rugby union	Journal of Strength and Conditioning Research	No	Before January1,2014
426	G. R. Hockey; P. Nickel; A. C. Roberts; M. H. Roberts	2009	Sensitivity of candidate markers of psychophysiological strain to cyclical changes in manual control load during simulated process control	Appl Ergon	No	Before January1,2014
427	G. R. Hockey; J. Sauer	1996	Cognitive fatigue and complex decision making under prolonged isolation and confinement	Adv Space Biol Med	No	Before January1,2014
428	G. R. Hockey; D. G. Wastell; J. Sauer	1998	Effects of sleep deprivation and user interface on complex performance: a multilevel analysis of compensatory control	Hum Factors	No	Before January1,2014
429	G. R. Hockey; M. Wiethoff	1993	European isolation and confinement study. Cognitive fatigue in complex decision-making	Adv Space Biol Med	No	Before January1,2014
430	G. R. J. Hockey; F. Earle	2006	Control over the scheduling of simulated office work reduces the impact of workload on mental fatigue and task performance	JOURNAL OF EXPERIMENTAL PSYCHOLOGY-APPLIED	No	Before January1,2014
431	L. Houghton; B. Dawson; J. Rubenson; M. Tobin	2011	Movement patterns and physical strain during a novel, simulated cricket batting innings (BATEX)	Journal of Sports Sciences	No	Before January1,2014
432	T. N. Hunt; M. S. Ferrara	2009	Age-Related Differences in Neuropsychological Testing Among High School Athletes	Journal of Athletic Training (National Athletic Trainers' Association)	No	Before January1,2014
433	C. Ioan Teodor; M. Claudiu	2012	EFFECT OF PHYSICAL AND TECHNICAL TRAINING ON THE SHARE OF INDIVIDUAL AND COLLECTIVE TACTICAL ACTIONS, IN A OFFICIAL GAME OF HANDBALL, AT THE JUNIORS TWO (JUN. II)	Ovidius University Annals, Series Physical Education & Sport/Science, Movement & Health	No	Before January1,2014
434	M. Isabel; S. António; R. António; P. Felismina; M. Michel	2008	A Systematic Observation of Youth Amateur Volleyball Coathes Behaviours	International Journal of Applied Sports Sciences	No	Before January1,2014
435	J. C. Ives; G. A. Shelley	2003	Psychophysics in functional strength and power training: Review and implementation framework	JOURNAL OF STRENGTH AND CONDITIONING RESEARCH	No	Before January1,2014
436	P. M. Jakeman; E. M. Winter; J. Doust	1994	A review of research in sports physiology	Journal of Sports Sciences	No	Before January1,2014
437	R. D. Johnston; T. J. Gabbett; D. G. Jenkins	2013	Influence of an intensified competition on fatigue and match performance in junior rugby league players	Journal of Science and Medicine in Sport	No	Before January1,2014
438	R. D. Johnston; N. V. Gibson; C. Twist; T. J. Gabbett; S. A. MacNay; N. G. MacFarlane	2013	Physiological responses to an intensified period of rugby league competition	Journal of Strength and Conditioning Research	No	Before January1,2014
439	J. Jooste; J. G. U. Van Wyk; B. J. M. Steyn	2013	The relationship between mental skills and level of cricket participation	African Journal for Physical, Health Education, Recreation	No	Before January1,2014

				& Dance		
440	K. Kaplanidou; H. Gibson	2010	Predicting Behavioral Intentions of Active Event Sport Tourists: The Case of a Small-scale Recurring Sports Event	Journal of Sport & Tourism	No	Before January1,2014
441	R. T. Kellogg	2001	Long-term working memory in text production	Memory and Cognition	No	Before January1,2014
442	T. H. Kim; A. Cruz	2011	Differences in brain activation during motor imagery and action observation of golf putting	Scientific Research and Essays	No	Before January1,2014
443	T. H. Kim; A. Cruz; J. H. Ha	2011	Differences in learning facilitatory effect of Motor Imagery and Action Observation of golf putting	Journal of Applied Sciences	No	Before January1,2014
444	M. J. Klusemann; D. B. Pyne; W. G. Hopkins; E. J. Drinkwater	2013	Activity Profiles and Demands of Seasonal and Tournament Basketball Competition	International Journal of Sports Physiology & Performance	No	Before January1,2014
445	A. P. Kontos; R. J. Elbin; B. Lau; S. Simensky; B. Freund; J. French; M. W. Collins	2013	Posttraumatic Migraine as a Predictor of Recovery and Cognitive Impairment After Sport-Related Concussion	American Journal of Sports Medicine	No	Before January1,2014
446	A. Koustelios	2010	Burnout among football coaches in Greece	Biology of Exercise	No	Before January1,2014
447	S. Laborde; M. Raab	2013	The Tale of Hearts and Reason: The Influence of Mood on Decision Making	Journal of Sport & Exercise Psychology	No	Before January1,2014
448	W. K. Lam; R. S. W. Masters; J. P. Maxwell	2010	Cognitive demands of error processing associated with preparation and execution of a motor skill	CONSCIOUSNESS AND COGNITION	No	Before January1,2014
449	F. M. Lichacz; J. T. Partington	1996	Collective efficacy and true group performance	International Journal of Sport Psychology	No	Before January1,2014
450	R. Lidor	2004	Developing metacognitive behaviour in physical education classes:the use of task-pertinent learning strategies	Physical Education & Sport Pedagogy	No	Before January1,2014
451	Z. Lin; J. Yao	2012	Application of factor analysis and pareto analysis for the value of college students regarding playing basketball	International Journal of Digital Content Technology and its Applications	No	Before January1,2014
452	C. Liu; K. Conn; N. Sarkar; W. Stone	2008	Online affect detection and robot behavior adaptation for intervention of children with autism	IEEE Transactions on Robotics	No	Before January1,2014
453	J. Low; A. M. Williams; A. P. McRobert; P. R. Ford	2013	The microstructure of practice activities engaged in by elite and recreational youth cricket players	Journal of Sports Sciences	No	Before January1,2014
454	C. Lundqvist; G. Kenttä	2010	Positive emotions are not simply the absence of the negative ones: Development and validation of the emotional recovery questionnaire (EmRecQ)	Sport Psychologist	No	Before January1,2014
455	M. Lyons; Y. Al-Nakeeb; J. Hankey; A. Nevill	2013	The effect of moderate and high-intensity fatigue on groundstroke accuracy in expert and non-expert tennis players	Journal of Sports Science and Medicine	No	Before January1,2014
456	W. Machnacz; A. Dudkowski; A. Rokita; Ł. Błach	2013	Measurement of reaction time in groups of young athletes in the conditions which require focused attention	Journal of Combat Sports & Martial Arts	No	Before January1,2014
457	C. MacMahon; W. Helsen; J. Starkes; M. Weston	2007	Decision-making skills and deliberate practice in elite association football referees	Journal of Sports Sciences	No	Before January1,2014
458	C. MacMahon; J. Starkes	2008	Contextual influences on baseball ball-strike decisions in umpires, players, and controls	Journal of Sports Sciences	No	Before January1,2014
459	J. Mallo; P. Frutos; D. Juárez; E. Navarro	2012	Effect of positioning on the accuracy of decision making of association football top-class referees and assistant referees	Journal of Sports Sciences	No	Before January1,2014

			during competitive matches			
460	J. G. Martínez; A. C. Bonet; F. L. Encinas	1998	Psychological programe to improve the output of handball players	Psicothema	No	Before January1,2014
461	T. Mashiko; T. Umeda; S. Nakaji; K. Sugawara	2004	Position related analysis of the appearance of and relationship between post-match physical and mental fatigue in university rugby football players	British Journal of Sports Medicine	No	Before January1,2014
462	S. Mellalieu; G. Trewartha; K. Stokes	2008	Science and rugby union	Journal of Sports Sciences	No	Before January1,2014
463	D. Memmert; S. Geppert; D. Seiler; N. Hagemann; R. Althoetmar	2009	Conditions of practice in perceptual skill learning	Research Quarterly for Exercise and Sport	No	Before January1,2014
464	D. Middleton	2012	ROUND 2 PREVIEWS	Rugby League Week	No	Before January1,2014
465	J. Milton; A. Solodkin; P. Hluštík; S. L. Small	2007	The mind of expert motor performance is cool and focused	NeuroImage	No	Before January1,2014
466	M. B. Mousseau; C. M. Janelle; S. A. Coombes; D. T. Y. Mann	2005	Cognitive fatigue and hockey decision-making: A multimethod exploration	JOURNAL OF SPORT & EXERCISE PSYCHOLOGY	No	Before January1,2014
467	D. Mroczek; A. Kawczyński; E. Superlak; J. Chmura	2013	Psychomotor performance of elite volleyball players during a game	Perceptual and Motor Skills	No	Before January1,2014
468	R. Mullen; L. Hardy; A. Tattersall	2005	The Effects of Anxiety on Motor Performance: A Test of the Conscious Processing Hypothesis	Journal of Sport & Exercise Psychology	No	Before January1,2014
469	M. Nédélec; A. McCall; C. Carling; F. Legall; S. Berthoin; G. Dupont	2012	Recovery in Soccer Part I - Post-Match Fatigue and Time Course of Recovery	SPORTS MEDICINE	No	Before January1,2014
470	M. Nédélec; A. McCall; C. Carling; F. Legall; S. Berthoin; G. Dupont	2013	Recovery in Soccer Part II-Recovery Strategies	SPORTS MEDICINE	No	Before January1,2014
471	M. Nedelec; U. Wisloff; A. McCall; S. Berthoin; G. Dupont	2013	Recovery after an intermittent test	International Journal of Sports Medicine	No	Before January1,2014
472	A. M. Nevill; N. J. Balmer; A. Mark Williams	2002	The influence of crowd noise and experience upon refereeing decisions in football	Psychology of Sport and Exercise	No	Before January1,2014
473	N. Nibbeling; R. R. D. Oudejans; H. A. M. Daanen	2012	Effects of anxiety, a cognitive secondary task, and expertise on gaze behavior and performance in a far aiming task	PSYCHOLOGY OF SPORT AND EXERCISE	No	Before January1,2014
474	T. A. C. Oliveira; R. A. Denardi; G. Tani; U. C. Corrêa	2013	Effects of Internal and External Attentional Foci on Motor Skill Learning: Testing the Automation Hypothesis	Human Movement	No	Before January1,2014
475	F. Pereira; I. Mesquita; A. Graça	2009	Accountability systems and instructional approaches in youth volleyball training	Journal of Sports Science & Medicine	No	Before January1,2014
476	F. Pereira Marques; I. Mesquita Ribeiro; A. Graça Santos; M. P. Moreno Arroyo	2010	Multidimensional analysis of pedagogical feedback in volleyball training setting	Revista Internacional de Medicina y Ciencias de la Actividad Fisica y del Deporte	No	Before January1,2014
477	G. Pezzulo; D. Ognibene	2012	Proactive Action Preparation: Seeing Action Preparation as a Continuous and Proactive Process	Motor Control	No	Before January1,2014
478	A. Piras; R. Lobietti; S. Squatrito	2010	A study of saccadic eye movement dynamics in volleyball: Comparison between athletes and non-athletes	Journal of Sports Medicine and Physical Fitness	No	Before January1,2014
479	R. Pollard	1986	Home advantage in soccer: A retrospective analysis	Journal of Sports Sciences	No	Before January1,2014
480	R. Pollard	2002	Evidence of a reduced home advantage when	Journal of Sports Sciences	No	Before

			a team moves to a new stadium			January1,2014
481	J. Price; D. L. Gill; J. Etnier; K. Kornatz	2009	Free-throw shooting during dual-task performance: Implications for attentional demand and performance	Research Quarterly for Exercise and Sport	No	Before January1,2014
482	M. Rendell; R. W. Masters; D. Farrow; T. Morris	2011	An Implicit Basis for the Retention Benefits of Random Practice	Journal of Motor Behavior	No	Before January1,2014
483	P. Richter; T. Wagner; R. Heger; G. Weise	1998	Psychophysiological analysis of mental load during driving on rural roads - A quasi-experimental field study	Ergonomics	No	Before January1,2014
484	S. P. Roberts; K. A. Stokes; G. Trewartha; P. Hogben; J. Doyle; D. Thompson	2011	Effect of combined carbohydrate-protein ingestion on markers of recovery after simulated rugby union match-play	Journal of Sports Sciences	No	Before January1,2014
485	R. Roczniok; A. Maszczyk; A. Stanula; M. Czuba; P. Pietraszewski; J. Kantyka; M. Starzyński	2013	Physiological and physical profiles and on-ice performance approach to predict talent in male youth ice hockey players during draft to hockey team	Isokinetics & Exercise Science	No	Before January1,2014
486	R. Roure; C. Collet; C. Deschaumes-Molinaro; G. Delhomme; A. Dittmar; E. Vernet-Maury	1999	Imagery quality estimated by autonomic response is correlated to sporting performance enhancement	Physiology and Behavior	No	Before January1,2014
487	R. Roure; C. Collet; C. Deschaumes-Molinaro; A. Dittmar; H. Rada; G. Delhomme; E. Vernet-Maury	1998	Autonomic nervous system responses correlate with mental rehearsal in volleyball training	European Journal of Applied Physiology and Occupational Physiology	No	Before January1,2014
488	M. Roy; J. P. Brunelle; G. Perreault; J. F. Desbiens; C. Spallanzani; S. Turcotte	2006	Implementation of a decision training program with a varsity canadian football team	Staps	No	Before January1,2014
489	K. Royal; D. Farrow; I. Mujika; S. Halson; D. Pyne; B. Abernethy	2006	The effects of fatigue on decision making and shooting skill performance in water polo players	Journal of Sports Sciences	No	Before January1,2014
490	D. Sacha; L. Simmering; M. Adler	2012	A Birdie in the Hand: Asymmetry in Golf Risk Preferences	International Journal of Golf Science	No	Before January1,2014
491	J. Sauer; D. G. Wastell; G. R. Hockey; F. Earle	2003	Performance in a complex multiple-task environment during a laboratory-based simulation of occasional night work	Hum Factors	No	Before January1,2014
492	J. M. H. Schellekens; G. J. Sijtsma; E. Vegter; T. F. Meijman	2000	Immediate and delayed after-effects of long lasting mentally demanding work	BIOLOGICAL PSYCHOLOGY	No	Before January1,2014
493	K. Schluter	2010	TO Snack OR NOT TO Snack...: WHY IS THAT EVEN A QUESTION?	Volleyball (10584668)	No	Before January1,2014
494	B. Schmand; T. Kuipers; M. G. Van Der; J. Bosveld; F. Bulthuis; M. Jellema	1994	Cognitive Disorders and Negative Symptoms as Correlates of Motivational Deficits in Psychotic Patients	Psychological Medicine	No	Before January1,2014
495	J. Schorer; S. Cobley; D. Büsch; H. Bräutigam; J. Baker	2009	Influences of competition level, gender, player nationality, career stage and playing position on relative age effects	Scandinavian Journal of Medicine & Science in Sports	No	Before January1,2014
496	K. Shih-Chun; H. Chung-Ju; H. Tsung-Min	2013	Frontal Midline Theta is a Specific Indicator of Optimal Attentional Engagement During Skilled Putting Performance	Journal of Sport & Exercise Psychology	No	Before January1,2014
497	B. A. Sibley; J. L. Etnier	2004	Time course of attention and decision making during a volleyball set	Research Quarterly for Exercise and Sport	No	Before January1,2014
498	C. C. Silva; T. B. L. Goldberg; R. C. Capela; C. S. Kurokawa; A. D. S.	2007	Acute post-exercise blood lactate and creatin phosphokinase levels responses in young athletes	Revista Brasileira de Medicina do Esporte	No	Before January1,2014

	Teixeira; J. C. Dalmas; E. S. Cyrino					
499	M. Silver	1997	Sound and fury	Sports Illustrated	No	Before January1,2014
500	J. Siniscalchi; C. D. Pierskalla	2005	FLYING SAUCERS	Parks & Recreation	No	Before January1,2014
501	N. C. Smith; M. Bellamy; D. J. Collins; D. Newell	2001	A test of processing efficiency theory in a team sport context	Journal of Sports Sciences	No	Before January1,2014
502	E. J. Stevenson; P. R. Hayes; S. J. Allison	2009	The effect of a carbohydrate-caffeine sports drink on simulated golf performance	Applied Physiology, Nutrition & Metabolism	No	Before January1,2014
503	M. Suzuki; T. Umeda; S. Nakaji; T. Shimoyama; T. Mashiko; K. Sugawara	2004	Effect of incorporating low intensity exercise into the recovery period after a rugby match	Br J Sports Med	No	Before January1,2014
504	A. J. Tattersall; G. R. J. Hockey	1995	Level of operator control and changes in heart rate variability during simulated flight maintenance	HUMAN FACTORS	No	Before January1,2014
505	Y. Tsuji; G. Bennett; J. H. Leigh	2009	Investigating Factors Affecting Brand Awareness of Virtual Advertising	Journal of Sport Management	No	Before January1,2014
506	C. Twist; M. Waldron; J. Highton; D. Burt; M. Daniels	2012	Neuromuscular, biochemical and perceptual post-match fatigue in professional rugby league forwards and backs	Journal of Sports Sciences	No	Before January1,2014
507	J. N. Vickers; L. F. Livingston; S. Umeris-Bohnert; D. Holden	1999	Decision training: The effects of complex instruction, variable practice and reduced delayed feedback on the acquisition and transfer of a motor skill	Journal of Sports Sciences	No	Before January1,2014
508	F. Vohle	2009	Cognitive Tools 2.0 in Trainer Education	International Journal of Sports Science & Coaching	No	Before January1,2014
509	A. M. Williams; J. Vickers; S. Rodrigues	2002	The Effects of Anxiety on Visual Search, Movement Kinematics, and Performance in Table Tennis: A Test of Eysenck and Calvo's Processing Efficiency Theory	Journal of Sport & Exercise Psychology	No	Before January1,2014
510	A. M. Williams; P. Ward; J. M. Knowles; N. J. Smeeton	2002	Anticipation skill in a real-world task: Measurement, training, and transfer in tennis	Journal of Experimental Psychology: Applied	No	Before January1,2014
511	M. Wilson; N. C. Smith	2007	A test of the predictions of processing efficiency theory during elite team competition using the Thought Occurrence Questionnaire for Sport	INTERNATIONAL JOURNAL OF SPORT PSYCHOLOGY	No	Before January1,2014
512	M. Wilson; N. C. Smith; P. S. Holmes	2007	The role of effort in influencing the effect of anxiety on performance: Testing the conflicting predictions of processing efficiency theory and the conscious processing hypothesis	British Journal of Psychology	No	Before January1,2014
513	L. Wing Kai; J. P. Maxwell; R. Masters	2009	Analogy Learning and the Performance Motor Skills Under Pressure	Journal of Sport & Exercise Psychology	No	Before January1,2014
514	J. M. Wood; D. L. Feltz	2013	Preparatory efficacy effects on practice effort and performance	Open Sports Sciences Journal	No	Before January1,2014
515	T. Woodman; P. A. Davis	2008	The Role of Repression in the Incidence of Ironic Errors	Sport Psychologist	No	Before January1,2014
516	M. J. Wright; D. T. Bishop; R. C. Jackson; B. Abernethy	2013	Brain regions concerned with the identification of deceptive soccer moves by higher-skilled and lower-skilled players	Front Hum Neurosci	No	Before January1,2014
517		2000	CHAPTER 2: Putting Pressure Into Practices		No	Book
518	A. J. H. Arundale; M. Bryant; L. Gartner	2020	On-Court Rehabilitation-From Treatment Table and Return to Play to a Return to Performance	Basketball Sports Medicine and Science	No	Book
519	D. F. Bjorklund	2018	Memory, strategies, knowledge, and	Conceptions of Development:	No	Book

			evolution: The evolution of a developmental	Lessons from the Laboratory		
520	R. W. Christina; E. Alpenfels	2012	Why does traditional training fail to optimize playing performance?	Science and Golf IV: Proceedings of the World Scientific Congress of Golf	No	Book
521	A. Eliakim; D. Nemet	2020	Endocrine Aspects in Performance and Recovery in Basketball	Basketball Sports Medicine and Science	No	Book
522	D. Farrow; T. Buszard	2017	Exploring the applicability of the contextual interference effect in sports practice	SPORT AND THE BRAIN: THE SCIENCE OF PREPARING, ENDURING AND WINNING, PT B	No	Book
523	C. F. Finch; A. Williamson; B. O'Brien	2011	An overview of the epidemiological evidence linking injury risk to fatigue in sport: Identification of research needs and opportunities	Regulation of Fatigue in Exercise	No	Book
524	A. P. Friesen; T. J. Devonport; A. M. Lane; C. N. Sellars	2017	Interpersonal emotion regulation: An intervention case study with a professional ice hockey team	Research in Sport Psychology	No	Book
525	J. Heidari; F. Loch; M. Kellmann	2019	Recovery in football	Football Psychology: From Theory to Practice	No	Book
526	R. Hockey	2011	The psychology of fatigue: Work, effort and control	The Psychology of Fatigue: Work, Effort and Control	No	Book
527	R. Hockey	2013	Psychology of Fatigue: Work, Effort and Control	PSYCHOLOGY OF FATIGUE: WORK, EFFORT AND CONTROL	No	Book
528	T. Huyghe; J. Calleja-Gonzalez; N. Terrados	2020	Post-Exercise Recovery Strategies in Basketball: Practical Applications Based on Scientific Evidence	Basketball Sports Medicine and Science	No	Book
529	B. Johnson; E. D. Bigler; S. Slobounov	2019	Functional Neuroimaging Markers of Persistent Post-Concussive Brain Change	Concussion and Traumatic Encephalopathy: Causes, Diagnosis, and Management	No	Book
530	M. I. Kawas; C. A. Sheridan; W. C. Flood; A. P. Sweeney; C. T. Whitlow	2023	TBI Sports Related Injury	Functional Neuroradiology: Principles and Clinical Applications	No	Book
531	T. Meyer; R. Julian; C. Thompson	2018	Exercise physiology of football: Factors related to performance and health	Return to Play in Football: An Evidence-based Approach	No	Book
532	M. Shacklock	2005	Clinical Neurodynamics	Clinical Neurodynamics	No	Book
533	J. Shapiro; J. Aronson; M. S. McGlone	2015	Stereotype threat	Handbook of Prejudice, Stereotyping, and Discrimination: 2nd Edition	No	Book
534	K. Slattery; A. J. Coutts	2019	The Application of Heat Stress to Team Sports: Football/Soccer, Australian Football and Rugby	Heat Stress in Sport and Exercise: Thermophysiology of Health and Performance	No	Book
535	S. M. Slobounov; W. J. Sebastianelli; K. M. Newell	2014	Feasibility of virtual reality for assessment of neurocognitive, executive, and motor functions in concussion	Concussions in Athletics: From Brain to Behavior	No	Book
536	Y. Takahashi	2015	Anserine as a suppressor of fatigue	Food and Nutritional Components in Focus	No	Book
537	M. R. Wilson; A. Webb; L. J. Wylie; S. J. Vine	2018	The quiet eye is sensitive to exercise-induced physiological stress	Progress in Brain Research	No	Book
538	J. J. Cañas	2017	Fatigue and Theories of Resources: The Energetic Metaphor of the Mind Functioning Might Be Reconsidered	ADVANCES IN NEUROERGONOMICS AND COGNITIVE ENGINEERING	No	Conference
539	M. Cavus; P. Biecek	2022	Explainable expected goal models for performance analysis in football analytics	Proceedings - 2022 IEEE 9th International Conference on Data Science and Advanced Analytics, DSAA 2022	No	Conference

540	Q. Guo; J. Wu; B. Li	2015	EEG-based golf putt outcome prediction using support vector machine	IEEE SSCI 2014 - 2014 IEEE Symposium Series on Computational Intelligence - CIBCI 2014: 2014 IEEE Symposium on Computational Intelligence in Brain Computer Interfaces, Proceedings	No	Conference
541	M. E. Hamlaoui; M. A. Lahsaini; S. H. Moselhy; T. Fissaa; Y. Laghouaouta	2023	Basketball Free Throw Analysis using Object Detection Techniques	Proceedings - 10th International Conference on Wireless Networks and Mobile Communications, WINCOM 2023	No	Conference
542	G. R. J. Hockey; R. L. Payne; J. T. Rick	1996	Intra-individual patterns of hormonal and affective adaptation to work demands: An n = 2 study of junior doctors	Biological Psychology	No	Conference
543	W. G. Hopkins	2021	Athlete Monitoring and Much More at the Virtual 26th Annual Meeting of the European College of Sport Science	Sportscience	No	Conference
544	M. Igorov; R. Predoiu; A. Predoiu; A. Igorov	2016	Creativity, Resistance to Mental Fatigue and Coping Strategies in Junior Women Handball Players	ICPESK 2015 - 5TH INTERNATIONAL CONGRESS ON PHYSICAL EDUCATION, SPORT AND KINETOTHERAPY	No	Conference
545	A. Kostenko; P. Rauffet; S. Moga; G. Coppin	2019	Operator Functional State: Measure It with Attention Intensity and Selectivity, Explain It with Cognitive Control	Communications in Computer and Information Science	No	Conference
546	P. Lenik; T. Krzeszowski; K. Przednowek; J. Lenik	2015	The analysis of basketball free throw trajectory using PSO algorithm	icSPORTS 2015 - Proceedings of the 3rd International Congress on Sport Sciences Research and Technology Support	No	Conference
547	K. J. Y. Lim; K. M. Kee	2023	Relationship of Personality Traits and Competitive Trait Anxiety in Recreational Individual Event Sports	Lecture Notes in Bioengineering	No	Conference
548	M. I. A. Manaf; N. I. Mohamad; N. F. A. Malek; A. A. M. Jahizi; A. M. Nadzalan	2021	The Relationship between Ball Speed and Shooting Accuracy during Field Hockey Hit	Journal of Physics: Conference Series	No	Conference
549	A. P. S. Mohamed Tanveejul; R. Vishakh Maheswar; A. Vikraman; R. D. Dhinesh; S. P. Preejith; M. Sivaprakasam	2021	Shot-wise Heart Rate Variability Analysis on Cricket Players	SeGAH 2021 - 2021 IEEE 9th International Conference on Serious Games and Applications for Health	No	Conference
550	T. J. Roy; M. A. Mahmood; D. Roy	2021	An Efficient Approach to Identify the Key Factors of Failure of Bangladesh Cricket Team in Test Cricket Utilizing Hypothesis Testing and Clustering Method	2021 5th International Conference on Electrical Information and Communication Technology, EICT 2021	No	Conference
551	N. Ruiz; G. Liu; B. Yin; D. Farrow; F. Chen	2010	Teaching athletes cognitive skills: Detecting cognitive load in speech input	Proceedings of the 2010 British Computer Society Conference on Human-Computer Interaction, BCS-HCI 2010	No	Conference
552	C. F. Savu; C. Pehoiu	2018	CORRELATION OF MOTOR AND TECHNICAL TRAINING PARAMETERS FOR JUNIOR I BASKETBALL PLAYERS	ICPESK 2017 - 7TH INTERNATIONAL CONGRESS ON PHYSICAL EDUCATION, SPORT AND KINETOTHERAPY	No	Conference

553	M. V. Stecklow; M. Cagy; A. F. C. Infantosi	2011	Event-related synchronization/desynchronization to assess changes in alpha peak frequency along time during kinesthetic motor imagery	Pan American Health Care Exchanges, PAHCE 2011 - Conference, Workshops, and Exhibits. Cooperation / Linkages: An Independent Forum for Patient Care and Technology Support	No	Conference
554	M. T. Tsai; W. C. Chen; K. M. Chou	2009	The effects of different feedback modes in video practice to improve the decision making of novice officials of ice hockey	Proceedings - International Conference on Management and Service Science, MASS 2009	No	Conference
555	Y. Wang; L. T. Tan; G. Kuan	2023	Tennis Player's Coping Strategies at Duta International Tennis Academy During Their Different Career Phases: A Narrative Review	Lecture Notes in Bioengineering	No	Conference
556	M. Wirth; S. Gradl; D. Poimann; H. Schaefke; J. Matlok; H. Koerger; B. M. Eskofier	2018	Assessment of perceptual-cognitive abilities among athletes in virtual environments: Exploring interaction concepts for soccer players	DIS 2018 - Proceedings of the 2018 Designing Interactive Systems Conference	No	Conference
557	C. Nash; J. Taylor	2021	'Just Let Them Play': Complex Dynamics in Youth Sport, Why It Isn't So Simple	Frontiers in Psychology	No	Abstract and keywords
558	W. Jee; M. Hyun	2023	"10,000 Available" or "10% Remaining": The Impact of Scarcity Framing on Ticket Availability Perceptions in the Secondary Ticket Market	Behavioral Sciences	No	Abstract and keywords
559	N. Goldschmied; M. Raphaeli; E. Morgulev	2023	"Icing the shooter" in basketball: The unintended consequences of time-out management when the game is on the line	Psychology of Sport and Exercise	No	Abstract and keywords
560	J. D. Rooks; A. B. Morrison; M. Goolsarran; S. L. Rogers; A. P. Jha	2017	"We Are Talking About Practice": the Influence of Mindfulness vs. Relaxation Training on Athletes' Attention and Well-Being over High-Demand Intervals	Journal of Cognitive Enhancement	No	Abstract and keywords
561	J. T. Schultz	2019	A CASE FOR RESISTANCE TRAINING IN JUNIOR DEVELOPMENT WITHIN AUSTRALIAN FOOTBALL	Journal of Australian Strength & Conditioning	No	Abstract and keywords
562	D. M. Corbett; A. J. Sweeting; S. Robertson	2019	A change point approach to analysing the match activity profiles of team-sport athletes	Journal of Sports Sciences	No	Abstract and keywords
563	A. C. Field; L. D. Harper; J. W. F. Aldous; R. M. Page	2023	A commentary on soccer match-play simulations for applied research and practice	Science and Medicine in Football	No	Abstract and keywords
564	R. D. Samuel; Y. Galily; O. Guy; E. Sharoni; G. Tenenbaum	2019	A decision-making simulator for soccer referees	International Journal of Sports Science and Coaching	No	Abstract and keywords
565	N. Naser; A. Ali	2016	A descriptive-comparative study of performance characteristics in futsal players of different levels	Journal of Sports Sciences	No	Abstract and keywords
566	L. Baldock; B. Cropley; S. D. Mellalieu; R. Neil	2022	A Longitudinal Examination of Stress and Mental Ill-/Well-Being in Elite Football Coaches	Sport Psychologist	No	Abstract and keywords
567	B. T. Crewther; M. Hecht; N. Potts; L. P. Kilduff; S. Drawer; E. Marshall; C. J. Cook	2020	A longitudinal investigation of bidirectional and time-dependent interrelationships between testosterone and training motivation in an elite rugby environment	Hormones and Behavior	No	Abstract and keywords
568	R. D. Samuel; J. Roy; P. Lew; M. Arnon; G. Tenenbaum	2018	A NEW APPROACH IN EVALUATING ATHLETES' PRE-PERFORMANCE MENTAL STATES IN AN APPLIED CONTEXT	Journal of Contemporary Athletics	No	Abstract and keywords
569	M. A. Carmichael; R. L. Thomson; L. J. Moran; J.	2021	A pilot study on the impact of menstrual cycle phase on elite australian football	International Journal of Environmental Research and	No	Abstract and keywords

	R. Dunstan; M. J. Nelson; M. L. Mathai; T. P. Wycherley		athletes	Public Health		
570	J. Heidari; M. Pelka; J. Beckmann; M. Kellmann	2019	A practitioner's perspective on psychological issues in football	Science & Medicine in Football	No	Abstract and keywords
571	P. Rodriguez-Giustiniani; I. Rollo; S. D. R. Galloway	2022	A preliminary study of the reliability of soccer skill tests within a modified soccer match simulation protocol	Science and Medicine in Football	No	Abstract and keywords
572	A. Denche-Zamorano; N. Mayordomo-Pinilla; S. Barrios-Fernandez; V. Luis-Del Campo; S. Gómez-Paniagua; J. Rojo-Ramos; A. Castillo-Paredes; L. Muñoz-Bermejo	2023	A scientometrics analysis of physical activity and transcranial stimulation research	Medicine (United States)	No	Abstract and keywords
573	B. Kiss; L. Balogh	2019	A study of key cognitive skills in handball using the Vienna test system	Journal of Physical Education & Sport	No	Abstract and keywords
574	S. Ankit; B. Ravi; M. Deepak	2015	A STUDY ON FACTORS INFLUENCING SPORTS CAREER OF STATE LEVEL PLAYERS PARTICIPATING IN DIFFERENT SPORTS	International Journal of Sports Sciences & Fitness	No	Abstract and keywords
575	K. Przednowek; T. Krzeszowski; K. H. Przednowek; P. Lenik	2018	A system for analysing the basketball free throw trajectory based on particle swarm optimization	Applied Sciences (Switzerland)	No	Abstract and keywords
576	B. Low; D. Coutinho; B. Gonçalves; R. Rein; D. Memmert; J. Sampaio	2020	A Systematic Review of Collective Tactical Behaviours in Football Using Positional Data	Sports Medicine	No	Abstract and keywords
577	C. H. Joo; H. Jee	2019	ACTIVITY PROFILES OF TOP-CLASS PLAYERS AND REFEREES AND ACCURACY IN FOUL DECISION-MAKING DURING KOREAN NATIONAL LEAGUE SOCCER GAMES	Journal of Strength and Conditioning Research	No	Abstract and keywords
578	Z. Nikolovski; D. Vrdoljak; N. Foretić; M. Perić; D. Marić; C. Fountoulakis	2023	Acute and Long-Lasting Effects of Slow-Paced Breathing on Handball Team Coach’s Match Stress	Healthcare (Switzerland)	No	Abstract and keywords
579	T. F. D. Kanthack; M. Bigliassi; L. F. Vieira; L. R. Altimari	2014	Acute effect of motor imagery on basketball players' free throw performance and self-efficacy. / Efeito agudo da imag ética no desempenho de lances livres e percepção de autoeficácia em atletas	Brazilian Journal of Kineanthropometry & Human Performance	No	Abstract and keywords
580	L. B. Baker; R. P. Nuccio; A. E. Jeukendrup	2014	Acute effects of dietary constituents on motor skill and cognitive performance in athletes	Nutrition Reviews	No	Abstract and keywords
581	C. Jones	2014	Alcoholism and recovery: A case study of a former professional footballer	International Review for the Sociology of Sport	No	Abstract and keywords
582	G. TekkurŞUn DemİR; H. İ. CİCİOĞLu; Y. E. Yarayan	2023	Algılanan Fiziksel ve Bilişsel Yorgunluk Ölçeği'nin Geliştirilmesi (AFBYÖ): Geçerlik ve Güvenirlik Çalışması. / Developing the Scale of Physical and Cognitive Fatigue Perceived (SPCFP): Validity and Reliability Study	Mediterranean Journal of Sport Science (MJSS)	No	Abstract and keywords
583	J. M. Beus; D. S. Whitman	2017	Almighty Dollar or Root of All Evil? Testing the Effects of Money on Workplace Behavior	Journal of Management	No	Abstract and keywords
584		2007	AMELIE MAURESMO		No	Abstract and keywords

585	D. Coutinho; B. Gonçalves; H. Folgado; B. Travassos; S. Santos; J. Sampaio	2022	Amplifying perceptual demands: How changes in the colour vests affect youth players performance during medium-sided games	PLoS ONE	No	Abstract and keywords
586	M. Jewiss; O. R. Runswick; I. Greenlees	2023	An Examination of the Challenge/Threat State and Sport-Performance Relationship While Controlling for Past Performance	Journal of Sport and Exercise Psychology	No	Abstract and keywords
587	M. Falces-Prieto; F. T. González-Fernández; J. Matas-Bustos; P. J. Ruiz-Montero; J. Rodicio-Palma; M. Torres-Pacheco; F. M. Clemente	2021	An exploratory data analysis on the influence of role rotation in a small-sided game on young soccer players	International Journal of Environmental Research and Public Health	No	Abstract and keywords
588	R. D. Samuel; G. Tenenbaum; Y. Galily	2021	An integrated conceptual framework of decision-making in soccer refereeing	International Journal of Sport and Exercise Psychology	No	Abstract and keywords
589	S. M. Smith; S. T. Cotterill; H. Brown	2020	An interpretative phenomenological analysis of performance influencing factors within the practice environment	Journal of Physical Education & Sport	No	Abstract and keywords
590	M. Mitrotasios; C. Casal; V. Armatas; J. Losada; R. Maneiro	2021	Analysis of Corner Kick Success in Laliga Santander 2019/2020	European Journal of Human Movement	No	Abstract and keywords
591	J. D. Connor; W. H. Sinclair; A. S. Leicht; K. Doma	2019	Analysis of cricket ball type and innings on state level cricket batter’s performance	Frontiers in Psychology	No	Abstract and keywords
592	M. Lorenzo-Martínez; A. Padrón-Cabo; E. Rey; D. Memmert	2021	Analysis of Physical and Technical Performance of Substitute Players in Professional Soccer	Research Quarterly for Exercise and Sport	No	Abstract and keywords
593	I. Teoldo da Costa; M. P. de Freitas Silvino	2023	Analysis of tactical behavior in full- and small-sided games: Comparing professional and youth academy athletes to enhance player development in soccer	International Journal of Sports Science and Coaching	No	Abstract and keywords
594	J. Sasadai; N. Maeda; R. Shimizu; T. Kobayashi; S. Sakai; M. Komiya; Y. Urabe	2020	Analysis of team-sport wheelchair falls during the Rio 2016 Summer Paralympic Games: A video-based cross-sectional observational study	BMJ Open	No	Abstract and keywords
595	T. J. C. Pereira; F. Y. Nakamura; M. T. de Jesus; C. L. R. Vieira; M. S. Misuta; R. M. L. de Barros; F. A. Moura	2017	Analysis of the distances covered and technical actions performed by professional tennis players during official matches	Journal of Sports Sciences	No	Abstract and keywords
596	L. S. C. Ueda; V. M. Menegassi; A. Avelar; L. Rechenchosky; F. L. O. Silva; P. H. Borges	2020	Analysis of the execution of core tactical principles and technical efficiency of primary school futsal players	Revista Brasileira de Cineantropometria e Desempenho Humano	No	Abstract and keywords
597	F. M. Clemente; A. F. Silva; C. C. T. Clark; D. Conte; J. Ribeiro; B. Mendes; R. Lima	2020	Analyzing the seasonal changes and relationships in training load and wellness in elite volleyball players	International Journal of Sports Physiology and Performance	No	Abstract and keywords
598	D. H. Han; B. N. Kim; J. H. Cheong; K. D. Kang; P. F. Renshaw	2014	Anxiety and attention shifting in professional baseball players	International Journal of Sports Medicine	No	Abstract and keywords
599	F. C. A. Nogueira; M. G. B. Filho; L. M. Lourenço	2019	Application of izof model for anxiety and self-efficacy in volleyball athletes: A case study	Revista Brasileira de Medicina do Esporte	No	Abstract and keywords
600	J. Schimpchen; S. Skorski; S. Nopp; T. Meyer	2016	Are “classical” tests of repeated-sprint ability in football externally valid? A new approach to determine in-game sprinting behaviour in	Journal of Sports Sciences	No	Abstract and keywords

			elite football players			
601	V. Zanetti; C. Carling; M. S. Aoki; P. S. Bradley; A. Moreira	2021	Are There Differences in Elite Youth Soccer Player Work Rate Profiles in Congested vs. Regular Match Schedules?	Journal of Strength and Conditioning Research	No	Abstract and keywords
602	J. P. Fuentes-García; J. L. Leon-Llamas; N. Gusi; S. Villafaina	2022	Are there neurophysiological differences behind the play of different chess modalities?: An international grandmaster case study	PHYSIOLOGY & BEHAVIOR	No	Abstract and keywords
603	M. K. Wheeler; C. Hambleton	2014	ARE U.S. YOUTH CLUBS LEAVING MONEY ON THE TABLE?	Soccer Journal	No	Abstract and keywords
604	N. Burger; M. I. Lambert; H. Hall; S. Hendricks	2019	Assessing tackle performance using a novel collision sport simulator in comparison to a "live" one-on-one tackling drill	Journal of Sports Sciences	No	Abstract and keywords
605	E. P. Storey; D. J. Corwin; C. C. McDonald; K. B. Arbogast; K. B. Metzger; M. R. Pfeiffer; S. S. Margulies; M. F. Grady; C. L. Master	2022	Assessment of Saccades and Gaze Stability in the Diagnosis of Pediatric Concussion	Clinical Journal of Sport Medicine	No	Abstract and keywords
606	A. Hof zum Berge; F. Loch; K. Schwarzenbrunner; A. Ferrauti; T. Meyer; M. Pfeiffer; M. Kellmann	2021	Assessment of sleep quality and daytime sleepiness in German national ice hockey players preparing for the world championship	German Journal of Exercise and Sport Research	No	Abstract and keywords
607	A. J. Pearce; D. King; D. J. Kidgell; A. K. Frazer; M. Tommerdahl; C. M. Suter	2022	Assessment of Somatosensory and Motor Processing Time in Retired Athletes with a History of Repeated Head Trauma	JOURNAL OF FUNCTIONAL MORPHOLOGY AND KINESIOLOGY	No	Abstract and keywords
608	J. Charest; J. D. Cook; A. M. Bender; O. Walch; M. A. Grandner; C. H. Samuels	2022	Associations between time zone changes, travel distance and performance: A retrospective analysis of 2013–2020 National Hockey League Data	Journal of Science and Medicine in Sport	No	Abstract and keywords
609	R. d. O. Moreira Rodrigues; D. Ribeiro de Almeida; V. P. Neves Miranda; D. Alvarez Pires; E. Macedo Penna; M. Vidigal Miranda Júnior	2021	AVALIAÇÃO DO BURNOUT EM JOGADORES DE FUTEBOL. / BURNOUT ASSESSMENT IN SOCCER PLAYERS	Brazilian Journal of Soccer Science / Revista Brasileira de Futebol	No	Abstract and keywords
610	E. Gonçalves; H. Sarmento; I. Teoldo; A. Tessitore; A. J. Figueiredo	2022	AVALIAÇÃO E MONITORAMENTO DE JOVENS JOGADORES DE FUTEBOL: UMA REVISÃO SISTEMÁTICA. / EVALUATION AND MONITORING OF YOUNG SOCCER PLAYERS: A SYSTEMATIC REVIEW	Brazilian Journal of Soccer Science / Revista Brasileira de Futebol	No	Abstract and keywords
611	M. Lopes; S. Lopes; T. Patinha; F. Araújo; M. Rodrigues; R. Costa; J. Oliveira; F. Ribeiro	2019	Balance and proprioception responses to FIFA 11+ in amateur futsal players: Short and long-term effects	Journal of Sports Sciences	No	Abstract and keywords
612	Y. K. Chiu; C. Y. Pan; F. C. Chen; Y. T. Tseng; C. L. Tsai	2020	Behavioral and cognitive electrophysiological differences in the executive functions of taiwanese basketball players as a function of playing position	Brain Sciences	No	Abstract and keywords
613	F. Dambroz; I. Teoldo	2023	Better decision-making skills support tactical behaviour and reduce physical wear under physical fatigue in soccer	Frontiers in Physiology	No	Abstract and keywords
614	A. P. Friesen; T. J. Devonport; A. M. Lane	2017	Beyond the technical: The role of emotion regulation in lacrosse officiating	Journal of Sports Sciences	No	Abstract and keywords

615	B. H. M. Toapanta; M. E. P. Ruiz; M. A. P. Peralta; A. R. B. López; E. R. Frómeta; L. M. Pedroso	2018	Biomechanical differences and effectiveness of basketball free throw in optimal state and fatigue	Revista Cubana de Investigaciones Biomedicas	No	Abstract and keywords
616	R. İri; E. Şengür	2021	BİYO-GRUPLAMAYA GÖRE VOLEYBOLCULARIN DİKEY SIÇRAMA, ÇEVİKLİK VE SÜRAT PERFORMANSLARININ KARŞILAŞTIRILMASI. / COMPARISON OF VOLLEYBALL PLAYERS OF VERTICAL JUMP, AGILITY AND SPEED PERFORMANCE ACCORDING TO BIO-BANDING	SPORMETRE: The Journal of Physical Education & Sport Sciences / Beden Eğitimi ve Spor Bilimleri Dergisi	No	Abstract and keywords
617	M. F. Hidayatullah; M. Doewes; S. K. Purnama; S. Riyadi; R. Syaifullah	2022	BOTELI and Interval Training to Improve Tennis Groundstroke Forehand's Rally Skills	International Journal of Human Movement and Sports Sciences	No	Abstract and keywords
618	L. S. Fortes; M. E. C. Ferreira; H. Faro; E. M. Penna; S. S. Almeida	2022	Brain Stimulation Over the Motion-Sensitive Midtemporal Area Reduces Deleterious Effects of Mental Fatigue on Perceptual–Cognitive Skills in Basketball Players	Journal of Sport & Exercise Psychology	No	Abstract and keywords
619	Q. Gao; L. Zhang	2023	Brief mindfulness meditation intervention improves attentional control of athletes in virtual reality shooting competition: Evidence from fNIRS and eye tracking	Psychology of Sport and Exercise	No	Abstract and keywords
620	A. T. Cook	2023	Buford Complex in a High School Softball Player with Glenohumeral Multidirectional Instability and Cubital Tunnel Syndrome with Ulnar Nerve Subluxation	Journal of Sports Medicine & Allied Health Sciences: Official Journal of the Ohio Athletic Trainers' Association	No	Abstract and keywords
621	M. D. Santos-Afonso; L. G. Lourenção; M. D. S. Afonso; M. D. O. Saes; F. B. D. Santos; J. G. M. Penha; D. M. Galvão; F. R. G. Ximenes Neto; N. S. G. M. D. S. Sasaki; M. D. L. S. G. Santos; F. A. Borges; J. F. D. Oliveira; S. T. Rodrigues; E. D. O. Bandeira; F. C. D. Alcantara; C. L. F. Cunha; F. G. D. Silva; M. Lemos; A. D. O. Soares Junior; F. B. Neves	2023	Burnout Syndrome in Selectable Athletes for the Brazilian Handball Team—Children Category	International Journal of Environmental Research and Public Health	No	Abstract and keywords
622	K. Van Meervelt; S. Van Puyenbroeck; G. Vande Broek	2023	Can participative coach behaviour be perceived as controlling? The role of athletes' expectations	European Journal of Sport Science	No	Abstract and keywords
623	A. Vaquera; J. Mielgo-Ayuso; J. Calleja-González; A. S. Leicht	2017	Cardiovascular and perceptual stress of female basketball referees during women's International matches	Journal of Sports Medicine and Physical Fitness	No	Abstract and keywords
624	J. Vázquez-Guerrero; B. Fernández-Valdés; B. Gonçalves; J. E. Sampaio	2019	Changes in locomotor ratio during basketball game quarters from elite under-18 teams	Frontiers in Psychology	No	Abstract and keywords
625	A. Gutiérrez-Capote; I. Madinabeitia; E. Torre; F. Alarcón; J. Jiménez-Martínez; D. Cárdenas	2023	Changes in Perceived Mental Load and Motor Performance during Practice-to-Learn and Practice-to-Maintain in Basketball	International Journal of Environmental Research and Public Health	No	Abstract and keywords

626	K. Barczyk-pawelec; K. Rubajczyk; M. Stefańska; Ł. Pawik; W. Dziubek	2022	Characteristics of Body Posture in the Sagittal Plane in 8–13-Year-Old Male Athletes Practicing Soccer	Symmetry	No	Abstract and keywords
627	A. J. Pearce; D. J. Kidgell; M. A. Tommerdahl; A. K. Frazer; B. Rist; R. Mobbs; J. Batchelor; M. E. Buckland	2021	Chronic Neurophysiological Effects of Repeated Head Trauma in Retired Australian Male Sport Athletes	Front Neurol	No	Abstract and keywords
628	E. Facer-Childs; R. Brandstaetter	2015	Circadian phenotype composition is a major predictor of diurnal physical performance in teams	Frontiers in Neurology	No	Abstract and keywords
629	H. Gunnarsson; J. Agerström	2018	Clinical pain, abstraction, and self-control: Being in pain makes it harder to see the forest for the trees and is associated with lower self-control	Journal of Pain Research	No	Abstract and keywords
630	R. A. Birrento; J. M. Giménez-Egido; E. Ortega; R. A. Birrento Aguiar	2023	COACHES AND EXPERTS’ OPINION ABOUT BASKETBALL MODIFICATION RULES IN UNDER 13 LEVEL	Journal of Sport and Health Research	No	Abstract and keywords
631	D. D. Grecic; M. B. Ryan	2018	Coaching Golf -- How skilled are we in 'skill'?	Sport Journal	No	Abstract and keywords
632	D. Elmes	2018	Coach-player communications: An analysis of top-level coaching discourse at a short-term ice hockey camp	Humanities and Social Sciences Reviews	No	Abstract and keywords
633	R. R. Azevedo; F. P. Carpes	2021	Cognitive and neuromuscular influences on perceived effort during a competitive season in futsal	Apunts Sports Medicine	No	Abstract and keywords
634	D. Milne; G. Morrison	2015	Cognitive behavioural intervention for the golf yips: A single-case design	Sport & Exercise Psychology Review	No	Abstract and keywords
635	D. Goble; C. J.-A. Christie	2017	Cognitive, physical and physiological responses of school boy cricketers to a 30-over batting simulation	Journal of Sports Sciences	No	Abstract and keywords
636	M. Bouchiba; N. L. Bragazzi; S. Zarzissi; M. Turki; F. Zghal; M. A. Grati; W. Daab; F. Ayadi; H. Rebai; H. Ibn Hadj Amor; T. J. Hureau; M. A. Bouzid	2022	Cold Water Immersion Improves the Recovery of Both Central and Peripheral Fatigue Following Simulated Soccer Match-Play	Frontiers in Physiology	No	Abstract and keywords
637	A. Green; C. Dafkin; S. Kerr; W. McKinon	2017	Combined individual scrummaging kinetics and muscular power predict competitive team scrum success	European Journal of Sport Science	No	Abstract and keywords
638	A. Rabbani; F. M. Clemente; M. Kargarfard; S. Jahangiri	2019	Combined small-sided game and high-intensity interval training in soccer players: The effect of exercise order	Journal of Human Kinetics	No	Abstract and keywords
639	A. Manholer; A. Oliveira; R. Denis; R. Chaves; S. G. da Silva; R. F. dos Santos Legnani; E. Legnani	2019	COMPARAÇÃO ENTRE AS RESPOSTAS PSICOFISIOLÓGICAS EM TESTE INCREMENTAL NA E ESTEIRA E JOGOS DE FUTSAL ADAPTADOS. / Comparison between psychophysiological responses in incremental test in the match and adapted futsal games	Revista Brasileira de Prescrição e Fisiologia do Exercício	No	Abstract and keywords
640	R. R. dos Santos Filho; A. Fontes; F. Cardoso	2021	COMPARAÇÃO ENTRE O NÍVEL DE CONHECIMENTO TÁTICO PROCESSUAL DE JOGADORES DE FUTEBOL DAS CATEGORIAS SUB-11 E SUB-13. / COMPARISON BETWEEN THE PROCEDURAL TACTICAL	Brazilian Journal of Soccer Science / Revista Brasileira de Futebol	No	Abstract and keywords

			KNOWLEDGE LEVEL OF SOCCER PLAYERS IN THE UNDER-11 AND UNDER-13 CATEGORIES			
641	G. He	2022	Comparative Study on Biomechanics of Two Legs in the Action of Single-Leg Landing in Men's Badminton	MCB Molecular and Cellular Biomechanics	No	Abstract and keywords
642	M. Oytun; M. Çakıcı; C. Tınazcı; H. U. Yavuz	2021	Comparison of anxiety and narcissism levels of different performance groups in female handball players	Anadolu Psikiyatri Dergisi	No	Abstract and keywords
643	A. Ploudre; J. L. Arabas; L. I. Z. Jorn; J. L. Mayhew	2018	Comparison of Techniques for Tracking Body Composition Changes across a Season in College Women Basketball Players	International Journal of Exercise Science	No	Abstract and keywords
644	X. Chen; R. Zheng; B. Xiong; X. Huang; B. Gong	2023	Comparison of the physiological responses and time-motion characteristics during football small-sided games: effect of pressure on the ball	Frontiers in Physiology	No	Abstract and keywords
645	B. S. H. de Faria; Y. de Almeida Costa Campos; H. T. Toledo; R. Miranda; J. M. Vianna; M. G. B. Filho	2020	Comparison of the training load of professional athletes between modes of volleyball specific drills and strength conditioning	Journal of Physical Education (Maringa)	No	Abstract and keywords
646	J. Schaefer; S. A. Vella; M. S. Allen; C. A. Magee	2016	Competition Anxiety, Motivation, and Mental Toughness in Golf	Journal of Applied Sport Psychology	No	Abstract and keywords
647	V. Kostiukevych; N. Lazarenko; V. Adamchuk; N. Shchepotina; T. Vozniuk; O. Shynkaruk; I. Asauliuk; S. Konnov; S. Voitenko	2023	COMPREHENSIVE ASSESSMENT OF THE PREPAREDNESS OF HIGHLY QUALIFIED FIELD HOCKEY PLAYERS AT THE STAGE OF DIRECT PREPARATION FOR THE MAIN COMPETITIONS	Physical Education Theory and Methodology	No	Abstract and keywords
648	R. Pereira da Silva; H. Izais Marcelo; D. Leite Lemos; M. Tetila Felix; R. Chaccon Castoldi; E. A. Carvalho Zanuto	2020	CONCENTRAÇÕES DE LACTATO, CREATINAQUINASE E LACTATODESIDROGENASE EM JOGADORES DE FUTEBOL AMADOR EXPOSTOS A CRIOTERAPIA POR IMERSÃO E EFICIÊNCIA DA PERCEPÇÃO SUBJETIVA DO ESFORÇO. / LACTATE, CREATINE KINASE, AND LACTATE DEHYDROGENASE CONCENTRATIONS IN AMATEUR SOCCER PLAYERS EXPOSED TO IMMERSION CRYOTHERAPY AND EFFICIENCY OF THE SUBJECTIVE PERCEPTION OF EFFORT	Brazilian Journal of Soccer Science / Revista Brasileira de Futebol	No	Abstract and keywords
649	G. Macartney; V. Simoncic; K. Goulet; M. Aglipay	2018	Concussion Symptom Prevalence, Severity and Trajectory: Implications for Nursing Practice	Journal of Pediatric Nursing	No	Abstract and keywords
650	A. Los Arcos; A. Gonzalez-Artetxe; U. Bayer-Perez; H. Folgado	2023	Consequences of field obstacles on tactical, conditional, and emotional dimensions in young female soccer players during small-sided games	Proceedings of the Institution of Mechanical Engineers, Part P: Journal of Sports Engineering and Technology	No	Abstract and keywords
651	M. A. R. Alves; D. C. Da Graça; B. Travassos	2022	Construction and validation of an observation tool of the imbalance pass in futsal	Revista Brasileira de Cineantropometria e Desempenho Humano	No	Abstract and keywords
652	P. Sainam; S. Balasubramanian; B. L. Bayus	2015	Consumer Forwards: Concept and Empirical Analysis of a Sports Ticket Market	International Journal of Sport Finance	No	Abstract and keywords
653	G. Hallé Petiot; R. Aquino; D. C. D. Silva; D.	2021	Contrasting Learning Psychology Theories Applied to the Teaching-Learning-Training	Frontiers in Psychology	No	Abstract and keywords

	V. Barreira; M. Raab		Process of Tactics in Soccer			
654	G. Machado; S. González-Víllora; I. Teoldo	2022	Contribution of deliberate practice, play, and futsal to the acquisition of decision-making skills in Brazilian professional female soccer players	International Journal of Sport and Exercise Psychology	No	Abstract and keywords
655	G. Martinent; J.-C. Decret	2015	Coping profiles of young Athletes in their everyday life: A three-wave two-month study	European Journal of Sport Science	No	Abstract and keywords
656	J. Habay; M. Proost; J. De Wachter; J. Díaz-García; K. De Pauw; R. Meeusen; J. Van Cutsem; B. Roelands	2023	Correction: Mental Fatigue-Associated Decrease in Table Tennis Performance: Is There an Electrophysiological Signature? (Int. J. Environ. Res. Public Health, (2021), 18, 12906, 10.3390/ijerph182412906)	International Journal of Environmental Research and Public Health	No	Abstract and keywords
657	Z. Muthahari; D. T. Mario; Y. Astuti	2023	Court Tennis Referee Decision Making: How does Mastery of Game Rules, Stress Management, and Concentration Affect it?	International Journal of Human Movement and Sports Sciences	No	Abstract and keywords
658	B. L. Hawkins; G. Ramshaw; T. Hooker; K. Walker	2020	Creating Football Memory Teams: Development and Evaluation of a Football-Themed Reminiscence Therapy Program	Therapeutic Recreation Journal	No	Abstract and keywords
659	C. Magee; A. Wolaver	2023	Crowds and the Timing of Goals and Referee Decisions	Journal of Sports Economics	No	Abstract and keywords
660	S. Ryan; T. Kempton; A. J. Coutts	2021	Data Reduction Approaches to Athlete Monitoring in Professional Australian Football	International Journal of Sports Physiology & Performance	No	Abstract and keywords
661	T. J. Hepler; M. Andre	2021	Decision outcomes in sport: Influence of type and level of stress	Journal of Sport and Exercise Psychology	No	Abstract and keywords
662	R. Sánchez-López; I. Echeazarra; J. M. Arrieta; J. Castellano	2023	Declarative tactical knowledge from 12 to 22 years old in a professional football club: Real Sociedad	International Journal of Sports Science and Coaching	No	Abstract and keywords
663	S. L. Schmidt; G. J. Schmidt; C. S. Padilla; E. N. Simões; J. C. Tolentino; P. R. Barroso; J. H. Narciso; E. S. Godoy; R. L. Costa Filho	2019	Decrease in Attentional Performance After Repeated Bouts of High Intensity Exercise in Association-Football Referees and Assistant Referees	Frontiers in Psychology	No	Abstract and keywords
664	M. Naughton; T. Scott; D. Weaving; C. Solomon; S. McLean	2023	Defining and quantifying fatigue in the rugby codes	PLoS ONE	No	Abstract and keywords
665	M. E. d. S. Nunes; U. C. Correa; M. G. T. X. de Souza; S. Santos	2021	Descriptive versus prescriptive feedback in the learning of golf putting by older persons	International Journal of Sport & Exercise Psychology	No	Abstract and keywords
666	Y. W. Ma; J. L. Chen; C. C. Hsu; Y. H. Lai	2021	Design and Analysis of a Pitch Fatigue Detection System for Adaptive Baseball Learning	Frontiers in Psychology	No	Abstract and keywords
667	G. A. Calle-Jaramillo; E. V. Gonzalez-Palacio; L. A. Perez-Mendez; A. Rojas-Jaramillo; J. A. Gonzalez-Jurado	2023	Design and Validation of a Test to Evaluate the Execution Time and Decision-Making in Technical–Tactical Football Actions (Passing and Driving)	Behavioral Sciences	No	Abstract and keywords
668	R. Boat; C. Sunderland; S. B. Cooper	2021	Detrimental effects of prior self-control exertion on subsequent sporting skill performance	Scandinavian Journal of Medicine & Science in Sports	No	Abstract and keywords
669	A. Ramos; P. Coutinho; K. Davids; I. Mesquita	2020	Developing Players' Tactical Knowledge Using Combined Constraints-Led and Step-Game Approaches—A Longitudinal Action-Research Study	Research Quarterly for Exercise and Sport	No	Abstract and keywords

670	L. Thieschäfer; J. Klütz; J. Weig; T. Dos'Santos; D. Büsch	2023	Development of a Cutting Technique Modification Training Program and Evaluation of its Effects on Movement Quality and Cutting Performance in Male Adolescent American Football Players	Sports (2075-4663)	No	Abstract and keywords
671	M. S. Taufik; E. F. Amalia	2021	Development of defensive training futsal model university of suryakancana	International Journal of Human Movement and Sports Sciences	No	Abstract and keywords
672	M. Pelka; P. Schneider; M. Kellmann	2018	Development of pre- and post-match morning recovery-stress states during in-season weeks in elite youth football	Science and Medicine in Football	No	Abstract and keywords
673	A. P. Cooley; S. L. Grace; P. Parker; J. A. Bunn	2023	Difference in Perceived Wellness and Training by Varying Psychological Hardiness in Division I Female Lacrosse Athletes	Journal of Sport Behavior	No	Abstract and keywords
674	G. De Sousa Pinheiro; H. S. Bernardino; I. T. Costa; V. T. Costa	2021	Differences in discriminative reaction time between elite youth football players: A comparison between age-categories	Retos	No	Abstract and keywords
675	M. Mitrotasios	2018	Differences in Performance Indicators between successful and unsuccessful Teams in UEFA-EURO 2012	Biology of Exercise	No	Abstract and keywords
676	D. Coutinho; B. Gonçalves; B. Travassos; H. Folgado; B. Figueira; J. Sampaio	2020	Different Marks in the Pitch Constraint Youth Players' Performances During Football Small-sided Games	Research Quarterly for Exercise & Sport	No	Abstract and keywords
677	F. Tokat; K. KeskİN	2023	Do Mental Training Level and Perceived Fatigue Affect Competition Outcome in Volleyball? / Zihinsel Antrenman Düzeyi ve Algılanan Yorgunluk Voleybolda Müsabaka Sonucunu Etkiler Mi?	Mediterranean Journal of Sport Science (MJSS)	No	Abstract and keywords
678	J. Vítor de Assis; S. González-Víllora; F. M. Clemente; F. Cardoso; I. Teoldo	2020	Do youth soccer players with different tactical behaviour also perform differently in decision-making and visual search strategies?	International Journal of Performance Analysis in Sport	No	Abstract and keywords
679	S. Sukys; I. Tilindienė; V. J. Cesnaitiene; R. Kreivyte	2019	Does Emotional Intelligence Predict Athletes' Motivation to Participate in Sports?	Perceptual & Motor Skills	No	Abstract and keywords
680	A. J. Picazo-Tadeo; F. González-Gómez; J. Guardiola	2017	Does the crowd matter in refereeing decisions? Evidence from Spanish soccer	International Journal of Sport & Exercise Psychology	No	Abstract and keywords
681	M. Turner; P. Beranek; J. Lo; A. Ferrauti; I. C. Dunican; T. Cruickshank	2022	Does time of day and player chronotype impact tennis-specific skills and physical performance?	International Journal of Sports Science and Coaching	No	Abstract and keywords
682	G. Gallicchio; C. Ring	2019	Don't look, don't think, just do it! Toward an understanding of alpha gating in a discrete aiming task	Psychophysiology	No	Abstract and keywords
683	T. van Duijn; A. Cooke; E. Bellomo; R. Masters	2017	EEG COHERENCE AND CONSCIOUS MOTOR PROCESSING IN GOLF PUTTING BEGINNERS	New Zealand Journal of Sports Medicine	No	Abstract and keywords
684	L. Pomportes; K. Davranche; A. Hays; J. Brisswalter	2015	Effect of a creatine-guaranà complex on muscular power and cognitive performance in high-level athletes	Science and Sports	No	Abstract and keywords
685	R. V. Gomes; A. Moreira; A. J. Coutts; C. D. Capitani; M. S. Aoki	2014	Effect of carbohydrate supplementation on the physiological and perceptual responses to prolonged tennis match play	Journal of Strength and Conditioning Research	No	Abstract and keywords
686	S. Mohammad Amoli; P. Aghaie Ataabadi; A. Letafatkar; G. B. Wilkerson; M. B.	2021	Effect of Cognitive Loading on Single-Leg Jump Landing Biomechanics of Elite Male Volleyball Players	International Journal of Athletic Therapy & Training	No	Abstract and keywords

	Mansouri					
687	J. A. Griffin; C. P. McLellan; J. Presland; C. T. Woods; J. W. L. Keogh	2017	Effect of defensive pressure on international women's rugby sevens attacking skills frequency and execution	International Journal of Sports Science & Coaching	No	Abstract and keywords
688	A. Bourara; Z. Németh; J. Methnani; M. Wilhelm	2023	Effect of exhaustion on dynamic balance of professional padel players	Sport Sciences for Health	No	Abstract and keywords
689	A. Rusdiana; H. Subarjah; I. Imanudin; Y. Kusdinar; A. M. Syahid; T. Kurniawan	2020	Effect of Fatigue on Biomechanical Variable Changes in Overhead Badminton Jump Smash	Annals of Applied Sport Science	No	Abstract and keywords
690	P. C. Lazarraga	2023	Effect of implicit teaching strategy on basketball players	VISUAL Review. International Visual Culture Review / Revista Internacional de Cultura	No	Abstract and keywords
691	A. Metelski; K. Kornakov	2021	Effect of lockdown owing to COVID-19 on players' match statistics in Bundesliga	Journal of Physical Education & Sport	No	Abstract and keywords
692	L. de Sousa Fortes; S. S. Almeida; J. R. A. Nascimento; L. Fiorese; D. Lima; M. E. C. Ferreira	2019	Effect of motor imagery training on tennis service performance in young tennis athletes	Revista de Psicologia del Deporte	No	Abstract and keywords
693	P. Shi; Z. Zhang; X. Feng; C. Li; Y. Tang	2023	Effect of physical exercise in real-world settings on executive function of atypical children: A systematic review and meta-analysis	Child: Care, Health and Development	No	Abstract and keywords
694	U. L. Yeole; N. Praneetha; C. Panigrahy	2023	Effect of Position Specific Physiotherapy Training on performance of Indian football players: A Randomized Control Trial	Research in Sports Medicine	No	Abstract and keywords
695	S. Mouelhi-Guizani; S. Guinoubi; N. Teyeb; M. Chtara; M. Crespo	2022	Effect of practice hours on elite junior tennis players' burnout: Gender differences	International Journal of Sports Science & Coaching	No	Abstract and keywords
696	T. Wiewelhove; C. Raeder; T. Meyer; M. Kellmann; M. Pfeiffer; A. Ferrauti	2016	Effect of Repeated Active Recovery During a High-Intensity Interval-Training Shock Microcycle on Markers of Fatigue	International Journal of Sports Physiology & Performance	No	Abstract and keywords
697	M. J. M. Al-Khalidi; D. T. H. Al-Janbi	2018	Effect of resistance exercises by means of AIDS with doses of vitamin D3 in some biomechanical variants to develop the spike skill of volleyball for female students	Journal of Global Pharma Technology	No	Abstract and keywords
698	K. J. Jaquess; Y. Lu; A. Ginsberg; S. Kahl Jr; C. Lu; B. Ritland; R. J. Gentili; B. D. Hatfield	2021	Effect of Self-Controlled Practice on Neuro-Cortical Dynamics During the Processing of Visual Performance Feedback	Journal of Motor Behavior	No	Abstract and keywords
699	H. O. Abdulamir; I. N. Kazem; M. A. Mohammed Ali; H. N. Habash Alshawi	2019	Effect of special exercises for the development of some biochemical variables and the level of performance of diagonal spike skill in the volleyball	Journal of Global Pharma Technology	No	Abstract and keywords
700	T. W. Spooner; A. T. West; M. E. T. Willems	2023	Effect of Substitution Time on Physical, Technical and Cognitive Performance in Sub-Elite Male Field Hockey Players	International Journal of Exercise Science	No	Abstract and keywords
701	J. López-Aguilar; I. Alonso-Arbiol; W. Onetti-Onetti; A. Castillo-Rodríguez	2021	Effect of the competition on amateur soccer referee impulsivity	Cultura, Ciencia y Deporte	No	Abstract and keywords
702	J. C. Ponce-Bordón; M. A. López-Gajardo; F. M. Leo; J. J. Pulido; T. García-Calvo	2021	Effect of training-task orientation in women’s football	Revista Internacional de Medicina y Ciencias de la Actividad Fisica y del Deporte	No	Abstract and keywords

703	J. C. Ponce-Bordón; M. A. López-Gajardo; F. M. Leo; J. J. Pulido; T. García-Calvo	2021	EFFECT OF TRAINING-TASK ORIENTATION IN WOMEN'S FOOTBALL. / EFECTO DE LA ORIENTACIÓN DE LAS TAREAS DE ENTRENAMIENTO EN FÚTBOL FEMENINO	International Journal of Medicine & Science of Physical Activity & Sport / Revista Internacional de Medicina y Ciencias de la Actividad Física y del Deporte	No	Abstract and keywords
704	J. F. Guzmán; J. Madera; D. Marín-Suelves; J. Ramón-Llin	2022	Effects of a notational analysis-based intervention on coaches' verbal behaviour according to physiological activation during competition	Heliyon	No	Abstract and keywords
705	K. Redman; K. Steel; V. Kelly; J. Siegler	2021	Effects of a rugby league match simulation on decision-making in elite junior rugby league	Journal of Strength and Conditioning Research	No	Abstract and keywords
706	F. Di Rienzo; P. Joassy; T. Kanthack; T. E. MacIntyre; U. Debarnot; Y. Blache; C. Hautier; C. Collet; A. Guillot	2019	Effects of Action Observation and Action Observation Combined with Motor Imagery on Maximal Isometric Strength	Neuroscience	No	Abstract and keywords
707	M. Loiseau-Taupin; A. Ruffault; J. Slawinski; L. Delabarre; D. Bayle	2021	Effects of Acute Physical Fatigue on Gaze Behavior and Performance During a Badminton Game	Frontiers in Sports and Active Living	No	Abstract and keywords
708	E. S. H. Murray; G. L. Hands; C. R. Calabrese; C. E. Stepp	2016	Effects of Adventitious Acute Vocal Trauma: Relative Fundamental Frequency and Listener Perception	Journal of Voice	No	Abstract and keywords
709	A. Pompeo; E. L. R. Cirillo; J. A. H. Da Costa; J. Vilaça-Alves; A. M. Williams; R. Ramirez-Campillo; J. A. Neves; F. Casanova	2023	Effects of ambient temperature on physical and physiological demands and player's judgment ability assessed by a football-specific fatigue-inducing protocols: A systematic review	Journal of Human Sport and Exercise	No	Abstract and keywords
710	A. Silva; R. Ferraz; L. Branquinho; T. Dias; J. E. Teixeira; D. A. Marinho	2023	Effects of applying a multivariate training program on physical fitness and tactical performance in a team sport taught during physical education classes	Frontiers in Sports and Active Living	No	Abstract and keywords
711	J. E. Teixeira; A. R. Alves; R. Ferraz; P. Forte; M. Leal; J. Ribeiro; A. J. Silva; T. M. Barbosa; A. M. Monteiro	2022	Effects of Chronological Age, Relative Age, and Maturation Status on Accumulated Training Load and Perceived Exertion in Young Sub-Elite Football Players	Frontiers in Physiology	No	Abstract and keywords
712	A. M. G. C. P. Adikari; M. Appukutty; G. Kuan	2020	Effects of daily probiotics supplementation on anxiety induced physiological parameters among competitive football players	Nutrients	No	Abstract and keywords
713	L. García-González; A. Moreno; A. Gil; M. P. Moreno; F. D. Villar	2014	Effects of Decision Training on Decision Making and Performance in Young Tennis Players: An Applied Research	Journal of Applied Sport Psychology	No	Abstract and keywords
714	J. C. M. Barte; A. Nieuwenhuys; S. A. E. Geurts; M. A. J. Kompier	2017	Effects of fatigue on soccer performance and the role of task Motivation	International Journal of Sport Psychology	No	Abstract and keywords
715	A. Nuño; I. J. Chirosa; R. van den Tillaar; R. Guisado; I. Martín; I. Martinez; L. J. Chirosa	2016	Effects of Fatigue on Throwing Performance in Experienced Team Handball Players	Journal of Human Kinetics	No	Abstract and keywords
716	H. MacLeod; S. Cooper; S. Bandelow; R. Malcolm; C. Sunderland	2018	Effects of heat stress and dehydration on cognitive function in elite female field hockey players	BMC Sports Science, Medicine & Rehabilitation	No	Abstract and keywords
717	S. Santos; D. Coutinho; B. Gonçalves; E. Abade; B. Pasquarelli; J. Sampaio	2020	Effects of manipulating ball type on youth footballers' performance during small-sided games	International Journal of Sports Science & Coaching	No	Abstract and keywords

718	J. E. Teixeira; M. Leal; R. Ferraz; J. Ribeiro; J. M. Cachada; T. M. Barbosa; A. M. Monteiro; P. Forte	2021	Effects of match location, quality of opposition and match outcome on match running performance in a portuguese professional football team	Entropy	No	Abstract and keywords
719	L. Filipas; S. Borghi; A. La Torre; M. R. Smith	2021	Effects of mental fatigue on soccer-specific performance in young players	SCIENCE AND MEDICINE IN FOOTBALL	No	Abstract and keywords
720	K. Tebourski; M. Bernier; M. Ben Salha; N. Souissi; J. F. Fournier	2022	Effects of Mindfulness for Performance Programme on Actual Performance in Ecological Sport Context: Two Studies in Basketball and Table Tennis	International Journal of Environmental Research and Public Health	No	Abstract and keywords
721	Q. Nian; W. Lu; Y. Xu	2023	Effects of object working memory load on visual search in basketball players: an eye movement study	BMC Psychology	No	Abstract and keywords
722	R. Ramirez-Campillo; J. Sanchez-Sanchez; B. Romero-Moraleda; J. Yanci; A. García-Hermoso; F. Manuel Clemente	2020	Effects of plyometric jump training in female soccer player's vertical jump height: A systematic review with meta-analysis	Journal of Sports Sciences	No	Abstract and keywords
723	N. Deng; K. G. Soh; B. Abdullah; D. Huang; W. Xiao; H. Liu	2023	Effects of plyometric training on technical skill performance among athletes: A systematic review and meta-analysis	PLoS ONE	No	Abstract and keywords
724	J. M. M. Maia Junior; D. B. de Mello; G. Rosa; L. A. dos Santos; R. A. M. Nunes; R. G. de Souza Vale	2023	Effects of scoring method on the physical, technical, and tactical performances during football smallsided games (SSGs): A systematic review	Retos	No	Abstract and keywords
725	S. J. Ibáñez; E. Pérez-Goye; J. García-Rubio; J. Courel-Ibáñez	2020	Effects of task constraints on training workload in elite women's soccer	International Journal of Sports Science and Coaching	No	Abstract and keywords
726	Y. Akinci; S. Kirazci	2020	Effects of Visual, Verbal, Visual + Verbal Feedback on Learning of Dribbling and Lay-up Skill	Sport Mont	No	Abstract and keywords
727	M. MladenoviĆ	2019	ELITE ATHLETES' ASSESSMENT OF MENTAL STATE FOR COMPETITION IN INDIVIDUAL AND TEAM SPORTS. / PROCENA MENTALNOG STANJA ZA TAKMIČENJE VRHUNSKIH SPORTISTA U INDIVIDUALNOM I KOLEKTIVNOM SPORTU	Sports Science & Health / Sportske Nauke i Zdravlje	No	Abstract and keywords
728	G. A. van Kleef; A. Cheshin; L. F. Koning; S. A. Wolf	2019	Emotional games: How coaches' emotional expressions shape players' emotions, inferences, and team performance	Psychology of Sport and Exercise	No	Abstract and keywords
729	A. Camacho-Cardenosa; M. Camacho-Cardenosa; J. Brazo-Sayavera	2019	ENDURANCE ASSESSMENT IN HANDBALL: A SYSTEMATIC REVIEW	European Journal of Human Movement	No	Abstract and keywords
730	Y. Li; B. Li; X. Wang; W. Fu; B. Dai; G. P. Nassis; B. E. Ainsworth	2020	Energetic profile in forehand loop drive practice with well-trained, young table tennis players	International Journal of Environmental Research and Public Health	No	Abstract and keywords
731	H. Nishizawa; T. Kimura	2017	Enhancement of motor skill learning by a combination of ideal model-observation and self-observation	Journal of Physical Therapy Science	No	Abstract and keywords
732	T. Dekkers; K. O'Sullivan; C. Blake; J. G. McVeigh; K. Collins	2022	Epidemiology and moderators of injury in Gaelic football: A systematic review and meta-analysis	Journal of Science and Medicine in Sport	No	Abstract and keywords
733	A. S. D. Gamble; J. L. Bigg; T. F. Vermeulen; S.	2019	Estimated Sweat Loss, Fluid and Carbohydrate Intake, and Sodium Balance of	International Journal of Sport Nutrition & Exercise	No	Abstract and keywords

	M. Boville; G. S. Eskedjian; S. Jannas-Vela; J. Whitfield; M. S. Palmer; L. L. Spriet		Male Major Junior, AHL, and NHL Players During On-Ice Practices	Metabolism		
734	K. Thomas; J. Dent; G. Howatson; S. Goodall	2017	Etiology and Recovery of Neuromuscular Fatigue after Simulated Soccer Match Play	Medicine and Science in Sports and Exercise	No	Abstract and keywords
735		2021	Evaluating the Impact of Concentrated Match Scheduling in College Volleyball during the COVID-19 Pandemic	Sport Journal	No	Abstract and keywords
736	B. S. Willer; M. R. Tiso; M. N. Haider; A. L. Hinds; J. G. Baker; J. C. Miecznikowski; J. J. Leddy	2018	Evaluation of Executive Function and Mental Health in Retired Contact Sport Athletes	Journal of Head Trauma Rehabilitation	No	Abstract and keywords
737	J. Calleja-González; N. Terrados; J. Mielgo-Ayuso; A. Delextrat; I. Jukic; A. Vaquera; L. Torres; X. Schelling; M. Stojanovic; S. M. Ostojic	2016	Evidence-based post-exercise recovery strategies in basketball	Physician and Sportsmedicine	No	Abstract and keywords
738	J. Calleja-González; J. Mielgo-Ayuso; S. M. Ostojic; M. T. Jones; D. Marques-Jiménez; T. Caparros; N. Terrados	2019	Evidence-based post-exercise recovery strategies in rugby: a narrative review	Physician and Sportsmedicine	No	Abstract and keywords
739	N. Haller; E. Hübler; T. Stöggl; P. Simon	2022	Evidence-Based Recovery in Soccer - Low-Effort Approaches for Practitioners	Journal of Human Kinetics	No	Abstract and keywords
740	H. Ulucan; İ. AdİLoĞUllari; D. ÜNver	2014	EXAMINATION OF THE RELATIONSHIP BETWEEN OCCUPATIONAL BURNOUT AND JOB SATISFACTION OF PROFESSIONAL FOOTBALL PLAYERS. / PROFESYONEL FUTBOLCULARIN MESLEKİ TÜKENMİŞLİK DÜZEYLERİ İLE İŞ DOYUMU DÜZEYLERİ ARASINDAKİ İLİŞKİNİN İNCELENMESİ	Nigde University Journal of Physical Education & Sport Sciences / Nigde Üniversitesi Beden Egitimi ve Spor Bilimleri Dergisi	No	Abstract and keywords
741	A. ÇEtİN	2020	EXAMINATION OF THE TABLE TENNIS PLAYERS' GENERAL SELFEFFICACY PERCEPTIONS AND SOURCES OF SPORTS MOTIVATION. / MASA TENİSİ OYUNCULARININ GENEL ÖZ YETERLİLİK ALGILARI VE SPOR MOTİVASYON KAYNAKLARININ İNCELENMESİ	SPORMETRE: The Journal of Physical Education & Sport Sciences / Beden Eğitimi ve Spor Bilimleri Dergisi	No	Abstract and keywords
742	F. Moen; R. A. Federici; F. Abrahamsen	2015	Examining possible Relationships between mindfulness, stress, school-and sport performances and athlete burnout	International Journal of Coaching Science	No	Abstract and keywords
743	K. M. Fisher; J. L. Etnier	2014	Examining the Time Course of Attention During Golf Putts of Two Different Lengths in Experienced Golfers	Journal of Applied Sport Psychology	No	Abstract and keywords
744	W. Junyeon; W. Shanshan; J. Hongqing; J. Carson Smith; P. Jungjun	2017	Executive Function and the P300 after Treadmill Exercise and Futsal in College Soccer Players	Sports (2075-4663)	No	Abstract and keywords
745	F. Alarcón; N. Ureña; A. Castillo; D. Martín; D. Cárdenas	2017	Executive functions predict expertise in basketball players	Revista de Psicologia del Deporte	No	Abstract and keywords
746	E. Renaghan; H. L.	2023	Exercise Cardiac Load and Autonomic	Journal of Functional	No	Abstract and

	Wittels; L. A. Feigenbaum; M. J. Wishon; S. Chong; E. D. Wittels; S. Hendricks; D. Hecocks; K. Bellamy; J. Girardi; S. Lee; T. Vo; S. M. McDonald; S. H. Wittels		Nervous System Recovery during In-Season Training: The Impact on Speed Deterioration in American Football Athletes	Morphology & Kinesiology		keywords
747	P. A. Davis; L. Davis; S. Wills; R. Appleby; A. Nieuwenhuys	2018	Exploring sledging" and interpersonal emotion-regulation strategies in professional cricket"	Sport Psychologist	No	Abstract and keywords
748	R. Paulauskas; M. Stumbras; D. Coutinho; B. Figueira	2022	Exploring the impact of the COVID-19 pandemic in Euroleague Basketball	Frontiers in Psychology	No	Abstract and keywords
749	L. J. Stephenson; S. G. Edwards; E. E. Howard; A. P. Bayliss	2018	Eyes that bind us: Gaze leading induces an implicit sense of agency	Cognition	No	Abstract and keywords
750	J. C. Marr; S. Thau	2014	Falling from great (and not-so-great) heights: How initial status position influences performance after status loss	Academy of Management Journal	No	Abstract and keywords
751	Ş. Kiziltoprak	2020	Fatigue and Recovery in Football. / Futbolda Yorgunluk ve Toparlanma	Spor Hekimligi Dergisi/Turkish Journal of Sports Medicine	No	Abstract and keywords
752	D. Marqués-Jiménez; J. Calleja-González; I. Arratibel; A. Delextrat; N. Terrados	2017	Fatigue and recovery in soccer: Evidence and challenge	Open Sports Sciences Journal	No	Abstract and keywords
753	J. C. M. Barte; A. Nieuwenhuys; S. A. E. Geurts; M. A. J. Kompier	2017	Fatigue experiences in competitive soccer: development during matches and the impact of general performance capacity	Fatigue: Biomedicine, Health and Behavior	No	Abstract and keywords
754	R. A. J. Mercer; J. L. Russell; L. C. McGuigan; A. J. Coutts; D. S. Strack; B. D. McLean	2023	Finding the Signal in the Noise - Interday Reliability and Seasonal Sensitivity of 84 Countermovement Jump Variables in Professional Basketball Players	Journal of Strength and Conditioning Research	No	Abstract and keywords
755	M. A. Nabli; N. B. Abdelkrim; I. Jabri; T. Batikh; C. Castagna; K. Chamari	2016	Fitness Field Tests' Correlation With Game Performance in U-19-Category Basketball Referees	International Journal of Sports Physiology & Performance	No	Abstract and keywords
756	L. Harper; S. Jones; R. Julian; R. Page	2021	Fixture congestion in professional football	Sport & Exercise Scientist	No	Abstract and keywords
757	M. Lacome; J. Piscione; J. P. Hager; C. Carling	2017	Fluctuations in running and skill-related performance in elite rugby union match-play	European Journal of Sport Science	No	Abstract and keywords
758	N. J. Duru	2019	For Fear of the Fans: An Argument for Holding Sports Teams Accountable for Fans' Post-Match Conduct	Texas Review of Entertainment & Sports Law	No	Abstract and keywords
759	A. M. Orozco; A. B. Vázquez	2021	Formative implications of the causal attribution of the result in school football	Retos	No	Abstract and keywords
760	S. Buzzelli	2021	From sigma test" to customized training"	Coaching & Sport Science Review	No	Abstract and keywords
761	T. J. Knight	2022	From the Field - Directed Topic: TACTICAL PERIODISATION AND FOOTBALL CONDITIONING: CONCEPT AND APPLICATION	Journal of Australian Strength & Conditioning	No	Abstract and keywords
762	N. Bonney; P. Larkin; K. Ball	2020	Future Directions and Considerations for Talent Identification in Australian Football	Frontiers in Sports and Active Living	No	Abstract and keywords
763	C. H. Almeida; P. Cruz; R. Gonçalves; R. Batalau; P. Paixão; J. A. Jorge; P.	2022	Game criticality in male youth football: Situational and age-related effects on the goal-scoring period in Portuguese national	Retos	No	Abstract and keywords

	Vargas		championships			
764	A. Carolina-Paludo; F. Nunes-Rabelo; M. Maciel-Batista; I. Rúbila-Maciel; M. Peikriszwili-Tartaruga; A. C. Simões	2020	Game location effect on pre-competition cortisol concentration and anxiety state: A case study in a futsal team	Revista de Psicologia del Deporte	No	Abstract and keywords
765	G. Ziv; R. Lidor	2021	Gaze Behavior in Golf Putting - A Review	International Journal of Golf Science	No	Abstract and keywords
766	C. M. Meira; C. O. Cortes; D. M. Corbetta; D. L. Oliveira; S. T. Rodrigues; B. V. F. Silva; M. Massa	2022	Gaze behaviour differentiates elite from non-elite female soccer players: a 2D video projections exploratory study	European Journal of Human Movement	No	Abstract and keywords
767	Y. Jacob; P. Chivers; R. S. Anderton	2019	Genetic predictors of match performance in sub-elite Australian football players: A pilot study	Journal of Exercise Science and Fitness	No	Abstract and keywords
768	F. A. Gutierrez; A. P. Sierra; E. D. Silva; V. C. Santos; M. P. Benetti; M. B. Silva; O. S. Gondim; H. H. Oliveira; C. M. Momesso; R. Gorjão; J. B. Pesquero; M. F. Cury-Boaventura	2020	Genetic variation, inflammatory and muscle injury response in rugby players to different positions in the field	Gazzetta Medica Italiana Archivio per le Scienze Mediche	No	Abstract and keywords
769	B. Min; I. Choi	2016	Heavy-heartedness biases your weight perception	Journal of Social Psychology	No	Abstract and keywords
770	E. Manci; O. C. Deniz; C. Guducu; E. Gunay; C. S. Bediz	2021	Hemodynamic changes in athletes' brains: is there any adaptation?	General Physiology and Biophysics	No	Abstract and keywords
771	L. Moscaleski; A. C. Paludo; V. L. Panissa; S. I. Delima; A. H. Okano; A. Moreira	2022	Home-based training program during the SARS-CoV-2 quarantine: training load, motivation, and wellbeing in professional elite female basketball players	Journal of Sports Medicine and Physical Fitness	No	Abstract and keywords
772	B. Holfelder; T. J. Klotzbier; M. Eisele; N. Schott	2020	Hot and Cool Executive Function in Elite- and Amateur- Adolescent Athletes From Open and Closed Skills Sports	Frontiers in Psychology	No	Abstract and keywords
773	E. Bisagno; S. Morra	2018	How do we learn to "kill" in volleyball?: The role of working memory capacity and expertise in volleyball motor learning	Journal of Experimental Child Psychology	No	Abstract and keywords
774	E. K. Coughlan; A. M. Williams; A. P. McRobert; P. R. Ford	2014	How experts practice: A novel test of deliberate practice theory	Journal of Experimental Psychology: Learning Memory and Cognition	No	Abstract and keywords
775	J. Ochoa-Lácar; M. Singh; S. P. Bird; J. Charest; T. Huyghe; J. Calleja-González	2022	How Sleep Affects Recovery and Performance in Basketball: A Systematic Review	Brain Sciences	No	Abstract and keywords
776	S. Pineda-Hernández	2022	How to play under pressure: EEG monitoring of mental activation training in a professional tennis player	Physiology and Behavior	No	Abstract and keywords
777	P. Fritz; R. Fritz; L. Mayer; B. Németh; J. Ressinka; P. Ács; C. Oláh	2022	Hungarian male water polo players' body composition can predict specific playing positions and highlight different nutritional needs for optimal sports performance	BMC Sports Science, Medicine & Rehabilitation	No	Abstract and keywords
778	S. Zhang; R. Roberts; T. Woodman; A. Cooke	2020	I Am Great, but Only When I Also Want to Dominate: Maladaptive Narcissism Moderates the Relationship Between Adaptive Narcissism and Performance Under Pressure	Journal of Sport & Exercise Psychology	No	Abstract and keywords

779	A. Alim; Y. Miftachurochmah	2023	Identification of Athlete Transfer Problematic Experiences toward the Social Adaptation Ability in Female Futsal Athletes	International Journal of Human Movement and Sports Sciences	No	Abstract and keywords
780	A. Kittel; P. Larkin; N. Elsworthy; M. Spittle	2019	Identification of key performance characteristics of elite Australian football umpires	International Journal of Sports Science & Coaching	No	Abstract and keywords
781	A. S. Kalani; S. Zardoshtian; S. Bahrami; M. Sadeghi	2021	Identifying and prioritizing the factors affecting the Iranian Sports Refereeing Ethics Code	Sport TK	No	Abstract and keywords
782	M. Bieleke; W. Wolff; C. Englert; P. M. Gollwitzer	2021	If-Then Planning in Sports: A Scoping Review	Zeitschrift fur Sportpsychologie	No	Abstract and keywords
783	F. García; J. Castellano; J. Vicens-Bordas; J. Vázquez-Guerrero; D. Ferioli	2023	Impact of a 6-Day Official Tournament on Physical Demands, Perceptual–Physiological Responses, Well-Being, and Game Performance of Under-18 Basketball Players	International Journal of Sports Physiology and Performance	No	Abstract and keywords
784	S. Takahashi; P. M. Grove	2023	Impact of acute open-skill exercise on inhibitory control and brain activation: A functional near-infrared spectroscopy study	PLoS ONE	No	Abstract and keywords
785	D. Suárez Iglesias; A. S. Leicht; H. Pojskić; A. Vaquera	2021	Impact of contextual factors on match demands experienced by elite male referees during international basketball tournaments	Journal of Sports Sciences	No	Abstract and keywords
786	V. J. Clemente-Suárez; S. Villafaina; T. García-Calvo; J. P. Fuentes-García	2022	Impact of HIIT Sessions with and without Cognitive Load on Cortical Arousal, Accuracy and Perceived Exertion in Amateur Tennis Players	Healthcare (Switzerland)	No	Abstract and keywords
787	K. CieŚLuk	2022	Impact of repeated short-term physical exercise on the level of selected technical skills in youth volleyball	Journal of Physical Education & Sport	No	Abstract and keywords
788	R. M. Ayuso-Moreno; J. P. Fuentes-García; H. Nobari; S. Villafaina	2021	Impact of the result of soccer matches on the heart rate variability of women soccer players	International Journal of Environmental Research and Public Health	No	Abstract and keywords
789	B. Oboudi; A. Elahi; H. Akbari Yazdi; D. Y. Pyun	2022	Impacts of game attractiveness and color of message on sport viewers' attention to prosocial message: an eye-tracking study	Sport, Business and Management: An International Journal	No	Abstract and keywords
790	R. D. Samuel; Y. Galily; E. Filho; G. Tenenbaum	2020	Implementation of the Video Assistant Referee (VAR) as a Career Change-Event: The Israeli Premier League Case Study	Frontiers in Psychology	No	Abstract and keywords
791	L. d. C. d. Silva; C. F. Pereira-Monfredini; L. A. Teixeira	2017	Improved children’s motor learning of the basketball free shooting pattern by associating subjective error estimation and extrinsic feedback	Journal of Sports Sciences	No	Abstract and keywords
792	S. Brini; L. P. Ardigò; F. M. Clemente; J. Raya-González; J. A. Kurtz; G. A. Casazza; C. Castagna; A. Bouassida; H. Nobari	2023	Increased game frequency period crossing Ramadan intermittent fasting decreases fat mass, sleep duration, and recovery in male professional basketball players	PeerJ	No	Abstract and keywords
793	K. Morris-Binelli; S. Müller; F. E. C. A. van Rens; A. G. Harbaugh; S. M. Rosalie	2021	Individual differences in performance and learning of visual anticipation in expert field hockey goalkeepers	Psychology of Sport and Exercise	No	Abstract and keywords
794	F. van Abswoude; N. B. Nuijen; J. van der Kamp; B. Steenbergen	2018	Individual Differences Influencing Immediate Effects of Internal and External Focus Instructions on Children's Motor Performance	Research Quarterly for Exercise & Sport	No	Abstract and keywords
795	G. M. Praça; R. B. e. Sousa; P. J. Greco	2019	Influence of Aerobic Power on Youth Players' Tactical Behavior and Network Properties during Football Small-Sided	Sports (2075-4663)	No	Abstract and keywords

			Games			
796	A. Lola; A. Koutsomarkou; G. Tzetzis	2022	Influence of different focus of attention instructions on learning volleyball skills for young novices	Journal of Human Sport and Exercise	No	Abstract and keywords
797	S. L. Moss; C. Twist	2015	Influence of different work and rest distributions on performance and fatigue during simulated team handball match play	Journal of Strength and Conditioning Research	No	Abstract and keywords
798	C. F. Wilke; S. P. Wanner; W. H. M. Santos; E. M. Penna; G. P. Ramos; F. Y. Nakamura; R. Duffield	2020	Influence of faster and slower recovery-profile classifications, self-reported sleep, acute training load, and phase of the microcycle on perceived recovery in futsal players	International Journal of Sports Physiology and Performance	No	Abstract and keywords
799	N. Dobbin; A. Atherton; C. Hill	2021	Influence of game design, physical demands, and skill involvement on the subjective task load associated with various small-sided games among elite junior rugby league players	International Journal of Sports Physiology and Performance	No	Abstract and keywords
800	R. Dubois; M. Lyons; T. Paillard; O. Maurelli; J. Prioux	2020	Influence of weekly workload on physical, biochemical and psychological characteristics in professional rugby union players over a competitive season	Journal of Strength and Conditioning Research	No	Abstract and keywords
801	L. W. Hogarth; B. J. Burkett; M. R. McKean	2015	Influence of Yo-Yo IR2 scores on internal and external workloads and fatigue responses of tag football players during tournament competition	PLoS ONE	No	Abstract and keywords
802	E. J. O'Connor; A. Murphy; M. J. Kohler; R. W. Chan; M. A. Immink	2022	Instantaneous effects of mindfulness meditation on tennis return performance in elite junior athletes completing an implicitly sequenced serve return task	Frontiers in Sports and Active Living	No	Abstract and keywords
803	R. Izzo; U. Rossini; G. Raiola; P. A. Cejudo; V. I. C. Hosseini	2020	Insurgence of fatigue and its implications in the selection and accuracy of passes in football. A case study	Journal of Physical Education & Sport	No	Abstract and keywords
804	J. E. Teixeira; P. Forte; R. Ferraz; L. Branquinho; A. J. Silva; A. M. Monteiro; T. M. Barbosa	2022	Integrating physical and tactical factors in football using positional data: a systematic review	PeerJ	No	Abstract and keywords
805	P. A. Dillon; T. Kempton; S. Ryan; J. Hocking; A. J. Coutts	2018	Interchange rotation factors and player characteristics influence physical and technical performance in professional Australian Rules football	Journal of Science and Medicine in Sport	No	Abstract and keywords
806	T. A. C. D. Oliveira; K. Davids; R. A. Denardi; S. Zalla; U. C. Corrêa	2023	Interpersonal coordination tendencies and perception of visual information for decision-making in futsal	Psychology of Sport and Exercise	No	Abstract and keywords
807	J. A. Jensen; D. Head; O. Monroe; S. Nestler	2022	Investigating sport league sponsor retention: Results from a semi-parametric hazard model	Sport Management Review	No	Abstract and keywords
808	G. Bastug	2018	Investigation of Attention, Concentration and Mental Toughness Properties in Tennis, Table Tennis, and Badminton Athletes	Sport Journal	No	Abstract and keywords
809	Y. ŞİRİN; E. DÖŞYilmaz	2017	INVESTIGATION OF JOB SATISFACTION AND BURNOUT LEVELS OF TURKISH SUPER LEAGUE FOOTBALL REFEREES. / TÜRKİYE SÜPER LİG FUTBOL HAKEMLERİNİN İŞ DOYUMU VE TÜKENMİŞLİK DÜZEYLERİNİNİNCELENMESİ	Nigde University Journal of Physical Education & Sport Sciences / Nigde Üniversitesi Beden Egitimi ve Spor Bilimleri Dergisi	No	Abstract and keywords
810	F. Bergmann; C. Meier; M. Braksiek	2022	Involvement and performance of U9 soccer players in 7v7 and 5v5 matches during	German Journal of Exercise and Sport Research	No	Abstract and keywords

			competition			
811	A. Neira; R. Silvestre; A. Debandi; D. Darras; I. Cristi-Sánchez; I. Barra; L. Peñailillo; C. De La Fuente	2022	Is the Rotatory Knee Stability Immediately Decreased Following a Competitive Soccer Match?	Frontiers in Bioengineering and Biotechnology	No	Abstract and keywords
812	E. Rio; M. Van Ark; S. Docking; G. L. Moseley; D. Kidgell; J. E. Gaida; I. Van Den Akker-Scheek; J. Zwerver; J. Cook	2017	Isometric contractions are more analgesic than isotonic contractions for patellar tendon pain: An in-season randomized clinical trial	Clinical Journal of Sport Medicine	No	Abstract and keywords
813	N. Bonney; P. Larkin; K. Ball	2022	Kick proficiency and skill adaptability increase from an Australian football small-sided game intervention	Frontiers in Sports and Active Living	No	Abstract and keywords
814	O. Estrada-Contreras; N. Fernández Martínez; E. Pérez-Córdoba; E. Cantón Chirivella; P. Jodra Jiménez; E. Huertas Castro	2023	La activación psicofisiológica en situaciones de competición en jugadores de bádminton. / The psychophysiological activation in competition situations in badminton players	Cuadernos de Psicología del Deporte	No	Abstract and keywords
815	M. Wigham; S. Wheatley	2023	Learn, Move, Compete: An alternative approach to mini tennis lessons	Coaching & Sport Science Review	No	Abstract and keywords
816	C. Porter; D. Greenwood; D. Panchuk; G.-J. Pepping	2020	Learner-adapted practice promotes skill transfer in unskilled adults learning the basketball set shot	European Journal of Sport Science	No	Abstract and keywords
817	J. B. Apidogo; J. Burdack; W. I. Schöllhorn	2022	Learning Multiple Movements in Parallel—Accurately and in Random Order, or Each with Added Noise?	International Journal of Environmental Research and Public Health	No	Abstract and keywords
818	S. J. Shrom; J. Cumming; S. J. Fenton	2023	Lifestyle challenges and mental health of professional tennis players: an exploratory case study	International Journal of Sport and Exercise Psychology	No	Abstract and keywords
819	J. L. Fox; A. T. Scanlan; R. Stanton; C. J. O'Grady; C. Sargent	2020	Losing Sleep Over It: Sleep in Basketball Players Affected by Game But Not Training Workloads	International Journal of Sports Physiology & Performance	No	Abstract and keywords
820	J. Petrović; D. Stanić; G. Dmitrašinović; B. Plećaš-Solarović; S. Ignjatović; B. Batinić; D. Popović; V. Pešić	2016	Magnesium supplementation diminishes peripheral blood lymphocyte DNA oxidative damage in athletes and sedentary young man	Oxidative Medicine and Cellular Longevity	No	Abstract and keywords
821	A. Shipnuck	2016	MAJOR CATASTROPHE	Sports Illustrated	No	Abstract and keywords
822	A. Karlinsky; N. J. Hodges	2019	Manipulations to practice organization of golf putting skills through interleaved matched or mismatched practice with a partner	Human Movement Science	No	Abstract and keywords
823	D. Wen; S. Robertson; G. Hu; B. Song; H. Chen	2018	Measurement properties and feasibility of the Loughborough soccer passing test: A systematic review	Journal of Sports Sciences	No	Abstract and keywords
824	J. L. Russell; B. D. McLean; F. M. Impellizzeri; D. S. Strack; A. J. Coutts	2021	Measuring Physical Demands in Basketball: An Explorative Systematic Review of Practices	Sports Medicine	No	Abstract and keywords
825	S. Mellalieu; C. Jones; C. Wagstaff; S. Kemp; M. J. Cross	2021	Measuring Psychological Load in Sport	Int J Sports Med	No	Abstract and keywords
826	M. R. Smith; S. M. Marcora; A. J. Coutts	2015	Mental Fatigue Impairs Intermittent Running Performance	MEDICINE AND SCIENCE IN SPORTS AND EXERCISE	No	Abstract and keywords

827	B. ŞİMŞEk; A. Kartal; S. AktaŞ	2023	Mental Toughness Levels of Male and Female Football Players. / Erkek ve Kadın Futbolcuların Zihinsel Dayanıklılık Düzeylerinin İncelenmesi	Turkish Journal of Sport & Exercise / Türk Spor & Egzersiz Dergisi	No	Abstract and keywords
828	C. F. Wilke; G. P. Ramos; D. A. S. Pacheco; W. H. M. Santos; M. S. L. Diniz; G. G. P. Gonçalves; J. C. B. Marins; S. P. Wanner; E. Silami-Garcia	2016	Metabolic Demand and Internal Training Load in Technical-Tactical Training Sessions of Professional Futsal Players	Journal of Strength and Conditioning Research	No	Abstract and keywords
829	J. V. García-Tormo; A. V. Jiménez; J. C. M. Rábago	2015	Methodological proposal for the quantification and analysis of the level of risk assumed in volleyball service execution in female high-level competition	Journal of Physical Education and Sport	No	Abstract and keywords
830	E. Bellomo; A. Cooke; G. Gallicchio; C. Ring; J. Hardy	2020	Mind and body: Psychophysiological profiles of instructional and motivational self-talk	Psychophysiology	No	Abstract and keywords
831	M. Pokolm; R. Rein; D. Müller; S. Nopp; M. Kirchhain; K. M. Aksum; G. Jordet; D. Memmert	2022	Modeling Players' Scanning Activity in Football	Journal of Sport & Exercise Psychology	No	Abstract and keywords
832	P. Fargier; S. Champely; R. Massarelli; L. Ammary; N. Hoyek	2022	Modelling response time in a mental rotation task by gender, physical activity, and task features	Scientific Reports	No	Abstract and keywords
833	A. D. Heishman; B. D. Daub; R. M. Miller; E. D. S. Freitas; M. G. Bemben	2020	Monitoring External Training Loads and Neuromuscular Performance for Division I Basketball Players over the Preseason	Journal of Sports Science & Medicine	No	Abstract and keywords
834	L. H. S. Fagundes; I. T. da Costa; C. P. Reis; G. De Sousa Pinheiro; V. T. Costa	2021	Monitoring of overtraining and motivation in elite soccer players	Motriz. Revista de Educacao Fisica	No	Abstract and keywords
835	F. N. Rabelo; B. N. Pasquarelli; B. Gonçalves; F. Matzenbacher; F. A. D. Campos; J. Sampaio; F. Y. Nakamura	2016	Monitoring the Intended and Perceived Training Load of a Professional Futsal Team Over 45 Weeks: A Case Study	Journal of Strength and Conditioning Research	No	Abstract and keywords
836	G. G. Bevilacqua; M. S. Viana; P. J. B. G. Filho; V. S. Borges; R. Brandt	2019	Mood states and sports results of a team throughout the second phase of the national futsal league	Psicologia: Teoria e Pesquisa	No	Abstract and keywords
837	F. Nutt; S. P. Hills; M. Russell; M. Waldron; P. Scott; J. Norris; C. J. Cook; B. Mason; N. Ball; L. P. Kilduff	2022	Morning resistance exercise and cricket-specific repeated sprinting each improve indices of afternoon physical and cognitive performance in professional male cricketers	Journal of Science and Medicine in Sport	No	Abstract and keywords
838	X. Yin; C. C. Vignesh; T. Vadivel	2022	Motion capture and evaluation system of football special teaching in colleges and universities based on deep learning	International Journal of System Assurance Engineering and Management	No	Abstract and keywords
839	S. DŽAjiĆ; D. Kuna	2017	MOTOR ACHIEVEMENTS DIFFERENCES IN BASKETBALL FOR JUNIOR HIGH SCHOOL STUDENTS. / RAZLIKE MOTORIČKIH DOSTIGNUĆA U KOŠARCI KOD UČENIKA OSNOVNE ŠKOLE	Sports Science & Health / Sportske Nauke i Zdravlje	No	Abstract and keywords
840	N. Jacobson; Q. Berleman-Paul; M. Mangalam; D. G. Kelty-Stephen; C. Ralston	2021	Multifractality in postural sway supports quiet eye training in aiming tasks: A study of golf putting	Human Movement Science	No	Abstract and keywords
841	A. Vu; A. Sorel; A.	2022	Multiple Players Tracking in Virtual Reality:	Frontiers in Psychology	No	Abstract and

	Limballe; B. Bideau; R. Kulpa		Influence of Soccer Specific Trajectories and Relationship With Gaze Activity			keywords
842	H. Bengtsson; J. Ekstrand; M. Waldén; M. Hägglund	2018	Muscle injury rate in professional football is higher in matches played within 5 days since the previous match: A 14-year prospective study with more than 130 000 match observations	British Journal of Sports Medicine	No	Abstract and keywords
843	H. Wiig; K. T. Cumming; V. Handegaard; J. Stabell; M. Spencer; T. Raastad	2022	Muscular heat shock protein response and muscle damage after semi-professional football match	Scandinavian Journal of Medicine & Science in Sports	No	Abstract and keywords
844	H. B. Cho; C. E. Bueler; J. Dimuzio; C. Hicks-Little; E. McGlade; I. K. Lyoo; D. Yurgelun-Todd	2018	Negative Mood States Correlate with Laterobasal Amygdala in Collegiate Football Players	BioMed Research International	No	Abstract and keywords
845	S. M. Mousavi; J. Dehghanizade; T. Iwatsuki	2022	Neither Too Easy Nor Too Difficult: Effects of Different Success Criteria on Motor Skill Acquisition in Children	Journal of Sport & Exercise Psychology	No	Abstract and keywords
846	J. Castellano; I. Echeazarra	2019	Network-based centrality measures and physical demands in football regarding player position: Is there a connection? A preliminary study	Journal of Sports Sciences	No	Abstract and keywords
847	S.-C. Kao; C.-J. Huang; T.-M. Hung	2014	Neurofeedback Training Reduces Frontal Midline Theta and Improves Putting Performance in Expert Golfers	Journal of Applied Sport Psychology	No	Abstract and keywords
848	L. T. Markwell; A. J. Strick; J. M. Porter	2022	No Fans, No Problem: An Investigation of Audience Effects on Shooting Performance in Professional Basketball	Journal of Motor Learning & Development	No	Abstract and keywords
849	M. E. d. S. Nunes; U. C. Correa; M. G. T. X. d. Souza; L. Basso; D. B. Coelho; S. Santos	2019	No Improvement on the Learning of Golf Putting By Older Persons With Self-Controlled Knowledge of Performance	Journal of Aging & Physical Activity	No	Abstract and keywords
850	A. Adda; D. Benamar	2023	Numerically equal small-sided game effect of 2v2 and 3v3 in a decision- making task simulated in handball	Journal of Physical Education & Sport	No	Abstract and keywords
851	B. A. McKay; J. A. Delaney; A. Simpkin; T. Larkin; A. Murray; D. Daniels; C. R. Pedlar; J. A. Sampson	2023	Objective Measures of Strain and Subjective Muscle Soreness Differ Between Positional Groups and Season Phases in American College Football	International Journal of Sports Physiology and Performance	No	Abstract and keywords
852	Y. L. Dickens; J. V. Raalte; R. T. Hurlburt	2018	On Investigating Self-Talk: A Descriptive Experience Sampling Study of Inner Experience During Golf Performance	Sport Psychologist	No	Abstract and keywords
853	G. Wood; G. Jordet; M. R. Wilson	2015	On winning the “lottery”: psychological preparation for football penalty shoot-outs	Journal of Sports Sciences	No	Abstract and keywords
854	R. A. Birrento; J. M. Giménez-Egido; E. Ortega	2023	OPINIÓN DE ENTRENADORES Y EXPERTOS SOBRE MODIFICACIONES REGLAMENTARIAS EN LA CATEGORÍA PREINFANTIL (UNDER-13) EN BALONCESTO. / COACHES AND EXPERTS' OPINION ABOUT BASKETBALL MODIFICATION RULES IN UNDER 13 LEVEL	Journal of Sport & Health Research	No	Abstract and keywords
855	M. D. Frick; M. D. Hamlet; F. Tudini; J. A. Bunn	2021	Original Scientific Research Study NO CORRELATION BETWEEN WELLNESS AND COUNTERMOVEMENT JUMP IN FEMALE COLLEGIATE LACROSSE	Journal of Australian Strength & Conditioning	No	Abstract and keywords
856	J. Jooste; A. N. Kubayi	2018	Perceived coach leadership style and psychological well-being among South	Disability and Health Journal	No	Abstract and keywords

			African national male wheelchair basketball players			
857	V. Beniscelli; G. Tenenbaum; R. J. Schinke; M. Torregrosa	2014	Perceived distributed effort in team ball sports	Journal of Sports Sciences	No	Abstract and keywords
858	B. J. Almagro; P. Sáenz-López; S. Fierro-Suero; C. Conde	2020	Perceived performance, intrinsic motivation and adherence in athletes	International Journal of Environmental Research and Public Health	No	Abstract and keywords
859	D. Conte; A. Tessitore; K. Smiley; C. Thomas; T. G. Favero	2016	Performance profile of NCAA Division I men's basketball games and training sessions	Biology of Sport	No	Abstract and keywords
860	T. Perri; R. Duffield; A. Murphy; T. Mabon; M. Reid	2023	Periodisation in professional tennis: A macro to micro analysis of load management strategies within a cluttered calendar	International Journal of Sports Science & Coaching	No	Abstract and keywords
861	A. F. Martin; S. P. Walker; L. A. McHugh	2023	PERSPECTIVE TAKING AS A PREDICTOR OF BURNOUT AMONG COMPETITIVE ADOLESCENT SQUASH PLAYERS	South African Journal for Research in Sport, Physical Education & Recreation	No	Abstract and keywords
862	P. Sansone; A. Tessitore; H. Paulauskas; I. Lukonaitiene; H. Tschan; V. Pliauga; D. Conte	2019	Physical and physiological demands and hormonal responses in basketball small-sided games with different tactical tasks and training regimes	Journal of Science and Medicine in Sport	No	Abstract and keywords
863	D. Supriadi; G. F. Friskawati; V. A. Karisman	2023	Physical Fitness of Futsal Athletes in Competition Preparation	International Journal of Human Movement and Sports Sciences	No	Abstract and keywords
864	J. A. Ovans; M. C. Hooke; A. E. Bendel; L. R. Tanner	2018	Physical Therapist Coaching to Improve Physical Activity in Children With Brain Tumors: A Pilot Study	PEDIATRIC PHYSICAL THERAPY	No	Abstract and keywords
865	S. Russell; M. J. Simpson; A. G. Evans; T. J. Coulter; V. G. Kelly	2021	Physiological and perceptual recovery-stress responses to an elite netball tournament	International Journal of Sports Physiology and Performance	No	Abstract and keywords
866	V. Loureiro da Silva; C. E. do Rosário Depizzol; L. Carletti; L. N. dos Santos Neves; R. Luís Vancini; C. A. Barbosa de Lira; M. dos Santos Andrade; A. Paula Lima-Leopoldo; A. Soares Leopoldo	2020	PHYSIOLOGICAL RESPONSES ASSOCIATED WITH CARDIOPULMONARY EXERCISE TESTING IN ELITE SOCCER REFEREES, DISTANCE RUNNERS AND HEALTH CONTROLS. / Respostas fisiológicas associadas ao teste de exercício cardiopulmonar em árbitros de futebol de elite, corredores de longa distância e controles de saúde	Revista Brasileira de Prescrição e Fisiologia do Exercício	No	Abstract and keywords
867	C. Ferrandez; T. Marsan; Y. Poulet; P. Rouch; P. Thoreux; C. Sauret	2021	Physiology, biomechanics and injuries in table tennis: A systematic review	Science and Sports	No	Abstract and keywords
868	H. Abaza; M. Saad	2016	PLAY POSITIONS AND LEFT VENTRICULAR MASS AND IT RELATIONSHIP WITH PHYSICAL VARIABLES FOR SOCCER PLAYERS	Ovidius University Annals, Series Physical Education & Sport/Science, Movement & Health	No	Abstract and keywords
869	N. Robin; L. Dominique; G. R. Coudevylle	2021	Playing tennis in hot environment: Applied strategies and new directions	Coaching & Sport Science Review	No	Abstract and keywords
870	H. Pineda	2022	Playing under pressure: EEG monitoring of activation in professional tennis players	Physiology and Behavior	No	Abstract and keywords
871	R. Abreu; P. Figueiredo; P. Beckert; J. P. Marques; S. Amorim; C. Caetano; P. Carvalho; C. Sá; R. Cotovio; J. Cruz; T. Dias; G. Fernandes; E.	2021	Portuguese Football Federation consensus statement 2020: Nutrition and performance in football	BMJ Open Sport and Exercise Medicine	No	Abstract and keywords

	Gonçalves; C. Leão; A. Leitão; J. Lopes; E. Machado; M. Neves; A. Oliveira; A. I. Pereira; B. Pereira; F. Ribeiro; L. M. Silva; F. Sousa; T. Tinoco; V. H. Teixeira; M. Sousa; J. Brito					
872	V. Sicard; G. Caron; R. D. Moore; D. Ellemberg	2021	Post-exercise cognitive testing to assess persisting alterations in athletes with a history of concussion	Brain Injury	No	Abstract and keywords
873	G. Ravier; P. Marcel-Millet; C. Fostel; E. Baradat	2022	Post-Exercise Cold- and Contrasting-Water Immersion Effects on Heart Rate Variability Recovery in International Handball Female Players	Journal of Human Kinetics	No	Abstract and keywords
874	M. Rico-González; J. Pino-Ortega; G. M. Praça; F. M. Clemente	2022	Practical Applications for Designing Soccer' Training Tasks From Multivariate Data Analysis: A Systematic Review Emphasizing Tactical Training	Perceptual and Motor Skills	No	Abstract and keywords
875	Z. Li; C. Yi; C. Chen; C. Liu; S. Zhang; S. Li; D. Gao; L. Cheng; X. Zhang; J. Sun; Y. He; P. Xu	2022	Predicting individual muscle fatigue tolerance by resting-state EEG brain network *	Journal of Neural Engineering	No	Abstract and keywords
876	K. E. Nolan; J. B. Caccese; A. P. Kontos; T. A. Buckley; G. G. P. Garcia; N. Port; S. P. Broglio; T. W. McAllister; M. McCrea; P. F. Pasquina; J. P. Hayes	2023	Primary and Secondary Risk Factors Associated With Concussion Symptom Clusters in Collegiate Athletes: Results From the NCAA-DoD Grand Alliance CARE Consortium	Orthopaedic Journal of Sports Medicine	No	Abstract and keywords
877	E. Herold; F. Boronczyk; C. Breuer	2021	Professional clubs as platforms in multi-sided markets in times of COVID-19: The role of spectators and atmosphere in live football	Sustainability (Switzerland)	No	Abstract and keywords
878	E. ÇAkaloĞLu; O. AkyÜZ; B. Sulu; P. Bayar	2019	PROFESYONEL FUTBOLCULARDA HEDEF YÖNELİMİNİN MOTİVASYONEL YÖNELİM ÜZERİNE ETKİSİ. / THE EFFECT OF GOAL ORIENTATION ON MOTIVATIONAL ORIENTATION IN PROFESSIONAL SOCCER PLAYERS	SPORMETRE: The Journal of Physical Education & Sport Sciences / Beden Eğitimi ve Spor Bilimleri Dergisi	No	Abstract and keywords
879	M. A. R. Alves; A. S. Oliveira; M. J. Paes; J. M. F. Stefanello	2022	Psychological aspects of soccer and futsal players: A systematic review	Suma Psicologica	No	Abstract and keywords
880	G. Jin	2021	Psychological Factors and Training Methods Affecting Chinese College Students' Confrontational Training in Football Teaching	Revista de Psicologia del Deporte	No	Abstract and keywords
881	F. R. De Almeida; C. B. Da Silva; C. M. Medeiros Vendramini; C. De Campos; M. R. Ferreira Brandão	2017	Psychometric properties of the brazilian version of the brums scale for basketball referees	Revista de Psicologia del Deporte	No	Abstract and keywords
882	M. S. Vaz; Y. S. Ribeiro; E. S. Pinheiro; F. B. Del Vecchio	2019	Psychophysiological profile and prediction equations for technical performance of football players	Revista Brasileira de Ciencias do Esporte	No	Abstract and keywords
883	J. Tallent; M. Higgins; N. Parker; M. Waldron; E.	2019	Quantification of bowling workload and changes in cognitive function in elite fast	Journal of Sports Medicine and Physical Fitness	No	Abstract and keywords

	Bradford; J. Keenan; B. V. O'Neill; P. G. Bell		bowlers in training compared with Twenty20 Cricket			
884	J. Vladovic; S. Versic; N. Foretic; R. Morgans; T. Modric	2023	Quantification of External Training Load among Elite-Level Goalkeepers within Competitive Microcycle	Applied Sciences (Switzerland)	No	Abstract and keywords
885	A. Dello Iacono; S. J. McLaren; T. W. Macpherson; M. Beato; M. Weston; V. B. Unnithan; T. Shushan	2023	Quantifying Exposure and Intra-Individual Reliability of High-Speed and Sprint Running During Sided-Games Training in Soccer Players: A Systematic Review and Meta-analysis	Sports Medicine	No	Abstract and keywords
886	D. Weaving; B. Jones; K. Till; P. Marshall; K. Earle; G. Abt	2020	Quantifying the External and Internal Loads of Professional Rugby League Training Modes: Consideration for Concurrent Field-Based Training Prescription	Journal of Strength and Conditioning Research	No	Abstract and keywords
887	Y. Hosokawa; S. O'Connor; C. Tashima; M. Otomo; A. Schmitt; E. Beidler	2023	Question Format Matters: Do Athletes Really Know the Signs and Symptoms of a Sport-Related Concussion?	Journal of Athletic Training (Allen Press)	No	Abstract and keywords
888	L. Dong; N. Berryman; T. Romeas	2023	Questioning the validity and reliability of using a video-based test to assess decision making among female and male water polo players	International Journal of Sports Science and Coaching	No	Abstract and keywords
889	H. Bougrine; A. Salem; N. Nasser; A. Ammar; H. Chtourou; N. Souissi	2023	Ramadan Fasting and Short-Term Maximal Physical Performance: Searching for Optimal Timing of the Last Meal "Suhoor" in Female Pre-University Handball Players	European Journal of Investigation in Health, Psychology and Education	No	Abstract and keywords
890	J. Calleja-Gonzalez; J. Mielgo-Ayuso; B. Sanchez-Ureña; S. M. Ostojic; N. Terrados	2019	Recovery in volleyball	Journal of Sports Medicine and Physical Fitness	No	Abstract and keywords
891	F. García; D. Fernández; J. Vázquez-Guerrero; R. Font; B. Moreno-Planas; D. Álamo-Arce; R. Medina-Ramírez; M. Mallol-Soler	2022	Recovery of the physiological status in professional basketball players using NESA neuromodulation treatment during different types of microcycles in season: A preliminary randomized clinical trial	Frontiers in Physiology	No	Abstract and keywords
892	A. A.-E; D. G	2018	Recovery strategies for football players	SSEM-Journal	No	Abstract and keywords
893	J. Calleja-González; A. Altarriba-Bartes; J. Mielgo-Ayuso; M. Casals; N. Terrados; J. Peña	2021	Recovery strategies for sports performance in the spanish professional basketball league (Acb)	Cultura, Ciencia y Deporte	No	Abstract and keywords
894	D. A. Yudkin; T. Rothmund; M. Twardawski; N. Thalla; J. J. Van Bavel	2016	Reflexive intergroup bias in third-party punishment	Journal of Experimental Psychology: General	No	Abstract and keywords
895	A. Vieira de Morais; F. C. de Andrade Nogueira; C. D. da Silva; M. G. Bara Filho	2023	Relação entre as Cargas de Treinamento, Recuperação e Burnout em Atletas de Voleibol. / Relationship between Training Load, Recovery and Burnout in Volleyball Athletes	Educación Física y Ciencia	No	Abstract and keywords
896	E. Lundkvist; H. Gustafsson; G. Björklund; P. Davis; A. Ivarsson	2021	Relating Competitive Golfers' Perceived Emotions and Performance	Perceptual & Motor Skills	No	Abstract and keywords
897	T. Li; Y. Ma	2023	RELATIONS BETWEEN PSYCHOLOGICAL QUALITY AND TRAINING IN BASKETBALL PLAYERS	Revista Brasileira de Medicina do Esporte	No	Abstract and keywords
898	R. F. García; F. Z. Ortega;	2014	Relationship between anxiety and	Universitas Psychologica	No	Abstract and

	D. L. Girela; J. A. Sandoval; F. P. de la Fuente; M. L. Manrique		self-esteem, field position and development of physical injuries			keywords
899	N. Tinkler; A. Kruger; J. Jooste	2021	RELATIONSHIP BETWEEN EMOTIONAL INTELLIGENCE AND COMPONENTS OF COMPETITIVE STATE ANXIETY AMONG SOUTH AFRICAN FEMALE FIELD-HOCKEY PLAYERS	South African Journal for Research in Sport, Physical Education & Recreation	No	Abstract and keywords
900	D. García-Santos; J. Pino-Ortega; J. García-Rubio; A. Vaquera; S. J. Ibáñez	2022	RELATIONSHIP BETWEEN EXTERNAL AND INTERNAL LOAD IN BASKETBALL REFEREES	Revista Internacional de Medicina y Ciencias de la Actividad Fisica y del Deporte	No	Abstract and keywords
901	A. R. Jagim; G. A. Wright; C. L. Camic; J. N. Kisiolek; J. Luedke; J. M. Oliver; K. M. Fischer; M. T. Jones	2021	Relationship between training load and recovery in collegiate American football players during pre-season training	Science & Medicine in Football	No	Abstract and keywords
902	J. Jaworski; G. Lech; M. Żak; K. Witkowski; P. Piepiora	2023	Relationships between selected indices of postural stability and sports performance in elite badminton players: Pilot study	Frontiers in Psychology	No	Abstract and keywords
903	F. Dervent; A. Kurucan; E. Devrilmez; P. Ward	2022	Relationships between university instructors' and preservice teachers' content knowledge	European Physical Education Review	No	Abstract and keywords
904	Y. Hidayat; Y. Yudiana; B. Hambali; R. Nugraha	2022	Reliability and Factorial Validity of Badminton Basic Skill among Badminton Beginner Athletes: A Preliminary Study	International Journal of Human Movement and Sports Sciences	No	Abstract and keywords
905	G. Taheri Karami; M. Hemmatinafar; M. Koushkie Jahromi; J. Nemati; A. Niknam	2023	Repeated mouth rinsing of coffee improves the specific-endurance performance and jump performance of young male futsal players	Journal of the International Society of Sports Nutrition	No	Abstract and keywords
906	J. Yin; M. Chen; Y. Ge; Q. Song; H. Zheng	2022	Research on Training Model of Volleyball Based on Flexible Strain Sensing Network for Training	Journal of Sensors	No	Abstract and keywords
907	M. CamÕEs; R. U. I. Silva; D. Oliveira; T. Sousa; P. Bezerra; R. Lima; F. M. Clemente	2022	RINK HOCKEY TEAM PERFORMANCE AND TECHNICAL DETERMINANTS OF THE GAME: A FULL-SEASON ANALYSIS	Human Movement	No	Abstract and keywords
908	T. Allen; M. Taberner; M. Zhilkin; D. Rhodes	2023	Running more than before? The evolution of running load demands in the English Premier League	International Journal of Sports Science and Coaching	No	Abstract and keywords
909	L. Pavlović; I. Bojić; N. Stojiljković; D. Djordjević; D. Radovanović	2018	Seasonal changes in selected physical and physiological variables in male handball players	Acta Facultatis Medicae Naissensis	No	Abstract and keywords
910	T. Ferreira Dias Kanthack; A. Guillot; L. Ricardo Altimari; S. Nunez Nagy; C. Collet; F. Di Rienzo	2016	Selective efficacy of static and dynamic imagery in different states of physical fatigue	PLoS ONE	No	Abstract and keywords
911	W. Selmi; H. Rebai; M. Chtara; A. Naceur; S. Sahli	2018	Self-confidence and affect responses to short-term sprint interval training	Physiology and Behavior	No	Abstract and keywords
912	A. Kaplánová	2019	Self-esteem, anxiety and coping strategies to manage stress in ice hockey	Acta Gymnica	No	Abstract and keywords
913	J. Nemček; D. Nemček	2022	SELF-PERCEIVED FATIGUE AFTER MOTOR ABILITIES TESTING IN ADOLESCENT ELITE TENNIS PLAYERS	Acta Facultatis Educationis Physicae Universitatis Comenianae	No	Abstract and keywords
914	F. Earle; B. Hockey; K. Earle; P. Clough	2015	Separating the effects of task load and task motivation on the effort-fatigue relationship	MOTIVATION AND EMOTION	No	Abstract and keywords

915	S. Lucia; M. Aydin; F. Di Russo	2023	Sex Differences in Cognitive-Motor Dual-Task Training Effects and in Brain Processing of Semi-Elite Basketball Players	Brain Sciences	No	Abstract and keywords
916	M.-Á. Gómez; S. Avugos; M.-Á. Oñoro; A. Lorenzo; M. Bar-Eli	2018	Shaq is Not Alone: Free-Throws in the Final Moments of a Basketball Game	Journal of Human Kinetics	No	Abstract and keywords
917	M. Herold; M. Kempe; L. Ruf; L. Guevara; T. Meyer	2022	Shortcomings of applying data science to improve professional football performance: Takeaways from a pilot intervention study	Frontiers in Sports and Active Living	No	Abstract and keywords
918	A. Liu	2021	Simulation of basketball motion image resolution based on FPGA and gaussian mixture model	Microprocessors and Microsystems	No	Abstract and keywords
919	R. Nehme; F. M. S. De Branco; P. F. Vieira; A. V. C. Guimaraes; G. K. Gomes; G. P. Teixeira; P. H. Rodrigues; L. M. De Castro Junior; G. M. Puga; B. Saunders; E. P. De Oliveira	2022	Single and Serial Carbohydrate Mouth Rinsing Do Not Improve Yo-Yo Intermittent Recovery Test Performance in Soccer Players	International Journal of Sport Nutrition and Exercise Metabolism	No	Abstract and keywords
920	J. Sarajärvi; R. Freitas; M. Elovaara; A. Volossovitch	2023	Skill-related studies from youth to high-performance football: a scoping review	German Journal of Exercise and Sport Research	No	Abstract and keywords
921	A. F. Silva; R. Oliveira; Z. Akyildiz; M. Yıldız; Y. Ocak; M. Günay; H. Sarmento; A. Marques; G. Badicu; F. M. Clemente	2022	Sleep Quality and Training Intensity in Soccer Players: Exploring Weekly Variations and Relationships	Applied Sciences (Switzerland)	No	Abstract and keywords
922	A. Andrade; G. G. Bevilacqua; D. R. Coimbra; F. S. Pereira; R. Brandt	2016	Sleep quality, mood and performance: A study of elite Brazilian volleyball athletes	Journal of Sports Science and Medicine	No	Abstract and keywords
923	L. D. A. Freire; P. Merino-Muñoz; E. Aedo-Muñoz; D. A. S. Soto; C. J. Brito; B. Miarka	2022	Soccer pacing strategy: chronological intracomparison of the same soccer athletes, disputing with the same opponent during the same year	Journal of Physical Education and Sport	No	Abstract and keywords
924	I. Teoldo; E. Mezzadri; F. Cardoso; G. Machado	2023	Speed of decision-making as a key element for professional and academy soccer players' performances	Retos	No	Abstract and keywords
925	K. Bahdur; L. Pillay; D. Dell'Oca	2022	Sport during the COVID-19 bio-bubble: Wellness and opinions in South African elite football	South African Journal of Sports Medicine	No	Abstract and keywords
926	R. Ballester; F. Huertas; E. Molina; D. Sanabria	2018	Sport participation and vigilance in children: Influence of different sport expertise	Journal of Sport and Health Science	No	Abstract and keywords
927	Y. Li; M. Zhao; Y. Cao; Y. Gao; Y. Wang; B. Yun; L. Luo; W. Liu; C. Zheng	2023	Static and dynamic resting-state brain activity patterns of table tennis players in 7-Tesla MRI	Frontiers in Neuroscience	No	Abstract and keywords
928	E. Galanis; A. Hatzigeorgiadis; F. Charachousi; A. T. Latinjak; N. Comoutos; Y. Theodorakis	2022	Strategic Self-Talk Assists Basketball Free Throw Performance Under Conditions of Physical Exertion	Frontiers in Sports and Active Living	No	Abstract and keywords
929	J. H. Tanimaru; A. N. A. L. P. Dos Santos	2016	Study on the quality of life at a baseball performance center	Journal of Physical Education & Sport	No	Abstract and keywords
930	M. A. Gabriela	2017	Study Regarding the Types of Incidence of Lower Limb Injuries in Romanian Badminton Players	Gymnasium: Scientific Journal of Education, Sports & Health	No	Abstract and keywords
931	G. B. Wilkerson; M. A.	2023	Subtle impairments of perceptual-motor	Frontiers in Sports and Active	No	Abstract and

	Colston; S. N. Acocello; J. A. Hogg; L. M. Carlson		function and well-being are detectable among military cadets and college athletes with self-reported history of concussion	Living		keywords
932	D. García-Santos; M. A. Gómez-Ruano; A. Vaquera; S. J. Ibáñez	2020	Systematic review of basketball referees' performances	International Journal of Performance Analysis in Sport	No	Abstract and keywords
933	R. Sánchez-López; I. Echeazarra; J. Castellano	2022	Systematic review of declarative tactical knowledge evaluation tools based on game-play scenarios in soccer	Quality and Quantity	No	Abstract and keywords
934	S. Schaefer; G. Amico	2022	Table tennis expertise influences dual-task costs in timed and self-initiated tasks	Acta Psychologica	No	Abstract and keywords
935	K. A. M. Sanusi; D. Di Mitri; B. Limbu; R. Klemke	2021	Table tennis tutor: Forehand strokes classification based on multimodal data and neural networks	Sensors	No	Abstract and keywords
936	L. Ribeiro; L. Figueiredo; J. PÉRez-Morales; G. Nascimento; D. Porto; P. Greco	2021	Tactical knowledge and visual search analysis of female handball athletes from different age groups	Journal of Physical Education & Sport	No	Abstract and keywords
937	R. Matos; C. Moreira; E. Alves; J. E. Teixeira; F. Rodrigues; D. Monteiro; R. Antunes; P. Forte	2023	Tactical Knowledge by Decision Making and Motor Efficiency of Young Football Players in Different Playing Positions during a Three-a-Side Small-Sided Game	Behavioral Sciences	No	Abstract and keywords
938	T. S. Duarte; D. L. Alves; D. R. Coimbra; B. Miloski; J. C. Bouzas Marins; M. G. Bara Filho	2019	Technical and Tactical Training Load in Professional Volleyball Players	International Journal of Sports Physiology & Performance	No	Abstract and keywords
939	S. Hendricks; T. van Niekerk; D. W. Sin; M. Lambert; S. den Hollander; J. Brown; W. Maree; P. Treu; K. Till; B. Jones	2018	Technical determinants of tackle and ruck performance in International rugby union	Journal of Sports Sciences	No	Abstract and keywords
940	G. de Sousa Pinheiro; D. Campos; V. Teoldo da Costa	2023	Temporal goal scoring characteristics in elite Brazilian football: a longitudinal study. / Características temporales de la anotación de goles en el fútbol de élite brasileño: un estudio longitudinal	Educación Física y Ciencia	No	Abstract and keywords
941	A. Fikri; R. R. Pratama; A. Haqiyah; A. Ramadhan; B. Hardiyono; A. Hidayat	2022	Tennis Ball Exersice: Variation to Increase Arm Muscle Strength in Martial Athletes at Sriwijaya State Sports School	International Journal of Human Movement and Sports Sciences	No	Abstract and keywords
942	L. D. Harper; R. Hunter; P. Parker; S. Goodall; K. Thomas; G. Howatson; D. J. West; E. Stevenson; M. Russell	2016	Test-Retest Reliability of Physiological and Performance Responses to 120 Minutes of Simulated Soccer Match Play	Journal of Strength and Conditioning Research	No	Abstract and keywords
943	E. Mokou; P. T. Nikolaidis; J. Padulo; N. Apostolidis	2016	The acute effect of exercise intensity on free throws in young basketball players	Sport Sciences for Health	No	Abstract and keywords
944	T. L. Chu	2015	The Application of Augmented Feedback in Coaching Table Tennis Youth Athletes	International Table Tennis Federation Sports Science Congress Conference Proceedings	No	Abstract and keywords
945	M. Marvier; P. Kareiva; D. Felix; B. J. Ferrante; M. B. Billington	2023	The benefits of nature exposure: The need for research that better informs implementation	Proceedings of the National Academy of Sciences of the United States of America	No	Abstract and keywords
946	J. J. Nam; D. H. Han	2020	The comparison of perfectionism and commitment between professional and amateur golfers and the association between	International Journal of Environmental Research and Public Health	No	Abstract and keywords

			perfectionism and commitment in the two groups			
947	M. H. White Ii; K. M. Sheldon	2014	The contract year syndrome in the NBA and MLB: A classic undermining pattern	Motivation and Emotion	No	Abstract and keywords
948	A. Field; R. J. Naughton; M. Haines; S. Lui; L. D. Corr; M. Russell; R. M. Page; L. D. Harper	2022	The demands of the extra-time period of soccer: A systematic review	Journal of Sport and Health Science	No	Abstract and keywords
949	J. A. Fleming; A. Field; S. Lui; R. J. Naughton; L. D. Harper	2023	The demands of training and match-play on elite and highly trained junior tennis players: A systematic review	International Journal of Sports Science & Coaching	No	Abstract and keywords
950	Georgiykorobeynikov; Gennadiilisenchuk; V. Tyshchenko; Tetianaodynets; Vasylchukvolodymyr; I. Dyadechko; Olenabessarabova; L. I. A. Galchenko; Pavlopyptiuk	2019	The dependence of emotional burnout on ovarian-menstrual cycle phases	Journal of Physical Education & Sport	No	Abstract and keywords
951	S. A. R. A. H. Al Hussaini; M. Abdel-Reda; K. M. Reda; H. N. H. Al-Shawi	2020	The effect of a mentoring program on competition orientation for football school athletes	Indian Journal of Forensic Medicine and Toxicology	No	Abstract and keywords
952	F. Moen; S. A. Pettersen; K. Gjertsås; M. Vatn; M. Ravenhorst; A. Kvålsvoll; K. H. Liland; E. F. Mosleth	2023	The effect of bio-electro-magnetic-energy-regulation therapy on sleep duration and sleep quality among elite players in Norwegian women's football	Frontiers in Psychology	No	Abstract and keywords
953	W. Sunarto; S. Nugroho; C. N. Wali; M. J. H. Louk	2023	The Effect of Circuit Training on Improving the Physical Condition of Northwest Pantar Football Athletes	International Journal of Human Movement and Sports Sciences	No	Abstract and keywords
954	Z. A. Fitrian; A. S. Graha; A. Nasrulloh; A. Munir; M. Asmara; N. Y. Irsyad	2023	The effect of circuit training, fartlek, and small-sided games on maximum oxygen consumption capacity building in futsal players	Health, Sport, Rehabilitation	No	Abstract and keywords
955	G. Rekik; G. Jouira; Y. Belkhir; M. Jarraya; C. D. Kuo; Y. S. Chen	2023	The effect of dynamic versus static visualizations on acquisition of basketball game actions: a diurnal study	Scientific Reports	No	Abstract and keywords
956	J. Tangkudung; M. Asmawi; A. S. Lengkana; A. A. Rahman; R. Abdulgani; D. Mulyana; B. Badaru	2021	The effect of endurance, eye-hand coordination, and confidence to volleyball referee performance in West Java	International Journal of Human Movement and Sports Sciences	No	Abstract and keywords
957	M. M. Wdowski; N. Clarke; E. L. J. Eyre; R. Morris; M. Noon; S. J. Eustace; J. Hankey; L. M. Raymond; D. L. Richardson	2021	The effect of fatigue on first stance phase kinetics during acceleration sprint running in professional football players	Science & Medicine in Football	No	Abstract and keywords
958	L. Feng; T. Rupčić; D. Knjaz	2021	THE EFFECT OF FATIGUE ON KINEMATICS AND KINETICS OF BASKETBALL DRIBBLING WITH CHANGES OF DIRECTION	Kinesiology	No	Abstract and keywords
959	M. Jalalvand; A. Bahram; A. Daneshfar; S. Arsham	2019	The Effect of Gradual Self-Control of Task Difficulty and Feedback on Learning Golf Putting	Research Quarterly for Exercise & Sport	No	Abstract and keywords
960	T. Jasinovic; J. S. Burma; B. Cameron; V. Lun; C. R.	2021	The effect of high-intensity physical exertion on measures of cervical spine,	Physical Therapy in Sport	No	Abstract and keywords

	van Rassel; B. Sutter; J. P. Wiley; K. J. Schneider		vestibular/ocular-motor screening, and vestibulo-ocular reflex function in university level collision and combative sport athletes			
961	Z. M. Khalaf	2023	The effect of light stimulation technology training on brain signals, motor response time, and jump shot accuracy among young basketball players	Sport TK	No	Abstract and keywords
962	H. T. Wang; Y. S. Chen; G. Rekik; C. C. Yang; M. S. Lai; H. L. Tai	2022	The effect of listening to preferred music after a stressful task on performance and psychophysiological responses in collegiate golfers	PeerJ	No	Abstract and keywords
963	Y. Wang; S. M. Lei; C. C. Wu	2023	The Effect of Mindfulness Intervention on the Psychological Skills and Shooting Performances in Male Collegiate Basketball Athletes in Macau: A Quasi-Experimental Study	International Journal of Environmental Research and Public Health	No	Abstract and keywords
964	J. Moradi; M. Maleki; H. Moradi	2023	The Effect of Part and Whole Practice on Learning Lay-Up Shot Skill in Young and Adolescent Male Students	Journal of Motor Learning and Development	No	Abstract and keywords
965	D. Davidow; M. Redman; M. Lambert; N. Burger; M. Smith; B. Jones; S. Hendricks	2020	The effect of physical fatigue on tackling technique in Rugby Union	Journal of Science and Medicine in Sport	No	Abstract and keywords
966	M. S. J. Al-Khuzai; H. F. Hasan	2020	The effect of special exercises on the most superior mental processes and the performance of some offensive skills for table tennis beginners	Indian Journal of Forensic Medicine and Toxicology	No	Abstract and keywords
967	A. Beavan; L. Hanke; J. Spielmann; S. Skorski; J. Mayer; T. Meyer; J. Fransen	2021	The effect of stroboscopic vision on performance in a football specific assessment	Science & Medicine in Football	No	Abstract and keywords
968	P. S. L. Buñuel; C. V. Domínguez; M. Á. G. Ruano; J. A. R. González	2021	The effect of teammates support on player's free-throws effectiveness	Revista de Psicologia del Deporte	No	Abstract and keywords
969	E. Efek; H. Eryigit	2022	THE EFFECT OF TENNIS EXERCISE PARTICIPATION ON STRESS LEVEL OF SECONDARY STUDENTS. / VPLIV UDELEŽBE PRI TENIŠKI VADBI NA RAVEN STRESA SREDNJEŠOLCEV	Kinesiologia Slovenica	No	Abstract and keywords
970	M. Ben Brahim; A. Sal-De-Rellán; A. García-Valverde; H. Yasin; J. Raya-González	2023	The effect of three different pre-match warm-up structures on male professional soccer players' physical fitness	PeerJ	No	Abstract and keywords
971	G. Zaharia; D. Badau; V. Tudor; R. Costache; A. Geambasu; M. Damian; L. Giurgiu; C. Damian; V. E. Ursu; R. G. Rusu; I. T. Hasmasan; I. Stoian; C. Tifrea	2023	The Effects of 8 Aerobic Endurance Training Weeks of 4vs.4+GK Small-Sided Games versus Traditional Training on Physical Fitness and Skills among U18 Football Players	Applied Sciences (Switzerland)	No	Abstract and keywords
972	R. Gorgulu; E. Gokcek	2021	The Effects of Avoiding Instructions Under Pressure: An Examination of the Volleyball Serving Task	Journal of Human Kinetics	No	Abstract and keywords
973	A. M. Ehlert; H. M. Twiddy; P. B. Wilson	2020	The effects of caffeine mouth rinsing on exercise performance: A systematic review	International Journal of Sport Nutrition and Exercise Metabolism	No	Abstract and keywords
974	D. Pavlů; A. Škripková; D. Pánek	2021	The effects of elastic band exercises with pnf on shooting speed and accuracy in ball	International Journal of Environmental Research and	No	Abstract and keywords

			hockey players during the covid-19 pandemic	Public Health		
975	D. Alder; P. R. Ford; J. Causer; A. M. Williams	2016	The Effects of High- and Low-Anxiety Training on the Anticipation Judgments of Elite Performers	Journal of Sport & Exercise Psychology	No	Abstract and keywords
976	P. Chmura; J. Chmura; W. Chodor; A. Drożdżowski; A. Rokita; M. Konefał	2023	The effects of high-intensity interval training at the anaerobic and psychomotor fatigue thresholds on physiological parameters in young soccer players: a prospective study	Frontiers in Physiology	No	Abstract and keywords
977	Y. Sarig; M. C. Ruiz; A. Hatzigeorgiadis; G. Tenenbaum	2023	The Effects of Instructional Self-Talk on Quiet-Eye Duration and Golf-Putting Performance	Sport Psychologist	No	Abstract and keywords
978	F. M. Clemente; J. Afonso; D. Castillo; A. L. Arcos; A. F. Silva; H. Sarmento	2020	The effects of small-sided soccer games on tactical behavior and collective dynamics: A systematic review	Chaos, Solitons and Fractals	No	Abstract and keywords
979	F. M. Clemente; H. Sarmento	2020	The effects of small-sided soccer games on technical actions and skills: A systematic review	Human Movement	No	Abstract and keywords
980	A. Green; C. Dafkin; S. Kerr; W. McKinon	2015	The effects of walking on golf drive performance in two groups of golfers with different skill levels	Biology of Exercise	No	Abstract and keywords
981	E. A. Harrison	2014	The First Concussion Crisis	American Journal of Public Health	No	Abstract and keywords
982	J. M. Lynch; M. Anderson; B. Benton; S. S. Green	2015	The Gaming of Concussions: A Unique Intervention in Postconcussion Syndrome	Journal of Athletic Training (Allen Press)	No	Abstract and keywords
983	S. Park; I. Yuhei	2018	The Impact of Brand-Event Fit in Virtual Advertising on Sport Television Viewers' Brand Attitudes	Sport Marketing Quarterly	No	Abstract and keywords
984	I. Senécal; S. J. Howarth; G. D. Wells; I. Raymond; S. Mior	2021	The Impact of Moderate and High Intensity Cardiovascular Exertion on Sub-Elite Soccer Referee's Cognitive Performance: A Lab-Based Study	Journal of Sports Science & Medicine	No	Abstract and keywords
985	S. J. Ibáñez; J. A. Pérez-Goye; J. Courel-Ibáñez; J. García-Rubio	2018	The impact of scoring first on match outcome in women’s professional football	International Journal of Performance Analysis in Sport	No	Abstract and keywords
986	M. Turner; P. Beranek; S. Sahrom; J. Lo; A. Ferrauti; I. C. Dunican; T. Cruickshank	2023	The impact of sleep behaviours, chronotype and time of match on the internal and external outcomes of a tennis match	International Journal of Sports Science & Coaching	No	Abstract and keywords
987	A. K. Awad; Z. H. Amr	2023	The impact of the five-cycle learning strategy using some mental maps in learning the skill of reception and preparation in volleyball	Journal of Human Sport & Exercise	No	Abstract and keywords
988	L. D. Harper; E. J. Stevenson; I. Rollo; M. Russell	2017	The influence of a 12% carbohydrate-electrolyte beverage on self-paced soccer-specific exercise performance	Journal of Science and Medicine in Sport	No	Abstract and keywords
989	E. Mancı; F. Herold; E. Günay; Ç. Güdücü; N. G. Müller; C. Ş. Bediz	2023	The Influence of Acute Sprint Interval Training on the Cognitive Performance of Male Basketball Players: An Investigation of Expertise-Related Differences	International Journal of Environmental Research and Public Health	No	Abstract and keywords
990	S. Zasso; G. Decarli; F. Vitali; L. Franchin	2023	The influence of coaches’ emotions on the emotional state and performance of young tennis athletes	Giornale Italiano di Psicologia	No	Abstract and keywords
991	J. Arboix-Alió; G. Trabal;	2021	The influence of contextual variables on	International Journal of	No	Abstract and

	J. Valente-Dos-Santos; J. Aguilera-Castells; A. Fort-Vanmeerhaeghe; B. Buscà		individual set-pieces in elite rink hockey	Performance Analysis in Sport		keywords
992	T. Sousa; H. Sarmento; A. Marques; A. Field; V. Vaz	2020	The influence of opponents' offensive play on the performance of professional rink hockey goalkeepers	International Journal of Performance Analysis in Sport	No	Abstract and keywords
993	M. Turner; J. Lo; P. Beranek; I. C. Dunican; T. Cruickshank	2022	The influence of self-reported total sleep time and sleep quality on physical performance in junior tennis players. / La influencia del tiempo de sueño total autorreportado y la calidad del sueño en el desempeño físico de jugadores júnior de tenis	International Journal of Racket Sports Science	No	Abstract and keywords
994	F. R. Serpiello; A. Cox; L. Oppici; W. G. Hopkins; M. C. Varley	2017	The Loughborough Soccer Passing Test has impractical criterion validity in elite youth football	Science and Medicine in Football	No	Abstract and keywords
995	S. Duica; R. Balázsi; R. Ciulei; A. Bivolaru	2014	The mediating role of coping strategies between achievement goals and competitive anxiety in elite sport: A path analytic study	Cognition, Brain, Behavior	No	Abstract and keywords
996	D. H. Kwak	2016	The Overestimation Phenomenon in a Skill-Based Gaming Context: The Case of March Madness Pools	Journal of Gambling Studies	No	Abstract and keywords
997	R. Vavassori; M. P. Moreno; A. Ureña Espa	2023	The Perception of Volleyball Student-Athletes: Evaluation of Well-Being, Sport Workload, Players' Response, and Academic Demands	Healthcare (Switzerland)	No	Abstract and keywords
998	T. Mullen; C. Twist; J. Highton	2021	The physiological and perceptual effects of stochastic simulated rugby league match play	International Journal of Sports Physiology and Performance	No	Abstract and keywords
999	B. Low; R. Rein; D. Raabe; S. Schwab; D. Memmert	2021	The porous high-press? An experimental approach investigating tactical behaviours from two pressing strategies in football	Journal of Sports Sciences	No	Abstract and keywords
1000	Z. A. Fitrian; A. S. Graha; A. Nasrulloh; M. Asmara	2023	The Positive Impact of Small-Sided Games Training on VO2 max and Passing Accuracy in Futsal Players	International Journal of Human Movement and Sports Sciences	No	Abstract and keywords
1001	J. Tallis; N. Clarke; R. Morris; D. Richardson; M. Ellis; E. Eyre; M. Duncan; M. Noon	2021	The prevalence and practices of caffeine use as an ergogenic aid in English professional soccer	Biology of Sport	No	Abstract and keywords
1002	G. Martinent; V. Cece; M. T. Elferink-Gemser; I. R. Faber; J.-C. Decret	2018	The prognostic relevance of psychological factors with regard to participation and success in table-tennis	Journal of Sports Sciences	No	Abstract and keywords
1003	D. W. Eccles; A. W. Kazmier	2019	The psychology of rest in athletes: An empirical study and initial model	Psychology of Sport and Exercise	No	Abstract and keywords
1004	O. Estrada-Contreras; N. F. Martínez; E. Pérez-Córdoba; E. C. Chirivella; P. J. Jiménez; E. H. Castro	2023	The psychophysiological activation in competition situations in badminton players	Cuadernos de Psicologia del Deporte	No	Abstract and keywords
1005	G. Machado; S. González-Víllora; I. Teoldo	2023	The relationship between deliberate practice, play, and futsal in childhood and adolescence and the development of different decision-making skills in professional female soccer players	Psychology of Sport and Exercise	No	Abstract and keywords
1006	S. E. Short; M. W. Short; C. R. Haugen	2015	The Relationship Between Efficacy and Burnout in Coaches	International Journal of Coaching Science	No	Abstract and keywords
1007	R. Hardin; R. Zakrajsek; B. Gaston	2015	THE RELATIONSHIP BETWEEN JOB SATISFACTION AND BURNOUT IN	Journal of Contemporary Athletics	No	Abstract and keywords

			FAST-PITCH SOFTBALL COACHES			
1008	C. L. Oxendale; C. Twist; M. Daniels; J. Highton	2016	The Relationship Between Match-Play Characteristics of Elite Rugby League and Indirect Markers of Muscle Damage	International Journal of Sports Physiology and Performance	No	Abstract and keywords
1009	H. H. K. Fullagar; J. A. Sampson; J. Delaney; B. McKay; A. Murray	2019	The relationship between objective measures of sleep and training load across different phases of the season in American collegiate football players	Science & Medicine in Football	No	Abstract and keywords
1010	P. Jin; Z. Q. Zhao; X. F. Zhu	2023	The relationship between sport types, sex and visual attention as assessed in a multiple object tracking task	Frontiers in Psychology	No	Abstract and keywords
1011	R. A. Salman; L. J. Nemah; H. N. H. Alshawi	2020	THE RELATIONSHIP of PSYCHOLOGICAL TENSION with SOME CONCENTRATIONS of ANTIOXIDANTS among YOUNG HANDBALL PLAYERS	Annals of Tropical Medicine and Public Health	No	Abstract and keywords
1012	A. M. Hassan; A. H. Ali; H. N. H. Alshawi	2020	The relationship of refereeing performance of the leading personality and thyroxin hormone level for football referees (First division)	Indian Journal of Forensic Medicine and Toxicology	No	Abstract and keywords
1013	J. L. Fox; C. J. O'Grady; A. T. Scanlan	2020	The Relationships Between External and Internal Workloads During Basketball Training and Games	International Journal of Sports Physiology & Performance	No	Abstract and keywords
1014	E. J. Sobolewski	2020	The Relationships between Internal and External Load Measures for Division I College Football Practice	Sports (2075-4663)	No	Abstract and keywords
1015	T. Han; W. Wang; Y. Kuroda; M. Mizuno	2022	The Relationships of Sleep Duration and Inconsistency With the Athletic Performance of Collegiate Soft Tennis Players	Frontiers in Psychology	No	Abstract and keywords
1016	H. Ben Chikha; B. Zoudji; A. Khacharem	2023	The role of coach's gaze guidance on memorization of tactical movements in basketball: an eye tracking study	GERMAN JOURNAL OF EXERCISE AND SPORT RESEARCH	No	Abstract and keywords
1017	R. Izzo; T. D'isanto; G. Raiola; A. Cejudo; N. Ponsano; C. H. Varde'i	2020	The role of fatigue in football matches, performance model analysis and evaluation during quarters using live global positioning system technology at 50hz	Sport Science	No	Abstract and keywords
1018	I. E. Ott; J. I. Santos	2020	The role of nutrition in the recovery of a basketball player	Nutricion Hospitalaria	No	Abstract and keywords
1019	S. Buzzelli	2020	The Sigma Test": A new methodology for evaluating a tennis player"	Coaching & Sport Science Review	No	Abstract and keywords
1020	J. R. Silva	2022	The soccer season: performance variations and evolutionary trends	PeerJ	No	Abstract and keywords
1021	N. Dobbin; A. Atherton; C. Hill	2022	The subjective task load responses and movement characteristics associated with purposefully designed games in junior Touch players	International Journal of Sports Science and Coaching	No	Abstract and keywords
1022		2019	The Tennis Magazine 100	Tennis	No	Abstract and keywords
1023	H. H. K. Fullagar; A. Govus; J. Hanisch; A. Murray	2017	The Time Course of Perceptual Recovery Markers After Match Play in Division I-A College American Football	International Journal of Sports Physiology & Performance	No	Abstract and keywords
1024	W. G. Hopkins; D. S. Rowlands; R. P. Lamberts	2016	The Triumph of Technology for Athletes at the 21st Annual Meeting of the European College of Sport Science	Sportscience	No	Abstract and keywords
1025	T. Huyghe; P. E. Alcaraz; J. Calleja-González; S. P. Bird	2022	The underpinning factors of NBA game-play performance: a systematic review (2001–2020)	Physician and Sportsmedicine	No	Abstract and keywords
1026	Ł. Rydzik; W. Wąsacz; T.	2023	The Use of Neurofeedback in Sports	Brain Sciences	No	Abstract and

	Ambroży; N. Javdaneh; K. Brydak; M. Kopańska		Training: Systematic Review			keywords
1027	A. Adrian; E.-V. Virgil	2023	THEORETICAL CONCEPTS IN THE EFFICIENCY OF BALL HITTING TECHNIQUE IN THE GAME OF TENNIS	Ovidius University Annals, Series Physical Education & Sport/Science, Movement & Health	No	Abstract and keywords
1028	H. Otani; T. Goto; H. Goto; M. Shirato	2017	Time-of-day effects of exposure to solar radiation on thermoregulation during outdoor exercise in the heat	Chronobiology International	No	Abstract and keywords
1029		2017	Tomas Berdych	Tennis	No	Abstract and keywords
1030	D. Meffert; J. Breuer; L. Ohlendorf; P. Born; R. Grambow; T. Vogt	2021	Towards an understanding of big points in tennis: perspectives of coaches, professional players, and junior players	Journal of Physical Education & Sport	No	Abstract and keywords
1031	T. Fernandes; O. Camerino; M. Castañer	2021	T-Pattern Detection and Analysis of Football Players' Tactical and Technical Defensive Behaviour Interactions: Insights for Training and Coaching Team Coordination	Frontiers in Psychology	No	Abstract and keywords
1032	M. R. Román; J. García-Rubio; S. Feu; S. J. Ibáñez	2019	Training and competition load monitoring and analysis of women's amateur basketball by playing position: Approach study	Frontiers in Psychology	No	Abstract and keywords
1033	C. Gardner; J. W. Navalta; B. Carrier; C. Aguilar; J. Perdomo Rodriguez	2023	Training Impulse and Its Impact on Load Management in Collegiate and Professional Soccer Players	Technologies	No	Abstract and keywords
1034	D. H. Méndez; A. Pierobón; T. J. Gabbett	2022	Training Load Management in Professional Tennis Players During COVID-19 Lockdown: A Case Series Study	JOSPT Cases	No	Abstract and keywords
1035	W. Wolff; A. Bertrams; J. Schüler	2019	Trait self-control discriminates between youth football players selected and not selected for the german talent program: A Bayesian analysis	Frontiers in Psychology	No	Abstract and keywords
1036	K. Sivakumar; S. Sasikumar; M. Krishnamurthy	2023	Tree Social Relations Optimization-Based ReLU-BiLSTM Framework for Improving Video Quality in Video Compression	Journal of Circuits, Systems and Computers	No	Abstract and keywords
1037	M. S. Brink; A. W. Kersten; W. G. P. Frencken	2017	Understanding the mismatch between coaches' and players' perceptions of exertion	International Journal of Sports Physiology and Performance	No	Abstract and keywords
1038	F. R. Goes; L. A. Meerhoff; M. J. O. Bueno; D. M. Rodrigues; F. A. Moura; M. S. Brink; M. T. Elferink-Gemser; A. J. Knobbe; S. A. Cunha; R. S. Torres; K. A. P. M. Lemmink	2021	Unlocking the potential of big data to support tactical performance analysis in professional soccer: A systematic review	European Journal of Sport Science	No	Abstract and keywords
1039	J. Ferk	2019	Uporabnost in učinkovitost malih prirejenih iger v nogometu. / Applicability and effectiveness of small-sided games in football	Revija Šport	No	Abstract and keywords
1040	M. Noetel; J. Ciarrochi; B. Sahdra; C. Lonsdale	2019	Using genetic algorithms to abbreviate the Mindfulness Inventory for Sport: A substantive-methodological synthesis	Psychology of Sport and Exercise	No	Abstract and keywords
1041	S. J. Solomon; J. H. Batcherlor	2021	Vain or able? Strong inference and the efficacy debate	Team Performance Management	No	Abstract and keywords
1042	J. Schimpchen; P. F. Correia; T. Meyer	2023	Validity and reproducibility of match-derived ratios of selected external and internal load parameters in soccer players: A simple way to monitor physical fitness?	Biology of Sport	No	Abstract and keywords

1043	Y. Xie; B. Bai; Y. Zhao	2022	Variation Factors and Dynamic Modeling Analysis of Tennis Players' Competitive Ability Based on Big Data Mining Algorithm	Journal of Sensors	No	Abstract and keywords
1044	Z. Akyildiz; H. Nobari; F. T. González-Fernández; G. M. Praça; H. Sarmento; A. H. Guler; E. K. Saka; F. M. Clemente; A. J. Figueiredo	2022	Variations in the physical demands and technical performance of professional soccer teams over three consecutive seasons	Scientific Reports	No	Abstract and keywords
1045	D. Davidow; M. Watson; M. Lambert; B. Jones; M. Smith; W. Kraak; S. Hendricks	2023	Video-based technical feedback and instruction improves tackling technique of community rugby union players	European Journal of Sport Science	No	Abstract and keywords
1046	S. Klatt; N. J. Smeeton	2020	Visual and auditory information during decision making in sport	Journal of Sport and Exercise Psychology	No	Abstract and keywords
1047	S. Schaper; L. v. d. Kaaden; V. d. Boode; G. Savelsbergh	2020	Visual gaze behaviour during free-kicks in football	International Journal of Sports Science & Coaching	No	Abstract and keywords
1048	T. Hülsdünker; A. Mierau	2021	Visual Perception and Visuomotor Reaction Speed Are Independent of the Individual Alpha Frequency	Frontiers in Neuroscience	No	Abstract and keywords
1049	F. Dambroz; F. Cardoso; J. Afonso; I. Teoldo	2022	Visual search strategies of young soccer players according to positional role	Motricidade	No	Abstract and keywords
1050	C. Ballet; J. Barreto; E. Hope; F. Casanova	2023	What is the visual behaviour and attentional effort of football players in different positions during a real 11v11 game? A pilot study	F1000Research	No	Abstract and keywords
1051	M. A. Carron; A. T. Scanlan; C. J. Power; T. M. Doering	2023	What Tests are Used to Assess the Physical Qualities of Male, Adolescent Rugby League Players? A Systematic Review of Testing Protocols and Reported Data Across Adolescent Age Groups	Sports Medicine - Open	No	Abstract and keywords
1052	J. Higgins	2018	Why Roger Federer is a GOAT: an account of sporting genius	Journal of the Philosophy of Sport	No	Abstract and keywords
1053		2017	WINGING IT AGAINST THE LIONS	NZ Rugby World	No	Abstract and keywords
1054	S. Hemphill	2015	Winners yet again and again	NCAA News	No	Abstract and keywords
1055	M. IŞIk Afacan	2022	Zihinsel Hazır Oluş ile Skor Arasındaki İlişkinin Analizi: Gümüş Ligi Oturarak Voleybol Milli Takım Örneği. / Evaluation of the Relationship Between Mental Readiness and Score: Silver League Sitting Volleyball National Team Example	Mediterranean Journal of Sport Science (MJSS)	No	Abstract and keywords
1056	محمد .ک ;رسول .ز ;جواد .ن شهابی رضا	2023	افزایش را درونی توجه کانون اثربخشی پنهان یادگیری میدهد: اهمیت کاهش بار شناختی. / Implicit Learning Increases the Effectiveness of Internal Focus of Attention: The Importance of Reducing Cognitive Load	Journal of Sports & Motor Development & Learning	No	Abstract and keywords
1057	V. Vanessa Wergin; Z. Zimanyi; J. Beckmann	2021	A field study investigating running distance and affect of field hockey players in collective team collapse situations	International Journal of Sport and Exercise Psychology	No	Abstract and keywords
1058	M. Zanin; J. Ranaweera; J. Darrall-Jones; D. Weaving; K. Till; G. Roe	2021	A systematic review of small sided games within rugby: Acute and chronic effects of constraints manipulation	Journal of Sports Sciences	No	Abstract and keywords
1059	H. Silva; F. Y. Nakamura; M. Beato; R. Marcelino	2023	Acceleration and deceleration demands during training sessions in football: a	Science and Medicine in Football	No	Abstract and keywords

			systematic review			
1060	K. Davies; B. Staples; C. Morris	2020	Accommodate and adapt - Coaching in COVID-19 environment	Coaching & Sport Science Review (Spanish Version)	No	Abstract and keywords
1061	R. J. Mason; D. Farrow; J. A. C. Hattie	2020	An analysis of in-game feedback provided by coaches in an Australian Football League competition	Physical Education and Sport Pedagogy	No	Abstract and keywords
1062	Y. Takahashi	2015	Anserine as a suppressor of fatigue	Food and Nutritional Components in Focus	No	Abstract and keywords
1063	L. Pote; S. Nicholls; G. King; C. Christie	2023	Anthropometric and morphological characteristics of elite male cricket bowlers and batters over time: A systematic review	International Journal of Sports Science & Coaching	No	Abstract and keywords
1064	J. M. Tassi; M. A. López-Gajardo; J. Díaz-García; T. García-Calvo; I. González-Ponce	2023	Attentional focus in team sports: Effects of an intervention program on football players	European Journal of Human Movement	No	Abstract and keywords
1065	J. Vera; R. Molina; D. Cárdenas; B. Redondo; R. Jiménez	2020	Basketball free-throws performance depends on the integrity of binocular vision	European Journal of Sport Science	No	Abstract and keywords
1066	F. Carson; C. McCormack; P. McGovern; S. Ralston; J. Walsh	2021	Coach Like a Woman: Learnings From a Pilot Coach Education Program	Women in Sport & Physical Activity Journal	No	Abstract and keywords
1067	A. Harrison; A. Lane-Cordova; M. F. La Fountaine; R. D. Moore	2022	Concussion History and Heart Rate Variability During Bouts of Acute Stress	Journal of Athletic Training (Allen Press)	No	Abstract and keywords
1068	I. Popovych; I. Radul; V. Radul; I. Geiko; N. Hoi; O. Sribna; Y. Tymosh	2022	Construction and comparison of mental resource complexes of male and female sports teams	Journal of Physical Education and Sport	No	Abstract and keywords
1069	A. D. Hagstrom; K. A. Shorter	2018	Creatine kinase, neuromuscular fatigue, and the contact codes of football: A systematic review and meta-analysis of pre- and post-match differences	European Journal of Sport Science	No	Abstract and keywords
1070	T. J. Hepler; M. Andre	2021	Does stress type and level affect gut decisions in sport?	International Journal of Sport and Exercise Psychology	No	Abstract and keywords
1071	D. Mexis; T. Nomikos; N. Mitsopoulos; N. Kostopoulos	2023	Effect of a 6-Week Preseason Training Protocol on Physiological and Muscle Damage Markers in High-Level Female and Male Basketball Players	Sports	No	Abstract and keywords
1072	W. Abbott; C. Thomas; T. Clifford	2023	Effect of Playing Status and Fixture Congestion on Training Load, Mental Fatigue, and Recovery Status in Premier League Academy Goalkeepers	JOURNAL OF STRENGTH AND CONDITIONING RESEARCH	No	Abstract and keywords
1073	P. Goldman; B. Pandit; D. Gomez; S. Lu; C. Mills; N. Kull; R. Ku; A. Aramie; A. Kim; A. Alexandru; J. Hu; E. V. Neufeld; B. A. Dolezal	2022	Effect of Real-Time Feedback on Power Output Using a Novel Smart-Resisted Sled Push	International Journal of Exercise Science	No	Abstract and keywords
1074	I. M. Alkhawaldeh; M. Altarawneh	2023	Effect of Trait and State Anxiety on Overhead Defensive Clear Shot Skill Performance Regarding Some Kinematic Variables for Badminton Players	Asian Journal of Sports Medicine	No	Abstract and keywords
1075	T. N. Ziegenfuss; S. M. Habowski; R. Lemieux; J. E. Sandrock; A. W. Kedia; C. M. Kerksick; H. L. Lopez	2015	Effects of a dietary supplement on golf drive distance and functional indices of golf performance	Journal of the International Society of Sports Nutrition	No	Abstract and keywords

1076	W. Pramkratok; T. Yimlamai	2021	Effects of Acute Hypoxia on Psycho-Physiological Response and Muscle Oxygenation during Incremental Running Exercise	Journal of Exercise Physiology Online	No	Abstract and keywords
1077	A. S. Parodi-Feye; Á. D. Cappuccio-Díaz; C. A. Magallanes-Mira	2023	Effects of Inspiratory Muscle Training on Physiological Performance Variables in Women's Handball	Journal of Human Kinetics	No	Abstract and keywords
1078	L. S. Fortes; F. S. Fonseca; F. Y. Nakamura; B. T. Barbosa; P. Gantois; D. de Lima-Júnior; M. E. C. Ferreira	2021	Effects of Mental Fatigue Induced by Social Media Use on Volleyball Decision-Making, Endurance, and Countermovement Jump Performance	Perceptual and Motor Skills	No	Abstract and keywords
1079	A. Ghavami; H. Samadi; A. Dana; S. Ghorbani	2022	Effects of observing real, animated and combined model on learning cognitive and motor levels of basketball jump shot in children	Biomedical Human Kinetics	No	Abstract and keywords
1080	W. Guo; J. Ren; B. Y. Wang; Q. Zhu	2015	Effects of Relaxing Music on Mental Fatigue Induced by a Continuous Performance Task: Behavioral and ERPs Evidence	PLOS ONE	No	Abstract and keywords
1081	G. P. Nassis; A. Massey; P. Jacobsen; J. Brito; M. B. Randers; C. Castagna; M. Mohr; P. Krustrup	2020	Elite football of 2030 will not be the same as that of 2020: Preparing players, coaches, and support staff for the evolution	Scandinavian Journal of Medicine and Science in Sports	No	Abstract and keywords
1082	V. Richard; B. Lavoie-Léonard; T. Romeas	2021	Embedding Perceptual–Cognitive Training in the Athlete Environment: An Interdisciplinary Case Study Among Elite Female Goalkeepers Preparing for Tokyo 2020	Case Studies in Sport & Exercise Psychology	No	Abstract and keywords
1083	S. Lane; N. Hall; L. Keir; C. Lawrence	2020	Empowering Elite Athlete Education in Australian Professional Sports	International Journal of Sport & Society	No	Abstract and keywords
1084	P. Scott; R. Ahmun; C. de Weymarn; E. Gardner; A. Bliss; T. W. Jones; S. J. Callaghan; J. Tallent	2023	Evolution of anthropometric and physical performance characteristics of international male cricketers from 2014 to 2020 in a World Cup winning nation	International Journal of Sports Science and Coaching	No	Abstract and keywords
1085	Z. Bilić; F. Sinković; P. Barbaros; D. Novak; E. Zemkova	2023	Exercise-Induced Fatigue Impairs Change of Direction Performance and Serve Precision among Young Male Tennis Players	Sports	No	Abstract and keywords
1086	D. Farrow; T. Buszard	2017	Exploring the applicability of the contextual interference effect in sports practice	Prog Brain Res	No	Abstract and keywords
1087	N. A. Sothern; J. O'Gorman	2021	Exploring the mental health and wellbeing of professional academy footballers in England	Soccer and Society	No	Abstract and keywords
1088	A. J. Coutts	2016	Fatigue in football: it's not a brainless task!	Journal of Sports Sciences	No	Abstract and keywords
1089	N. Goldschmied; M. Raphaeli; S. Moothart; P. Furley	2022	Free throw shooting performance under pressure: a social psychology critical review of research	International Journal of Sport and Exercise Psychology	No	Abstract and keywords
1090	E. Galanis; A. Hatzigeorgiadis; N. Comoutos; F. Charachousi; X. Sanchez	2018	From the Lab to the Field: Effects of Self-Talk on Task Performance Under Distracting Conditions	Sport Psychologist	No	Abstract and keywords
1091	J. L. Fox; C. J. O'Grady; A. T. Scanlan	2020	Game schedule congestion affects weekly workloads but not individual game demands in semi-professional basketball	Biology of Sport	No	Abstract and keywords
1092	F. B. Mundstock; F. H. D. S. Maia; C. C. F. Bicalho	2021	Goal difference relationship between the national leagues of brazil, germany and england from the perspective of the prospect theory	Journal of Physical Education and Sport	No	Abstract and keywords
1093	J. M. Pratas; A.	2018	Goal scoring in elite male football: A	Journal of Human Sport &	No	Abstract and

	Volossovitch; A. I. Carita		systematic review	Exercise		keywords
1094	S. J. Haslerig	2017	Graduate(d) student athletes in Division I football: Redefining archetypes and disrupting stereotypes or invisible?	Sociology of Sport Journal	No	Abstract and keywords
1095	A. Rusdiana; A. Komaini; S. Nugraha	2023	Impact of cardiovascular fatigue on kinematic changes in badminton overhead jump smash: A descriptive analysis	Journal of Physical Education & Sport	No	Abstract and keywords
1096	H. R. Thornton; J. Miller; L. Taylor; C. Sargent; M. Lastella; P. M. Fowler	2018	Impact of short- compared to long-haul international travel on the sleep and wellbeing of national wheelchair basketball athletes	Journal of Sports Sciences	No	Abstract and keywords
1097	Y. Le Mansec; J. Perez; Q. Rouault; J. Doron; M. Jubeau	2020	Impaired performance of the smash stroke in badminton induced by muscle fatigue	International Journal of Sports Physiology and Performance	No	Abstract and keywords
1098	M. Peralta-Geis; J. Arboix-Alió; J. C. Sanromà; R. M. Agulled	2021	Influence of fatigue in decision-making in football referees	Sport TK	No	Abstract and keywords
1099	R. FernÁNdez; F. Zurita; M. A. R. Cepero; V. A. Molina; J. M. Vilches; J. Ambris	2015	Influence of the physical and psychological variables on physical injuries in football	Journal of Human Sport & Exercise	No	Abstract and keywords
1100	A. Peeters; C. Carling; J. Piscione; M. Lacome	2019	In-Match Physical Performance Fluctuations in International Rugby Sevens Competition	Journal of Sports Science & Medicine	No	Abstract and keywords
1101	I. Popovych; A. Kurova; I. Koval; V. Kazibekova; M. Maksymov; V. Huzar	2022	Interdependence of emotionality, anxiety, aggressiveness and subjective control in handball referees before the beginning of a game: a comparative analysis	Journal of Physical Education and Sport	No	Abstract and keywords
1102	W. K. Lam; J. X. Fan; Y. Zheng; W. C. C. Lee	2019	Joint and plantar loading in table tennis topspin forehand with different footwork	European Journal of Sport Science	No	Abstract and keywords
1103	S. H. Doeven; M. S. Brink; B. C. H. Huijgen; J. de Jong; K. A. P. M. Lemmink	2021	Managing load to optimize well-being and recovery during short-term match congestion in elite basketball	International Journal of Sports Physiology and Performance	No	Abstract and keywords
1104	L. W. Hogarth; B. J. Burkett; M. R. McKean	2016	Match demands of professional rugby football codes: A review from 2008 to 2015	International Journal of Sports Science and Coaching	No	Abstract and keywords
1105	J. M. Sarabia; J. Fernandez-Fernandez; C. Juan-Recio; H. Hernández-Davó; T. Urbán; M. Moya	2015	Mechanical, Hormonal and Psychological Effects of a Non-Failure Short-Term Strength Training Program in Young Tennis Players	Journal of Human Kinetics	No	Abstract and keywords
1106	S. Shao; C. Yu; Y. Song; J. S. Baker; U. C. Ugbolue; I. M. Lanzoni; Y. Gu	2020	Mechanical character of lower limb for table tennis cross step maneuver	International Journal of Sports Science and Coaching	No	Abstract and keywords
1107	Y. P. Da Costa; L. Fortes; R. Santos; E. Souza; L. Hayes; E. Soares-Silva; G. R. Batista	2023	Mental fatigue measured in real-world sport settings: A case study of world class beach volleyball players	Journal of Physical Education and Sport	No	Abstract and keywords
1108	J. Habay; M. Proost; J. De Wachter; J. Díaz-García; K. De Pauw; R. Meeusen; J. Van Cutsem; B. Roelands	2021	Mental Fatigue-Associated Decrease in Table Tennis Performance: Is There an Electrophysiological Signature?	Int J Environ Res Public Health	No	Abstract and keywords
1109	H. Makaruk; J. M. Porter; A. Bodasińska; S. Palmer	2020	Optimizing the penalty kick under external focus of attention and autonomy support instructions	European Journal of Sport Science	No	Abstract and keywords
1110	R. E. Venter	2014	Perceptions of team athletes on the importance of recovery modalities	European Journal of Sport Science	No	Abstract and keywords

1111	N. Maraga; R. Duffield; D. Gescheit; T. Perri; M. Reid	2018	Playing not once, not twice but three times in a day: the effect of fatigue on performance in junior tennis players	International Journal of Performance Analysis in Sport	No	Abstract and keywords
1112	T. Shcherbak; I. Popovych; A. Kariyev; A. Duisenbayeva; V. Huzar; I. Hoian; K. Kyrychenko	2023	Psychological causes of fatigue in football players	Journal of Physical Education and Sport	No	Abstract and keywords
1113	D. L. Huber; D. G. Thomas; M. Danduran; T. B. Meier; M. A. McCrea; L. D. Nelson	2019	Quantifying activity levels after sport-related concussion using actigraph and mobile (mHealth) technologies	Journal of Athletic Training	No	Abstract and keywords
1114	D. Conte; M. R. Smith; F. Santolamazza; T. G. Favero; A. Tessitore; A. Coutts	2019	Reliability, usefulness and construct validity of the Combined Basketball Skill Test (CBST)	Journal of Sports Sciences	No	Abstract and keywords
1115	M. Arvinen-Barrow; W. V. Massey; B. Hemmings	2014	Role of Sport Medicine Professionals in Addressing Psychosocial Aspects of Sport-Injury Rehabilitation: Professional Athletes' Views	Journal of Athletic Training (Allen Press)	No	Abstract and keywords
1116	I. Jukic; G. Julio; F. Cos; F. Cuzzolin; J. Olmo; N. Terrados; N. Njaradi; R. Sassi; B. Requena; L. Milanovic; I. Krakan; K. Chatzichristos; P. E. Alcaraz	2020	Strategies and Solutions for Team Sports Athletes in Isolation due to COVID-19	SPORTS	No	Abstract and keywords
1117	F. Audrino	2020	Strongest team favoritism in European national football: Myth or reality?	International Journal of Sport Finance	No	Abstract and keywords
1118	A. Francesco	2020	Strongest Team Favoritism in European National Football: Myth or Reality?	International Journal of Sport Finance	No	Abstract and keywords
1119	R. J. Jansen; R. Van Egmond; H. De Ridder	2016	Task prioritization in dual-tasking: Instructions versus preferences	PLoS ONE	No	Abstract and keywords
1120	P. Sansone; A. Tessitore; I. Lukonaitiene; H. Paulauskas; H. Tschan; D. Conte	2020	Technical-tactical profile, perceived exertion, mental demands and enjoyment of different tactical tasks and training regimes in basketball small-sided games	Biology of Sport	No	Abstract and keywords
1121	G. A. De Moraes; V. N. Soares; J. G. C. Chiminazzo	2022	TEMPORAL ANALYSIS OF GOALS SCORED IN FUTSAL: A COMPARISON OF TWO MODELS	Human Movement	No	Abstract and keywords
1122	H. S. Ahmed; S. M. Marcora; D. Dixon; G. Davison	2020	The Effect of a Competitive Futsal Match on Psychomotor Vigilance in Referees	International Journal of Sports Physiology & Performance	No	Abstract and keywords
1123	R. O'Meagher; J. O'Reilly; A. Ali	2022	The effect of different playing surfaces on soccer skill performance	International Journal of Sports Science and Coaching	No	Abstract and keywords
1124	G. Rekik; N. Khacharem; Y. Belkhir; N. Bali; M. Jarraya	2019	The effect of visualization format and content complexity on acquisition of tactical actions in basketball	LEARNING AND MOTIVATION	No	Abstract and keywords
1125	H. Folgado; R. Duarte; P. Marques; J. Sampaio	2015	The effects of congested fixtures period on tactical and physical performance in elite football	Journal of Sports Sciences	No	Abstract and keywords
1126	L. Nielsen	2014	The Games Approach and High School Football	Sport Journal	No	Abstract and keywords
1127	J. D. Graham; B. Zhang; D. M. Y. Brown; J. Cairney	2022	The Home Advantage in the National Basketball Association Conference Finals and Finals Series From 1979 to 2019: A Mediation Analysis of Offensive and Defensive Skills	Journal of Sport and Exercise Psychology	No	Abstract and keywords
1128	R. A. Denardi; F. A. R.	2017	The influence of defender's positional gap on	Journal of Human Sport and	No	Abstract and

	Clavijo; T. A. C. De Oliveira; S. L. Da Silva; B. Travassos; U. C. Corrêa		the aces in the sport of volleyball	Exercise		keywords
1129	K. Berestetska	2020	The Relationship Between Perceived Coaching Behaviors, Intrinsic Motivation, and Scholarship Status on NCAA Division I Tennis Players' Sport Commitment	Journal of Sport Behavior	No	Abstract and keywords
1130	D. Suarez; J. C. Rius	2016	The specific nature of endurance training in tennis	Coaching & Sport Science Review	No	Abstract and keywords
1131	R. Notario-Alonso; A. Prieto-Ayuso; A. GarcÍA-Notario; O. R. Contreras-JordÁN	2023	The sports commitment in football players and its relationship with the coach performance: A systematic review	Journal of Human Sport & Exercise	No	Abstract and keywords
1132	F. R. Goes; M. S. Brink; M. T. Elferink-Gemser; M. Kempe; K. A. P. M. Lemmink	2021	The tactics of successful attacks in professional association football: large-scale spatiotemporal analysis of dynamic subgroups using position tracking data	Journal of Sports Sciences	No	Abstract and keywords
1133	R. Marques; F. Martins; R. Mendes; M. C. E Silva; G. Dias	2018	The use of eye tracking glasses in basketball shooting: A systematic review	Journal of Physical Education and Sport	No	Abstract and keywords
1134	P. Camacho; D. A. Cruz; I. Madinabeitia; F. J. Giménez; D. Cárdenas	2021	Time Constraint Increases Mental Load and Influences in the Performance in Small-Sided Games in Basketball	Research Quarterly for Exercise and Sport	No	Abstract and keywords
1135	C. J. Thompson; M. Noon; C. Towlson; J. Perry; A. J. Coutts; L. D. Harper; S. Skorski; M. R. Smith; S. Barrett; T. Meyer	2020	Understanding the presence of mental fatigue in English academy soccer players	JOURNAL OF SPORTS SCIENCES	No	Abstract and keywords
1136	K. Put; J. Wagemans; J. Spitz; A. M. Williams; W. F. Helsen	2016	Using web-based training to enhance perceptual-cognitive skills in complex dynamic offside events	Journal of Sports Sciences	No	Abstract and keywords
1137	V. H. de Freitas; S. d. P. Ramos; A. Leicht; T. Alves; F. Rabelo; M. G. Bara-Filho; F. A. Guarnier; F. Y. Nakamura	2017	Validation of the futsal-specific intermittent shuttle protocol for the simulation of the physical demands of futsal match-play	International Journal of Performance Analysis in Sport	No	Abstract and keywords
1138	W. Jang; D. H. Kwak; Y. J. Ko	2020	Vitalizing effect of athlete-drafting task in fantasy sports: the role of competitive goal-framing, involvement, and competitiveness trait	European Sport Management Quarterly	No	Abstract and keywords
1139	S. Cao; S. K. Geok; S. Roslan; S. Qian; H. Sun; S. K. Lam; J. Liu	2022	Mindfulness-Based Interventions for the Recovery of Mental Fatigue: A Systematic Review	International Journal of Environmental Research and Public Health	No	Review article
1140	S. Cao; S. K. Geok; S. Roslan; H. Sun; S. K. Lam; S. Qian	2022	Mental Fatigue and Basketball Performance: A Systematic Review	Frontiers in Psychology	No	Review article
1141	F. M. Clemente; R. Ramirez-Campillo; D. Castillo; J. Raya-González; A. F. Silva; J. Afonso; H. Sarmento; T. Rosemann; B. Knechtle	2021	Effects of Mental Fatigue in Total Running Distance and Tactical Behavior During Small-Sided Games: A Systematic Review With a Meta-Analysis in Youth and Young Adult's Soccer Players	Frontiers in Psychology	No	Review article
1142	Y. P. da Costa; C. Freitas-Júnior; D. de Lima-Júnior; E. L. Soares-Silva; G. R. Batista; L. Hayes; L. de	2022	Mental fatigue and ball sports: a narrative review focused on physical, technical, and tactical performance	Motriz. Revista de Educacao Fisica	No	Review article

	Sousa Fortes					
1143	J. Fuster; T. Caparrós; L. Capdevila	2021	Evaluation of cognitive load in team sports: literature review	PEERJ	No	Review article
1144	A. García-Giménez; F. Pradas de la Fuente; C. Castellar Otín; L. Carrasco Páez	2022	Performance Outcome Measures in Padel: A Scoping Review	International Journal of Environmental Research and Public Health	No	Review article
1145	S. González-Víllora; A. Prieto-Ayuso; F. Cardoso; I. Teoldo	2022	The role of mental fatigue in soccer: a systematic review	International Journal of Sports Science and Coaching	No	Review article
1146	J. Habay; R. Uylenbroeck; R. Van Droogenbroeck; J. De Wachter; M. Proost; B. Tassignon; K. De Pauw; R. Meeusen; N. Pattyn; J. Van Cutsem; B. Roelands	2023	Interindividual Variability in Mental Fatigue-Related Impairments in Endurance Performance: A Systematic Review and Multiple Meta-regression	Sports Medicine - Open	No	Review article
1147	A. Hatzigeorgiadis; E. Galanis	2017	Self-talk effectiveness and attention	CURRENT OPINION IN PSYCHOLOGY	No	Review article
1148	R. Hunte; S. B. Cooper; I. M. Taylor; M. E. Nevill; R. Boat	2021	The mechanisms underpinning the effects of self-control exertion on subsequent physical performance: a meta-analysis	INTERNATIONAL REVIEW OF SPORT AND EXERCISE PSYCHOLOGY	No	Review article
1149	M. A. Nabli; N. Ben Abdelkrim; M. S. Fessi; M. D. DeLang; W. Moalla; K. Chamari	2019	Sport science applied to basketball refereeing: a narrative review	Physician and Sportsmedicine	No	Review article
1150	M. Nédélec; S. Halson; A. Abaidia; S. Ahmaidi; G. Dupont	2015	Stress, Sleep and Recovery in Elite Soccer: A Critical Review of the Literature	SPORTS MEDICINE	No	Review article
1151	D. J. Paul; P. S. Bradley; G. P. Nassis	2015	Factors Affecting Match Running Performance of Elite Soccer Players: Shedding Some Light on the Complexity	INTERNATIONAL JOURNAL OF SPORTS PHYSIOLOGY AND PERFORMANCE	No	Review article
1152	B. Reid; K. Schreiber; J. Shawhan; E. Stewart; R. Burch; W. Reimann	2020	Reaction time assessment for coaching defensive players in NCAA division 1 American football: A comprehensive literature review	International Journal of Industrial Ergonomics	No	Review article
1153	I. Rollo; C. Williams	2023	Carbohydrate Nutrition and Skill Performance in Soccer	SPORTS MEDICINE	No	Review article
1154	H. Sun; K. G. Soh; S. Roslan; M. Wazir; F. Liu; Z. Zhao	2022	The Counteractive Effect of Self-Regulation-Based Interventions on Prior Mental Exertion: A Systematic Review of Randomised Controlled Trials	BRAIN SCIENCES	No	Review article
1155	H. Sun; K. G. Soh; S. Roslan; M. R. W. N. Wazir; K. L. Soh	2021	Does mental fatigue affect skilled performance in athletes? A systematic review	PLoS ONE	No	Review article
1156	H. Sun; K. G. G. Soh; S. Roslan; M. Wazir; A. Mohammadi; C. Ding; Z. J. Zhao	2022	Nature exposure might be the intervention to improve the self-regulation and skilled performance in mentally fatigue athletes: A narrative review and conceptual framework	FRONTIERS IN PSYCHOLOGY	No	Review article
1157	C. J. Thompson; J. Fransen; S. Skorski; M. R. Smith; T. Meyer; S. Barrett; A. J. Coutts	2019	Mental Fatigue in Football: Is it Time to Shift the Goalposts? An Evaluation of the Current Methodology	Sports Medicine	No	Review article
1158	R. Yuan; H. Sun; K. G. Soh; A. Mohammadi; Z. Toumi; Z. Zhang	2023	The effects of mental fatigue on sport-specific motor performance among team sport athletes: A systematic scoping review	Frontiers in Psychology	No	Review article

1159	X. E. Fernandez; M. Priego-Ojeda; A. R. Morente; C. A. Mora	2022	Relationship between emotional intelligence, burnout and health perception in a sample of football Spanish referees	RETOS-NUEVAS TENDENCIAS EN EDUCACION FISICA DEPORTE Y RECREACION	No	No players
1160	M. Peralta-Geis; J. Arboix-Alió; J. C. Sanromà; R. M. Agulled	2021	Influence of fatigue in decision-making in football referees	Sport TK	No	No players
1161	R. D. Samuel; C. Englert; Q. Zhang; I. Basevitch	2018	Hi ref, are you in control? Self-control, ego-depletion, and performance in soccer referees	Psychology of Sport and Exercise	No	No players
1162	W. Abbott; T. E. Brownlee; R. J. Naughton; T. Clifford; R. Page; L. D. Harper	2020	Changes in perceptions of mental fatigue during a season in professional under-23 English Premier League soccer players	Research in Sports Medicine	No	No MFinduced
1163	D. B. Alder; D. P. Broadbent; J. Stead; J. Poolton	2019	The impact of physiological load on anticipation skills in badminton: From testing to training	Journal of Sports Sciences	No	No MFinduced
1164	D. B. Alder; P. R. Ford; J. Causer; A. M. Williams	2018	The effect of anxiety on anticipation, allocation of attentional resources, and visual search behaviours	Human Movement Science	No	No MFinduced
1165	H. Aliyari; H. Sahraei; M. Erfani; M. Mohammadi; M. Kazemi; M. R. Daliri; B. Minaei-Bidgoli; H. Agaei; M. Sahraei; S. M. Ali Seyed Hosseini; E. Tekieh; M. Salehi; F. Farajdokht	2020	Changes in cognitive functions following violent and football video games in young male volunteers by studying brain waves	Basic and Clinical Neuroscience	No	No MFinduced
1166	D. Aras; A. S. Onlu; T. Durmus; C. Cengiz; D. Guler; Y. Guler; A. Ugurlu; M. I. Aldhahi; M. Gnln	2023	A brief body scan mindfulness practice has no positive effect on the recovery of heart rate variability and cognitive tasks in female professional basketball players	FRONTIERS IN PSYCHOLOGY	No	No MFinduced
1167	M. A. Baldacchino; R. Kerr-Cumbo; E. ŞEnel	2023	A Quantitative Study on the Relationship Between Players' Wellness, Mental and Physical Intensity in Youth Football Training in Malta. / Malta'da Genç Futbol Antrenmanlarında Oyuncuların Wellness, Mental ve Fiziksel Yoğunluğu Arasındaki İlişki Üzerine Nicel Bir Çalışma	Mediterranean Journal of Sport Science (MJSS)	No	No MFinduced
1168	J. C. M. Barte; A. Nieuwenhuys; S. A. E. Geurts; M. A. J. Kompier	2019	Motivation counteracts fatigue-induced performance decrements in soccer passing performance	JOURNAL OF SPORTS SCIENCES	No	No MFinduced
1169	J. C. M. Barte; A. Nieuwenhuys; S. A. E. Geurts; M. A. J. Kompier	2020	Effects of fatigue on interception decisions in soccer	INTERNATIONAL JOURNAL OF SPORT AND EXERCISE PSYCHOLOGY	No	No MFinduced
1170	H. Ben Chikha; B. Zoudji; A. Khacharem	2023	Using pointing gestures to convey tactical information: investigating the roles of expertise and complexity	Psychological Research	No	No MFinduced
1171	H. Ben Chikha; B. Zoudji; A. Khacharem	2023	The role of coach’s gaze guidance on memorization of tactical movements in basketball: an eye tracking study	German Journal of Exercise and Sport Research	No	No MFinduced
1172	H. Ben Chikha; B. Zoudji; A. Khacharem	2023	An eye-tracking study of how coach's nonverbal communication affects memorization of basketball tactical scenes	International Journal of Sports Science and Coaching	No	No MFinduced
1173	S. Brini; A. Ben Abderrahman; C. C. T. Clark; S. Zouita; A. C.	2021	Sex-specific effects of small-sided games in basketball on psychometric and physiological markers during Ramadan	BMC SPORTS SCIENCE MEDICINE AND REHABILITATION	No	No MFinduced

	Hackney; K. Govindasamy; U. Granacher; H. Zouhal		intermittent fasting: a pilot study			
1174	D. P. Broadbent; J. Causer; A. Mark Williams; P. R. Ford	2017	The role of error processing in the contextual interference effect during the training of perceptual-cognitive skills	Journal of Experimental Psychology: Human Perception and Performance	No	No MFinduced
1175	M. J. Campbell; A. P. Moran; N. Bargary; S. Surmon; L. Bressan; I. C. Kenny	2019	Pupillometry during golf putting: A new window on the cognitive mechanisms underlying quiet eye	Sport, Exercise, and Performance Psychology	No	No MFinduced
1176	E. Carnegie; D. Marchant; S. Towers; P. Ellison	2020	Beyond visual fixations and gaze behaviour. Using pupillometry to examine the mechanisms in the planning and motor performance of a golf putt	Human Movement Science	No	No MFinduced
1177	H. J. Carson; D. Collins; J. Richards	2014	Intra-individual movement variability during skill transitions: A useful marker?	European Journal of Sport Science	No	No MFinduced
1178	H. J. Carson; D. Collins; J. Richards	2016	Initiating technical refinements in high-level golfers: Evidence for contradictory procedures	European Journal of Sport Science	No	No MFinduced
1179	F. Casanova; P. T. Esteves; M. B. Padilha; J. Ribeiro; A. M. Williams; J. Garganta	2022	The Effects of Physiological Demands on Visual Search Behaviours During 2 vs. 1 + GK Game Situations in Football: An in-situ Approach	Frontiers in Psychology	No	No MFinduced
1180	F. Casanova; I. Teoldo da Costa; M. Padilha; S. González-Víllora; J. Garganta	2022	The gaze behaviours and defensive tactical performance of football players during small-sided games: A pilot study. / Os comportamentos visuais e a performance tática defensiva dos futebolistas durante os jogos reduzidos: Estudo piloto	Revista Portuguesa de Ciências do Desporto	No	No MFinduced
1181	T. T. J. Chong; M. A. J. Apps; K. Giehl; S. Hall; C. H. Clifton; M. Husain	2018	Computational modelling reveals distinct patterns of cognitive and physical motivation in elite athletes	SCIENTIFIC REPORTS	No	No MFinduced
1182	A. J. Cocks; R. C. Jackson; D. T. Bishop; A. M. Williams	2016	Anxiety, anticipation and contextual information: A test of attentional control theory	Cognition and Emotion	No	No MFinduced
1183	D. R. Coimbra; G. G. Bevilacqua; F. S. Pereira; A. Andrade	2021	Effect of Mindfulness Training on Fatigue and Recovery in Elite Volleyball Athletes: A Randomized Controlled Follow-Up Study	JOURNAL OF SPORTS SCIENCE AND MEDICINE	No	No MFinduced
1184	E. K. Coughlan; A. Mark Williams; P. R. Ford	2019	Lessons from the experts: The effect of a cognitive processing intervention during deliberate practice of a complex task	Journal of Sport and Exercise Psychology	No	No MFinduced
1185	J. O. C. Coyne; A. J. Coutts; R. U. Newton; G. G. Haff	2021	The Influence of Mental Fatigue on Sessional Ratings of Perceived Exertion in Elite Open and Closed Skill Sports Athletes	Journal of Strength and Conditioning Research	No	No MFinduced
1186	J. O. C. Coyne; G. Gregory Haff; A. J. Coutts; R. U. Newton; S. Nimphius	2018	The current state of subjective training load monitoring—A practical perspective and call to action	Sports Medicine - Open	No	No MFinduced
1187	B. D. Daub; B. D. McLean; A. D. Heishman; A. J. Coutts	2023	The reliability and usefulness of a novel basketball standardized shooting task	INTERNATIONAL JOURNAL OF SPORTS SCIENCE & COACHING	No	No MFinduced
1188	B. D. Daub; B. D. McLean; A. D. Heishman; K. M. Peak; A. J. Coutts	2023	The Relationship Between Mental Fatigue and Shooting Performance Over the Course of a National Collegiate Athletic Association Division I Basketball Season	J Strength Cond Res	No	No MFinduced
1189	G. A. de Moraes; J. D. Crippa; A. F. Leme; J. G. C. Chiminazzo	2020	Futsal 2018 national league: analysis of the gols incidence	REVISTA BRASILEIRA DE FUTSAL E FUTEBOL	No	No MFinduced

1190	J. Díaz-García; J. C. Ponce-Bordón; A. Moreno-Gil; A. Rubio-Morales; M. Á. López-Gajardo; T. García-Calvo	2023	Influence of Scoring Systems on Mental Fatigue, Physical Demands, and Tactical Behavior during Soccer Large-Sided Games	International Journal of Environmental Research and Public Health	No	No MFinduced
1191	J. Díaz-García; J. J. Pulido; J. C. Ponce-Bordón; C. Cano-Prado; M. A. López-Gajardo; T. García-Calvo	2021	Coach Encouragement During Soccer Practices Can Influence Players' Mental and Physical Loads	JOURNAL OF HUMAN KINETICS	No	No MFinduced
1192	B. I. Dragoş; O. B. Marian	2016	STUDY ON THE STREAMLINE OF THE U15 WOMENS BASKETBALL FREE THROWS	Ovidius University Annals, Series Physical Education & Sport/Science, Movement & Health	No	No MFinduced
1193	B. D. Fletcher; C. Twist; J. D. Haigh; C. Brewer; J. P. Morton; G. L. Close	2016	Season-long increases in perceived muscle soreness in professional rugby league players: role of player position, match characteristics and playing surface	JOURNAL OF SPORTS SCIENCES	No	No MFinduced
1194	H. H. K. Fullagar; S. Skorski; R. Duffield; R. Julian; J. Bartlett; T. Meyer	2016	Impaired sleep and recovery after night matches in elite football players	Journal of Sports Sciences	No	No MFinduced
1195	Y. Gok; E. Suel; Y. Soylu	2023	Effects of different small-sided games on psychological responses and technical activities in young female basketball players	Acta Gymnica	No	No MFinduced
1196	N. Goldschmied; D. Vira	2019	The experience of ego threat in the public arena: A study of air ball shots performance in collegiate basketball	Journal of Applied Social Psychology	No	No MFinduced
1197	B. Gonçalves; D. Coutinho; B. Travassos; H. Folgado; P. Caixinha; J. Sampaio	2018	Speed synchronization, physical workload and match-to-match performance variation of elite football players	PLoS ONE	No	No MFinduced
1198	N. Haller; T. Ehlert; S. Schmidt; D. Ochmann; B. Sterzing; F. Grus; P. Simon	2019	Circulating, cell-free DNA for monitoring player load in professional football	International Journal of Sports Physiology and Performance	No	No MFinduced
1199	M. M. Hassan; N. F. Hassan; A. M. J. Al-Sayegh; H. N. H. Alshawi	2020	Awareness of creativity and its relationship to speed and accuracy of futsal soccer for female students	Indian Journal of Forensic Medicine and Toxicology	No	No MFinduced
1200	T. J. Hepler; A. J. Kovacs	2017	Influence of acute stress on decision outcomes and heuristics	Journal of Sports Medicine and Physical Fitness	No	No MFinduced
1201	R. J. Jansen; R. Van Egmond; H. De Ridder	2016	Task prioritization in dual-tasking: Instructions versus preferences	PLoS ONE	No	No MFinduced
1202	A. Khacharem	2017	Top-down and bottom-up guidance in comprehension of schematic football diagrams	Journal of Sports Sciences	No	No MFinduced
1203	A. Khacharem; K. Trabelsi; F. A. Engel; B. Sperlich; S. Kalyuga	2020	The Effects of Temporal Contiguity and Expertise on Acquisition of Tactical Movements	FRONTIERS IN PSYCHOLOGY	No	No MFinduced
1204	A. Khacharem; K. Trabelsi; B. Zoudji; S. Kalyuga	2020	Communicating Dynamic Behaviors in Basketball: The Role of Verbal Instructions and Arrow Symbols	Research Quarterly for Exercise and Sport	No	No MFinduced
1205	A. Khacharem; B. Zoudji; S. Kalyuga	2019	Which representation is best for communicating dynamic information?	Memory	No	No MFinduced
1206	A. Kok	2022	Cognitive control, motivation and fatigue: A	Brain and Cognition	No	No MFinduced

			cognitive neuroscience perspective			
1207	J. W. Lee; S. Song; Y. Kim; S. B. Park; D. H. Han	2023	Soccer's AI transformation: deep learning's analysis of soccer's pandemic research evolution	FRONTIERS IN PSYCHOLOGY	No	No MFinduced
1208	G. Marcolin; N. Camazzola; F. A. Panizzolo; D. Grigoletto; A. Paoli	2018	Different intensities of basketball drills affect jump shot accuracy of expert and junior players	PEERJ	No	No MFinduced
1209	Y. Mariano; K. Martin; J. Mara	2023	Mental fatigue and technical performance in elite rugby league	Journal of Sports Sciences	No	No MFinduced
1210	S. A. A. Massar; A. Csathó; D. Van der Linden	2018	Quantifying the Motivational Effects of Cognitive Fatigue Through Effort-Based Decision Making	FRONTIERS IN PSYCHOLOGY	No	No MFinduced
1211	E. Maunder; A. E. Kilding; S. P. Cairns	2017	Do fast bowlers fatigue in cricket? A paradox between player anecdotes and quantitative evidence	International Journal of Sports Physiology and Performance	No	No MFinduced
1212	J. Morente-Sánchez; T. Zandonai; M. Mateo-March; D. Sanabria; C. Sánchez-Muñoz; C. Chiamulera; M. Zabala Díaz	2015	Acute effect of Snus on physical performance and perceived cognitive load on amateur footballers	Scandinavian Journal of Medicine and Science in Sports	No	No MFinduced
1213	A. P. Murphy; R. Duffield; A. Kellett; M. Reid	2014	Comparison of athlete-coach perceptions of internal and external load markers for elite junior tennis training	International Journal of Sports Physiology and Performance	No	No MFinduced
1214	A. P. Murphy; R. Duffield; A. Kellett; M. Reid	2014	A descriptive analysis of internal and external loads for elite-level tennis drills	International Journal of Sports Physiology and Performance	No	No MFinduced
1215	A. P. Murphy; R. Duffield; A. Kellett; M. Reid	2016	A comparison of the perceptual and technical demands of tennis training, simulated match play, and competitive tournaments	International Journal of Sports Physiology and Performance	No	No MFinduced
1216	Y. Nagashima; K. Ehara; Y. Ehara; A. Mitsume; K. Kubo; S. Mineo	2023	Effects of Continuous Carbohydrate Intake with Gummies during the Golf Round on Interstitial Glucose, Golf Performance, and Cognitive Performance of Competitive Golfers: A Randomized Repeated-Measures Crossover Design	Nutrients	No	No MFinduced
1217	M. Nedelec; G. Dupont	2019	The influence of playing position in soccer on the recovery kinetics of cognitive and physical performance	JOURNAL OF SPORTS MEDICINE AND PHYSICAL FITNESS	No	No MFinduced
1218	B. Noël; S. Klatt	2021	Seemingly Uninvolved Players' Impact on Assistant Referees' Offside Decisions	JOURNAL OF SPORT & EXERCISE PSYCHOLOGY	No	No MFinduced
1219	S. Perrey	2023	Probing the Promises of Noninvasive Transcranial Electrical Stimulation for Boosting Mental Performance in Sports	Brain Sciences	No	No MFinduced
1220	S. A. Pullinger; P. S. Bradley; J. Causer; P. R. Ford; A. Newlove; K. Patel; K. Reid; C. M. Robertson; J. G. Burniston; D. A. Doran; J. M. Waterhouse; B. J. Edwards	2019	Football-induced fatigue in hypoxia impairs repeated sprint ability and perceptual-cognitive skills	SCIENCE AND MEDICINE IN FOOTBALL	No	No MFinduced
1221	G. Rekik; Y. Belkhir; M. Jarraya	2021	Searching to improve learning from complex animated basketball scenes: when decreasing the presentation speed is more efficient than using segmentation	Technology, Pedagogy and Education	No	No MFinduced

1222	G. Rekik; Y. Belkhir; M. Jarraya	2021	Improving Soccer Knowledge From Computerized Game Diagrams: Benefits of Sequential Instructional Arrows	Perceptual and Motor Skills	No	No MFinduced
1223	G. Rekik; Y. Belkhir; N. Mezghanni; M. Jarraya; Y. S. Chen; C. D. Kuo	2021	Learning basketball tactical actions from video modeling and static pictures: When gender matters	Children	No	No MFinduced
1224	G. Rekik; A. Khacharem; Y. Belkhir; N. Bali; M. Jarraya	2019	The effect of visualization format and content complexity on acquisition of tactical actions in basketball	Learning and Motivation	No	No MFinduced
1225	G. Rekik; A. Khacharem; Y. Belkhir; N. Bali; M. Jarraya	2019	The instructional benefits of dynamic visualizations in the acquisition of basketball tactical actions	Journal of Computer Assisted Learning	No	No MFinduced
1226	M. K. Robison; B. Nguyen	2023	Competition and reward structures nearly eliminate time-on-task performance decrements: Implications for theories of vigilance and mental effort	Journal of experimental psychology. Human perception and performance	No	No MFinduced
1227	O. R. Runswick; M. Jewiss; B. T. Sharpe; J. S. North	2021	Context affects quiet eye duration and motor performance independent of cognitive effort	Journal of Sport and Exercise Psychology	No	No MFinduced
1228	S. Russell; D. Jenkins; S. Halson; V. Kelly	2020	Changes in subjective mental and physical fatigue during netball games in elite development athletes	Journal of Science and Medicine in Sport	No	No MFinduced
1229	S. Russell; D. G. Jenkins; S. L. Halson; L. E. Juliff; M. J. Connick; V. G. Kelly	2022	Mental Fatigue Over 2 Elite Netball Seasons: A Case for Mental Fatigue to be Included in Athlete Self-Report Measures	International Journal of Sports Physiology and Performance	No	No MFinduced
1230	S. Russell; D. G. Jenkins; S. L. Halson; V. G. Kelly	2022	Mental fatigue increases across a 16-week pre-season in elite female athletes	Journal of Science and Medicine in Sport	No	No MFinduced
1231	S. Ryan; T. Kempton; F. M. Impellizzeri; A. J. Coutts	2020	Training monitoring in professional Australian football: theoretical basis and recommendations for coaches and scientists	Science and Medicine in Football	No	No MFinduced
1232	H. Sepahvand; G. P. Jahromi; H. Sahraei; G. H. Meftahi	2017	Studying the perceptive and cognitive function under the stress of match in female futsal players	Asian Journal of Sports Medicine	No	No MFinduced
1233	F. Silvestri; M. Campanella; M. Bertollo; M. R. Albuquerque; V. Bonavolontà; F. Perroni; C. Baldari; L. Guidetti; D. Curzi	2023	Acute Effects of Fitlight Training on Cognitive-Motor Processes in Young Basketball Players	International Journal of Environmental Research and Public Health	No	No MFinduced
1234	W. Staiano; M. Merlini; M. Romagnoli; U. Kirk; C. Ring; S. Marcora	2022	Brain Endurance Training Improves Physical, Cognitive, and Multitasking Performance in Professional Football Players	International Journal of Sports Physiology and Performance	No	No MFinduced
1235	J. M. Tassi; J. Díaz-García; M. A. López-Gajardo; A. Rubio-Morales; T. García-Calvo	2023	Effect of a Four-Week Soccer Training Program Using Stressful Constraints on Team Resilience and Precompetitive Anxiety	INTERNATIONAL JOURNAL OF ENVIRONMENTAL RESEARCH AND PUBLIC HEALTH	No	No MFinduced
1236	B. Thatcher; G. Ivanov; M. Szerovay; G. Mills	2021	Virtual Reality Technology in Football Coaching: Barriers and Opportunities	INTERNATIONAL SPORT COACHING JOURNAL	No	No MFinduced
1237	C. J. Thompson; M. Noon; C. Towlson; J. Perry; A. J. Coutts; L. D. Harper; S. Skorski; M. R. Smith; S. Barrett; T. Meyer	2020	Understanding the presence of mental fatigue in English academy soccer players	JOURNAL OF SPORTS SCIENCES	No	No MFinduced
1238	T. Toering; G. Jordet	2015	Self-Control in Professional Soccer Players	JOURNAL OF APPLIED SPORT PSYCHOLOGY	No	No MFinduced
1239	J. Vera; R. Jiménez; B. Redondo; I. Madinabeitia;	2019	Intraocular Pressure as an Indicator of the Level of Induced Anxiety in Basketball	OPTOMETRY AND VISION SCIENCE	No	No MFinduced

	I. Madinabeitia; F. A. López; D. Cárdenas					
1240	J. Zhu; Y. P. Zhu; G. Song	2023	Effect of Probiotic Yogurt Supplementation(<i>Bifidobacterium animalis</i> ssp. lactis BB-12) on Gut Microbiota of Female Taekwondo Athletes and Its Relationship with Exercise-Related Psychological Fatigue	MICROORGANISMS	No	No MFinduced
1241	I. I. M. Alkhawaldeh	2023	The Effect of Mental Fatigue on the Accuracy of the Direct Free Kick in Terms of Some Kinematic Variables for Football Players	Asian Journal of Sports Medicine	No	No recovery after MF induced
1242	L. Angius; M. Merlini; J. Hopker; M. Bianchi; F. Fois; F. Piras; P. Cugia; J. Russell; S. M. Marcora	2022	Physical and Mental Fatigue Reduce Psychomotor Vigilance in Professional Football Players	International Journal of Sports Physiology and Performance	No	No recovery after MF induced
1243	O. O. Badin; M. R. Smith; D. Conte; A. J. Coutts	2016	Mental fatigue: Impairment of technical performance in small-sided soccer games	International Journal of Sports Physiology and Performance	No	No recovery after MF induced
1244	T. Ballard; A. Neal; S. Farrell; E. Lloyd; J. Lim; A. Heathcote	2022	A General Architecture for Modeling the Dynamics of Goal-Directed Motivation and Decision-Making	PSYCHOLOGICAL REVIEW	No	No recovery after MF induced
1245	C. Bian; A. Ali; G. P. Nassis; Y. M. Li	2022	Repeated Interval Loughborough Soccer Passing Tests: An Ecologically Valid Motor Task to Induce Mental Fatigue in Soccer	FRONTIERS IN PHYSIOLOGY	No	No recovery after MF induced
1246	R. Boat; C. Sunderland; S. B. Cooper	2021	Detrimental effects of prior self-control exertion on subsequent sporting skill performance	Scandinavian Journal of Medicine and Science in Sports	No	No recovery after MF induced
1247	G. Ciocca; A. Tessitore; M. Mandorino; H. Tschan	2022	A Video-Based Tactical Task Does Not Elicit Mental Fatigue and Does Not Impair Soccer Performance in a Subsequent Small-Sided Game	Sports	No	No recovery after MF induced
1248	D. Coutinho; B. Gonçalves; B. Travassos; D. Wong; A. J. Coutts; J. E. Sampaio	2017	Mental Fatigue and Spatial References Impair Soccer Players' Physical and Tactical Performances	FRONTIERS IN PSYCHOLOGY	No	No recovery after MF induced
1249	D. Coutinho; B. Gonçalves; D. P. Wong; B. Travassos; A. J. Coutts; J. Sampaio	2018	Exploring the effects of mental and muscular fatigue in soccer players' performance	HUMAN MOVEMENT SCIENCE	No	No recovery after MF induced
1250	B. D. Daub; B. D. McLean; A. D. Heishman; K. M. Peak; A. J. Coutts	2023	Impacts of mental fatigue and sport specific film sessions on basketball shooting tasks	European Journal of Sport Science	No	No recovery after MF induced
1251	D. Davidow; M. Smith; T. Ross; G. L. James; L. Paul; M. Lambert; B. Jones; S. Hendricks	2023	Mental Fatigue Impairs Tackling Technique in Amateur Rugby Union Players	International Journal of Sports Physiology and Performance	No	No recovery after MF induced
1252	J. Díaz-García; V. J. Clemente-Suárez; J. P. Fuentes-García; S. Villafaina	2023	Combining HIIT Plus Cognitive Task Increased Mental Fatigue but Not Physical Workload in Tennis Players	Applied Sciences (Switzerland)	No	No recovery after MF induced
1253	C. Englert; A. Bertrams	2015	Autonomy as a protective factor against the detrimental effects of ego depletion on tennis serve accuracy under pressure	International Journal of Sport and Exercise Psychology	No	No recovery after MF induced
1254	C. Englert; A. Bertrams; P. Furley; R. R. D. Oudejans	2015	Is ego depletion associated with increased distractibility? Results from a basketball free throw task	Psychology of Sport and Exercise	No	No recovery after MF induced
1255	H. Faro; D. Cavalcante Silva; B. T. Barbosa; Y. P.	2023	Young Basketball Players' Multiple Object Tracking Skills Were Unaffected by	Perceptual and Motor Skills	No	No recovery after MF induced

	D. Costa; C. G. Freitas-Junior; D. de Lima-Junior; J. Faubert; L. D. S. Fortes		Stroop-Induced Mental Fatigue			
1256	H. Faro; L. D. S. Fortes; D. D. Lima-Junior; B. T. Barbosa; M. E. C. Ferreira; S. S. Almeida	2023	Sport-based video game causes mental fatigue and impairs visuomotor skill in male basketball players	International Journal of Sport and Exercise Psychology	No	No recovery after MF induced
1257	L. Filipas; D. Ferioli; G. Banfi; A. L. Torre; J. A. Vitale	2021	Single and combined effect of acute sleep restriction and mental fatigue on basketball free-throw performance	International Journal of Sports Physiology and Performance	No	No recovery after MF induced
1258	L. Filipas; C. Rossi; R. Codella; M. Bonato	2023	Mental Fatigue Impairs Second Serve Accuracy in Tennis Players	Research Quarterly for Exercise and Sport	No	No recovery after MF induced
1259	L. S. Fortes; G. P. Berriel; H. Faro; C. G. Freitas-Júnior; L. A. Peyré-Tartaruga	2022	Can Prolongate Use of Social Media Immediately Before Training Worsen High Level Male Volleyball Players' Visuomotor Skills?	Perceptual and Motor Skills	No	No recovery after MF induced
1260	L. S. Fortes; F. S. Fonseca; F. Y. Nakamura; B. T. Barbosa; P. Gantois; D. de Lima-Júnior; M. E. C. Ferreira	2021	Effects of Mental Fatigue Induced by Social Media Use on Volleyball Decision-Making, Endurance, and Countermovement Jump Performance	Perceptual and Motor Skills	No	No recovery after MF induced
1261	L. S. Fortes; D. Lima-Junior; B. T. Barbosa; H. K. C. Faro; M. E. C. Ferreira; S. S. Almeida	2022	Effect of mental fatigue on decision-making skill and visual search behaviour in basketball players: an experimental and randomised study	International Journal of Sport and Exercise Psychology	No	No recovery after MF induced
1262	J. P. Fuentes-García; J. Díaz-García; M. A. López-Gajardo; V. J. Clemente-Suarez	2021	Effects of Combined HIIT and Stroop on Strength Manifestations, Serve Speed and Accuracy in Recreational Tennis Players	SUSTAINABILITY	No	No recovery after MF induced
1263	G. Góes; R. A. Amaral; M. P. Morato	2021	Uefa champions league: overview of the critical phase of match, influence of the first goal and home advantage	REVISTA BRASILEIRA DE FUTSAL E FUTEBOL	No	No recovery after MF induced
1264	J. Habay; M. Proost; J. De Wachter; J. Díaz-garcía; K. De Pauw; R. Meeusen; J. Van Cutsem; B. Roelands	2021	Mental fatigue-associated decrease in table tennis performance: is there an electrophysiological signature?	International Journal of Environmental Research and Public Health	No	No recovery after MF induced
1265	M. H. Kosack; W. Staiano; R. Folino; M. B. Hansen; S. Lønbro	2020	The acute effect of mental fatigue on badminton performance in elite players	International Journal of Sports Physiology and Performance	No	No recovery after MF induced
1266	C. A. Kunrath; F. Cardoso; F. Y. Nakamura; I. Teoldo	2018	Mental fatigue as a conditioner of the tactical and Physical response in soccer players: A pilot study	Human Movement	No	No recovery after MF induced
1267	C. A. Kunrath; F. Y. Nakamura; A. Roca; A. Tessitore; I. Teoldo Da Costa	2020	How does mental fatigue affect soccer performance during small-sided games? A cognitive, tactical and physical approach	Journal of Sports Sciences	No	No recovery after MF induced
1268	Y. Le Mansec; B. Pageaux; A. Nordez; S. Dorel; M. Jubeau	2018	Mental fatigue alters the speed and the accuracy of the ball in table tennis	Journal of Sports Sciences	No	No recovery after MF induced
1269	A. Magdaleno; L. Wiersma; B. B. Meyer	2022	An Exploratory Comparison of Subjective Mental Fatigue Following a Task Designed to Replicate the Observation of Game Film	International Journal of Exercise Science	No	No recovery after MF induced
1270	M. Mikicin; M. Kowalczyk	2015	Audio-Visual and Autogenic Relaxation Alter Amplitude of Alpha EEG Band, Causing Improvements in Mental Work	APPLIED PSYCHOPHYSIOLOGY AND BIOFEEDBACK	No	No recovery after MF induced

			Performance in Athletes			
1271	A. Moreira; M. S. Aoki; E. Franchini; D. G. da Silva Machado; A. C. Paludo; A. H. Okano	2018	Mental fatigue impairs technical performance and alters neuroendocrine and autonomic responses in elite young basketball players	Physiology and Behavior	No	No recovery after MF induced
1272	A. Oliver; P. J. McCarthy; L. Burns	2020	A Grounded-Theory Study of Meta-Attention in Golfers	SPORT PSYCHOLOGIST	No	No recovery after MF induced
1273	E. M. Penna; E. Filho; B. T. Campos; D. A. Pires; F. Y. Nakamura; T. T. Mendes; T. R. Lopes; M. Smith; L. S. Prado	2018	Mental fatigue does not affect heart rate recovery but impairs performance in handball players	Revista Brasileira de Medicina do Esporte	No	No recovery after MF induced
1274	M. Shin; Y. Kim; S. Park	2019	Effects of State Anxiety and Ego Depletion on Performance Change in Golf Putting: A Hierarchical Linear Model Application	Perceptual and Motor Skills	No	No recovery after MF induced
1275	D. C. D. Silva; J. Afonso; D. Augusto; G. H. Petiot; C. C. Martins Filho; F. Vasconcellos	2023	Influence of pre-induced mental fatigue on tactical behaviour and performance among young elite football players	International Journal of Sport and Exercise Psychology	No	No recovery after MF induced
1276	M. R. Smith; A. J. Coutts; M. Merlini; D. Deprez; M. Lenoir; S. M. Marcora	2016	Mental fatigue impairs soccer-specific physical and technical performance	Medicine and Science in Sports and Exercise	No	No recovery after MF induced
1277	M. R. Smith; J. Fransen; D. Deprez; M. Lenoir; A. J. Coutts	2017	Impact of mental fatigue on speed and accuracy components of soccer-specific skills	Science and Medicine in Football	No	No recovery after MF induced
1278	M. R. Smith; L. Zeuwts; M. Lenoir; N. Hens; L. M. S. De Jong; A. J. Coutts	2016	Mental fatigue impairs soccer-specific decision-making skill	Journal of Sports Sciences	No	No recovery after MF induced
1279	J. Torrado; C. Arce; A. Vales-Vázquez; A. Areces; G. Iglesias; I. Valle; G. Patiño	2017	Relationship between Leadership among Peers and Burnout in Sports Teams	Spanish Journal of Psychology	No	No recovery after MF induced
1280	J. Van Cutsem; K. De Pauw; C. Vandervaeren; S. Marcora; R. Meeusen; B. Roelands	2019	Mental fatigue impairs visuomotor response time in badminton players and controls	Psychology of Sport and Exercise	No	No recovery after MF induced
1281	D. Veness; S. D. Patterson; O. Jeffries; M. Waldron	2017	The effects of mental fatigue on cricket-relevant performance among elite players	Journal of Sports Sciences	No	No recovery after MF induced
1282	J. Vera; R. Jiménez; J. A. García; D. Cárdenas	2017	Simultaneous Physical and Mental Effort Alters Visual Function	OPTOMETRY AND VISION SCIENCE	No	No recovery after MF induced
1283	T. Vogt; S. Gassen; S. Wrede; J. Spielmann; M. Jedrusiak-Jung; S. Hartel; J. Mayer	2018	Football practice with youth players in the Footbonaut" Speed of action and ball control in face of physical andmental strain"	GERMAN JOURNAL OF EXERCISE AND SPORT RESEARCH	No	No recovery after MF induced
1284	L. Yang; Y. C. Wang	2023	The effect of motivational and instructional self-talk on attentional control under noise distraction	PLOS ONE	No	No recovery after MF induced
1285	F. Alarcón; N. Ureña; D. Cárdenas	2017	Mental fatigue impairs the basketball free-throw performance	Revista de Psicologia del Deporte	No	Not English
1286	D. C. da Silva; D. M. Carnevale; D. A. N. Santos; C. de Novaes Andrade; C. C. M. Filho; F. Vasconcellos	2024	Mental fatigue in football: behavioural responses of players with high and low tactical performance	Retos	No	Not English
1287	T. García-Calvo; I. González-Ponce; J. C. Ponce; D. Tomé-Lourido;	2019	Incidence of the tasks scoring system on the mental load in football training	REVISTA DE PSICOLOGIA DEL DEPORTE	No	Not English

	A. Vales-Vázquez					
1288	C. Myojin; S. Ueshima; M. Kawanishi; M. Tokimoto; T. Matsunami; K. Sagawa; K. Yoshitani; T. Ioka; K. Omori	2016	Reducing Effects of branched-chain amino acids and the citric acid on fatigue caused by exercise	Japanese Pharmacology and Therapeutics	No	Not English
1289	N. S. Shumova; Y. V. Baykovsky; L. Siuntse	2019	Russian and Chinese student basketball teams: Personality profiles versus the competitive success rates	Teoriya i Praktika Fizicheskoy Kultury	No	Not English
1290	J. Habay; M. Proost; J. De Wachter; J. Díaz-García; K. De Pauw; R. Meeusen; J. Van Cutsem; B. Roelands	2023	Correction: Habay et al. Mental Fatigue-Associated Decrease in Table Tennis Performance: Is There an Electrophysiological Signature? Int. J. Environ. Res. Public Health 2021, 18, 12906	Int J Environ Res Public Health	No	Correction
1291	A. J. Coutts	2016	Fatigue in football: it's not a brainless task!	Journal of Sports Sciences	No	Not fulltext
1292	N. S. Weerakkody; C. J. Taylor; C. L. Bulmer; D. B. Hamilton; J. Gloury; N. J. O'Brien; J. H. Saunders; S. Harvey; T. A. Patterson	2021	The effect of mental fatigue on the performance of Australian football specific skills amongst amateur athletes	Journal of Science and Medicine in Sport	No	Not fulltext
1293	C. Englert; A. Bertrams	2016	Active relaxation counteracts the effects of ego depletion on performance under evaluative pressure in a state of ego depletion	Sportwissenschaft	Yes	
1294	L. S. Fortes; M. E. C. Ferreira; H. Faro; E. M. Penna; S. S. Almeida	2022	Brain Stimulation Over the Motion-Sensitive Midtemporal Area Reduces Deleterious Effects of Mental Fatigue on Perceptual-Cognitive Skills in Basketball Players	Journal of sport & exercise psychology	Yes	
1295	E. Galanis; L. Nurkse; J. Kooi; E. Papagiannis; A. Karathanasi; N. Comoutos; Y. Theodorakis; A. Hatzigeorgiadis	2022	Effects of a Strategic Self-Talk Intervention on Attention Functions and Performance in a Golf Task under Conditions of Ego Depletion	Sustainability (Switzerland)	Yes	
1296	A. Moreira; L. Moscaleski; D. G. D. Machado; M. Bikson; G. Unal; P. S. Bradley; T. Cevada; F. T. G. da Silva; A. F. Baptista; E. Morya; A. H. Okano	2023	Transcranial direct current stimulation during a prolonged cognitive task: the effect on cognitive and shooting performances in professional female basketball players	ERGONOMICS	Yes	
1297	F. Shaabani; A. Naderi; E. Borella; L. Calmeiro	2020	Does a brief mindfulness intervention counteract the detrimental effects of ego depletion in basketball free throw under pressure?	Sport, Exercise, and Performance Psychology	Yes	
1298	H. Sun; K. G. Soh; X. Xu	2022	Nature Scenes Counter Mental Fatigue-Induced Performance Decrements in Soccer Decision-Making	Front Psychol	Yes	

Literature information is generated by EndNote 20's Copy Formatted Literature feature. All authors will jointly decide on the treatment of missing data on a case-by-case basis. In terms of the practicalities of the literature screening process for this review, a small number of articles did have author information, but the rest of the information was complete and did not affect our screening. Only two studies in this systematic review were not available in full text. After a joint assessment by all authors, it was determined that the missing data would have no impact on the results of the analysis. Therefore, we chose to ignore this missing data.

Name of data extractors and date of data extraction

Author(s)	Year	Title	Name of data extractors	date
C. Englert; A. Bertrams	2016	Active relaxation counteracts the effects of ego depletion on performance under evaluative pressure in a state of ego depletion	Scopus	2024/1/2
L. S. Fortes; M. E. C. Ferreira; H. Faro; E. M. Penna; S. S. Almeida	2022	Brain Stimulation Over the Motion-Sensitive Midtemporal Area Reduces Deleterious Effects of Mental Fatigue on Perceptual-Cognitive Skills in Basketball Players	Scopus	2024/1/2
E. Galanis; L. Nurkse; J. Kooi; E. Papagiannis; A. Karathanasi; N. Comoutos; Y. Theodorakis; A. Hatzigeorgiadis	2022	Effects of a Strategic Self-Talk Intervention on Attention Functions and Performance in a Golf Task under Conditions of Ego Depletion	Scopus	2024/1/2
A. Moreira; L. Moscaleski; D. G. D. Machado; M. Bikson; G. Unal; P. S. Bradley; T. Cevada; F. T. G. da Silva; A. F. Baptista; E. Morya; A. H. Okano	2023	Transcranial direct current stimulation during a prolonged cognitive task: the effect on cognitive and shooting performances in professional female basketball players	Web of Science	2024/1/2
F. Shaabani; A. Naderi; E. Borella; L. Calmeiro	2020	Does a brief mindfulness intervention counteract the detrimental effects of ego depletion in basketball free throw under pressure?	Scopus	2024/1/2
H. Sun; K. G. Soh; X. Xu	2022	Nature Scenes Counter Mental Fatigue-Induced Performance Decrements in Soccer Decision-Making	Pubmed	2024/1/2

Literature information is generated by EndNote 20's Copy Formatted Literature feature.
